# A cell-cell communication signal from *Enterobacter cloacae* interfering with the signaling systems and virulence in *Shigella sonnei*

**DOI:** 10.1128/aem.00510-25

**Published:** 2025-05-12

**Authors:** Xiayu Chen, Mingfang Wang, Zhuoxian Zhao, Xiwen Ling, Ganjin Peng, Binbin Cui, Qiaoping Wang, Bing Gu, Yinyue Deng

**Affiliations:** 1 School of Pharmaceutical Sciences (Shenzhen), Sun Yat-sen University - Shenzhen Campus 582261 , Shenzhen, China; 2 Department of Clinical Laboratory Medicine, Guangdong Provincial People’s Hospital (Guangdong Academy of Medical Sciences), Southern Medical University, Guangzhou, Guangdong, China; 3 Pharmacy Department, The Affiliated LiHuiLi Hospital of Ningbo University, Ningbo, China; Indiana University Bloomington, Bloomington, Indiana, USA

**Keywords:** quorum sensing, *Enterobacter cloacae*, indole-3-ethanol, biofilm formation, virulence

## Abstract

Quorum sensing (QS) is widely utilized by both bacteria and fungi to mediate cell-cell communication. Previous studies have demonstrated that the indole derivative indole-3-ethanol (also known as tryptophol) controls morphogenesis as a QS molecule in fungi. However, whether this QS signal is involved in the modulation of biological functions in bacteria remains unknown. Here, we report that indole-3-ethanol controls the biological functions and pathogenicity of *Enterobacter cloacae* subsp. *cloacae* ATCC 13047. The biosynthesis of indole-3-ethanol is performed by YjgB (ECL_RS22935), an alcohol dehydrogenase. Deletion of *yjgB* results in impaired biological functions and virulence. Furthermore, we revealed that indole-3-ethanol from *E. cloacae* reduces the competitive fitness of *Shigella sonnei* by inhibiting its biofilm formation, extracellular polysaccharide synthesis, and virulence. Given that both *E. cloacae* and *S. sonnei* are common human intestinal microbes, our results highlight the critical roles of indole-3-ethanol in both intraspecies signaling and interspecies communication in bacteria.

**IMPORTANCE:**

Quorum sensing is a cell-cell communication mechanism widely employed by bacteria to control various biological functions and pathogenicity. In this study, we demonstrated that *Enterobacter cloacae* employs indole-3-ethanol as a quorum-sensing signal to control biological functions and virulence. We also revealed that indole-3-ethanol from *E. cloacae* effectively inhibits biofilm formation and virulence in *Shigella sonnei*. Our findings not only suggest the important role of indole-3-ethanol in the regulation of the pathogenicity of *E. cloacae* but also provide new insights into the development of indole-3-ethanol as an anti-virulence agent against *S. sonnei*.

## INTRODUCTION

Quorum sensing (QS) is a cell density-dependent cell-to-cell communication mechanism that coordinates a series of crucial physiological activities, such as biofilm formation, motility, virulence factor production, and antibiotic resistance in bacteria (1–3). One of the most well-studied QS systems in gram-negative bacteria is *N*-acyl-L-homoserine lactone (AHL), which plays a critical role in regulating various physiological processes (4, 5). In addition, many other QS signals have been identified, such as diffusible signaling factor (DSF) family signals (6–8), autoinducer-2 (AI-2) (9), 2-heptyl-3-hydroxy-4(1H)-quinolone (PQS) (10, 11), and anthranilic acid (12). Recently, the QS signal indole was demonstrated to be synthesized by the novel enzyme AbiS in *Acinetobacter baumannii* through a pathway that is quite distinct from the traditional tryptophanase route (13). AbiR has also been characterized as a receptor, exhibiting high affinity for indoles via a unique region between its receiver (REC) and helix-turn-helix (HTH) domains, which enhances its role in regulating key physiological and virulence functions (14).

In addition to bacteria, QS systems have also been identified in fungi, where they play crucial roles in coordinating collective behaviors such as morphogenesis and biofilm formation (15, 16). For example, farnesol acts as a QS molecule, inhibiting hyphal growth and controlling biofilm development in *Candida albicans*, whereas tyrosol has the opposite effect, promoting the yeast-to-hyphal transition essential for pathogenesis (17, 18). Additionally, indole-3-ethanol (also known as tryptophol) functions as a QS signal in *Saccharomyces cerevisiae*, stimulating morphogenesis by upregulating the expression of FLO11 through a Tpk2p-dependent pathway (19–22). In the marine yeast *Scheffersomyces spartinae*, indole-3-ethanol can promote its own population density, biofilm formation, and cell aggregation. It can also enhance the biocontrol effect of *S. spartinae* against *Botrytis cinerea* through QS (23). However, studies on the regulatory effects of indole-3-ethanol on the biological functions of bacteria are scarce, and further exploration is essential to uncover its potential mechanisms and roles.

*Enterobacter cloacae* is an opportunistic gram-negative pathogen that causes respiratory and urinary tract infections, particularly in immunocompromised individuals (24). The increase in antibiotic resistance, especially carbapenem resistance and extended-spectrum beta-lactamase production, has raised concerns about *E. cloacae* in health-care settings (25). Previous studies have shown that *trans* expression of a homoserine lactonase (AiiA), which hydrolyzes AHL signaling molecules, affects proteolytic activity and biofilm formation in *E. cloacae* (26). The AHL-dependent transcriptional regulator SdiA in *E. cloacae* GS1 can respond to exogenous AHLs during rice root colonization, and its inactivation enhances bacterial adhesion, root colonization, and biofilm formation (27). However, there are no reports of QS signaling molecules produced by *E. cloacae*.

*Shigella sonnei*, a Gram-negative, non-motile, and non-spore-forming bacterium belonging to the Enterobacteriaceae family, is one of the primary pathogens responsible for bacterial dysentery (28). The pathogenicity of *S. sonnei* is closely linked to its ability to form biofilms, which are complex bacterial communities that adhere to various solid surfaces by secreting extracellular polymeric substance, including extracellular polysaccharides (EPS), proteins, DNA, and lipids (29, 30). Our previous studies demonstrated that key pathogenic traits of *S. sonnei*, such as biofilm formation and virulence factor production, are regulated by its QS signaling molecule 4-hydroxybenzoic acid (4-HBA) (31). Given the clinical importance of *S. sonnei* and its prevalence in gut infections, we further explored whether gut commensals such as *E. cloacae* interact with *S. sonnei* via QS signaling, thereby modulating its pathogenic behavior.

In this study, we explore the interactions between *E. cloacae* and *S. sonnei*, revealing that indole-3-ethanol produced by YjgB in *E. cloacae* not only inhibits the biofilm formation and EPS synthesis but also disperses the biofilm biomass of *S. sonnei*. In-frame deletion of *yjgB* disrupted biofilm formation, motility, EPS production, and virulence in *E. cloacae*, whereas exogenous addition of indole-3-ethanol restored these phenotypes to wild-type strain levels. Our data suggest that indole-3-ethanol is a QS signal employed by bacteria and fungi to play an important role in microbial ecology, which plays a crucial role not only in the physiology of *E. cloacae* but also in antagonistic interactions with other enteric pathogens, thus having significant implications for the dynamic balance of the gut microbiota and disease control.

## RESULTS

### *E. cloacae* extract inhibits the pathogenic phenotype of *S. sonnei*

**Fig 1:**
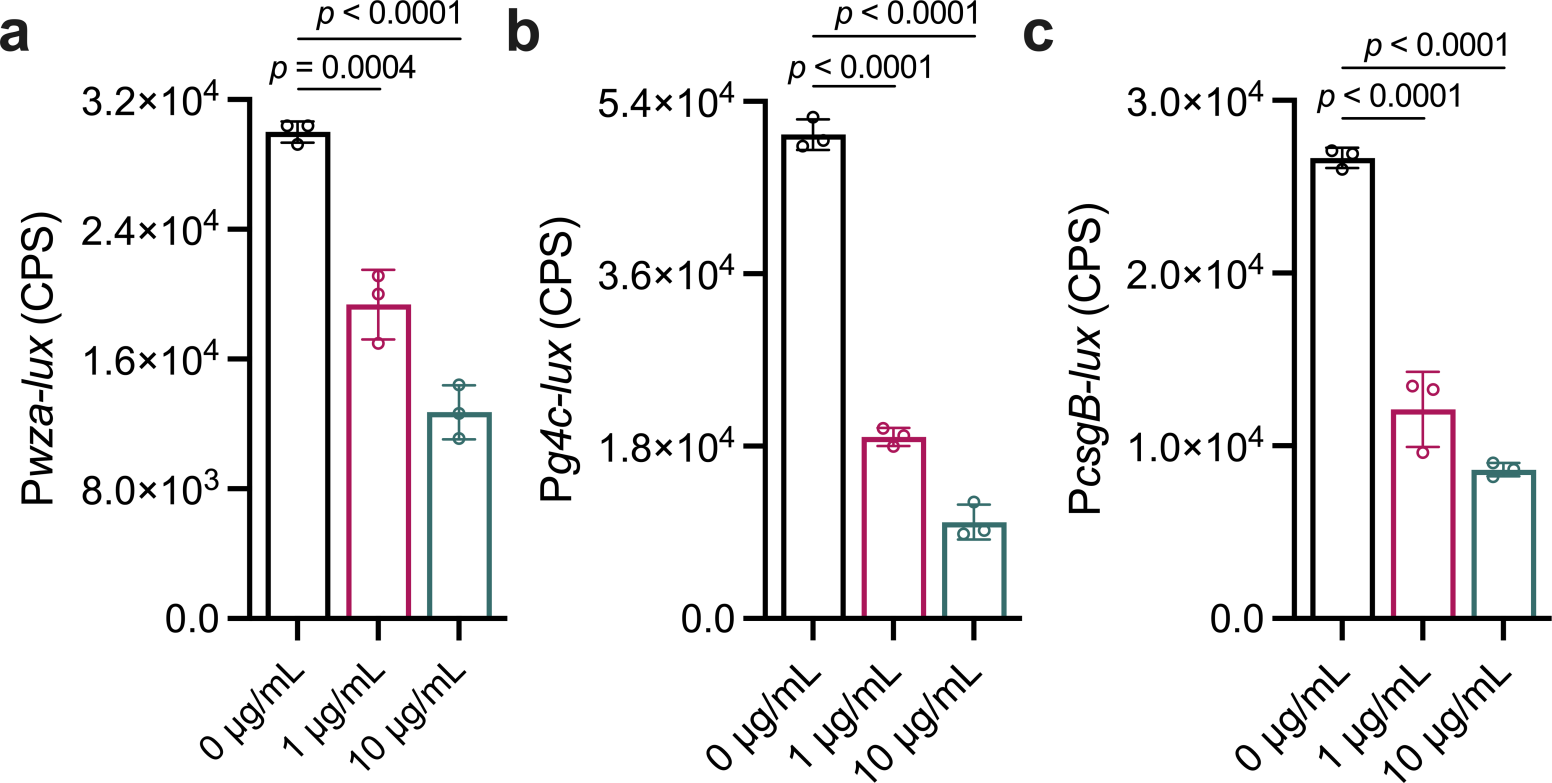
Effects of the ethyl acetate extract of *E. cloacae* on *S. sonnei*. The transcriptional expression of EPS biosynthesis- and biofilm-related genes, *wza* (a), *g4c* (b), and *csgB* (c), was analyzed in the absence and presence of 1 and 10 µg/mL ethyl acetate extract. The gene expression levels of *wza*, *g4c*, and *csgB* were evaluated by assessing light production (counts per second [cps]) by *wza-luxCDABE*, *g4c-luxCDABE*, and *csgB-luxCDABE* transcriptional fusions in the *S. sonnei* strains. The extract was dissolved in methanol, and the same volume of methanol used as the solvent for the compounds served as a control. The data are presented as mean ± SD and are representative of three independent experiments. The error bars indicate SDs. *P* values reflect one-way analysis of variance (ANOVA) tests used to determine the significance of the results. The source data are provided as a source data file.

Given the well-documented ability of certain gut microbes to inhibit the colonization of pathogens, we conducted a screening to assess their potential to suppress the growth or virulence of *S. sonnei*. The pathogenicity and virulence of *S. sonnei* rely on several key genes involved in EPS biosynthesis- and biofilm-related genes, including *wza*, which is essential for colanic acid synthesis (32); *csgB*, a key component in curli fiber formation (33); and the *g4c* operon, which is involved in O antigen capsule synthesis (34–36). These genes are related to bacterial adhesion and invasiveness, contributing to pathogen survival and infection efficiency. Through ethyl acetate extraction from the liquid culture of *E. cloacae*, we isolated low-molecular-weight compounds, which were subsequently concentrated via evaporation and redissolved in methanol for quantitative analysis. We found that the exogenous addition of the ethyl acetate extract of *E. cloacae* reduced the transcription of EPS biosynthesis- and biofilm-related genes, specifically *wza*, *g4c*, and *csgB*, but did not obviously affect the growth of *S. sonnei* (Fig. 1; Fig. S1). This finding suggests a potential mechanism whereby compounds produced by *E. cloacae* disrupt biofilm formation in *S. sonnei*, thereby affecting its ability to colonize and infect.

### The major active component of *E. cloacae* is indole-3-ethanol

**Fig 2:**
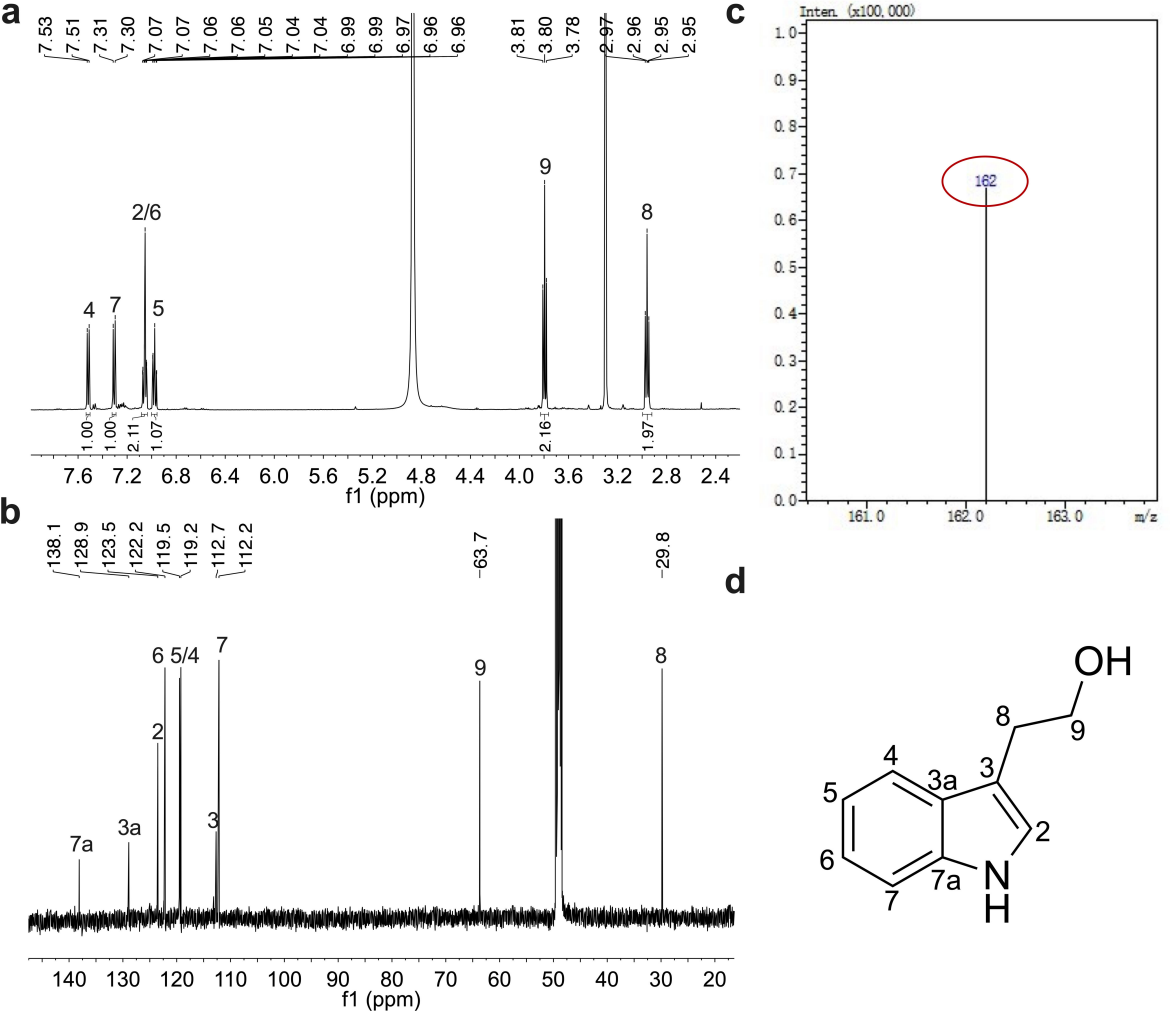
Structural characterization of indole-3-ethanol. (a) ^1^H NMR spectrum of indole-3-ethanol. (b) ^13^C NMR spectrum of indole-3-ethanol. (c) ESI-MS spectra of indole-3-ethanol. (d) Chemical structure of indole-3-ethanol.

To identify the active components of *E. cloacae* that inhibit biofilm formation and EPS biosynthesis in *S. sonnei*, we isolated and purified the active fractions from 100 L of *E. cloacae* subsp. *cloacae* ATCC 13047 culture supernatants via high-performance liquid chromatography (HPLC). Four active compounds were identified, and their chemical structures were confirmed by ^1^H and ^13^C nuclear magnetic resonance (NMR) spectroscopy. Among these peaks, approximately 23.6 mg of the purified compound of peak 1 was purified. Structural characterization revealed five protons in the aromatic region and four methylene protons in the ^1^H NMR spectrum (Fig. 2a). The ^13^C NMR data for the compound revealed the presence of eight aromatic carbons, one carbon attached to the hydroxyl group, and one methylene carbon (Fig. 2b). Electrospray ionization tandem mass spectrometry (ESI-MS) analysis of the active compound revealed a molecular ion [M + H]^+^ with an *m/z* ratio of 162.2 (Fig. 2c), matching the molecular formula C_10_H_11_NO. The results were consistent with the literature (37), indicating that the active compound was indole-3-ethanol (Fig. 2d). The results of the NMR spectroscopy analysis of the other three active compounds are shown in Fig. S2 and S3, and they were determined to be indole-3-pyruvic acid, indole-3-carbinol, and indole, respectively. We reevaluated the impact of these four compounds on the phenotypes of *S. sonnei* by adding 100 µM indole-3-ethanol, indole-3-pyruvic acid, indole-3-carbinol, and indole to the reporter systems and found that indole-3-ethanol had the strongest ability to reduce the transcription levels of *wza*, *g4c*, and *csgB* in *S. sonnei* (Fig. S4).

### Indole-3-ethanol inhibits the pathogenic phenotypes of *S. sonnei* in a dose-dependent manner

**Fig 3:**
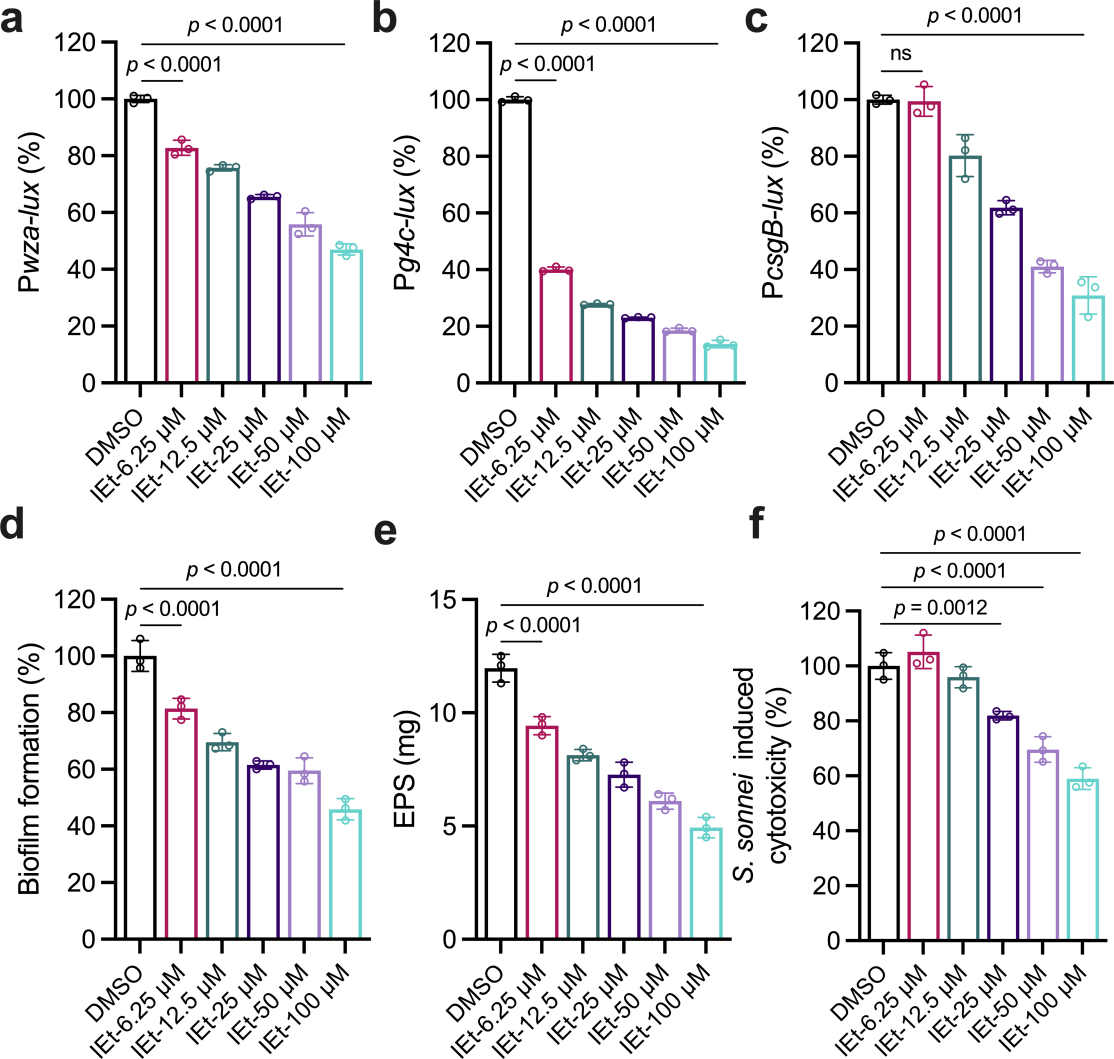
Effects of different concentrations of indole-3-ethanol on the phenotype of *S. sonnei*. The transcriptional expression of EPS biosynthesis- and biofilm-related genes, *wza* (**a**), *g4c* (**b**), and *csgB* (**c**), was analyzed in the absence and presence of 6.25, 12.5, 25, 50, and 100 µM indole-3-ethanol. The gene expression levels of *wza*, *g4c*, and *csgB* were evaluated by assessing light production (counts per second [cps]) by *wza-luxCDABE*, *g4c-luxCDABE*, and *csgB-luxCDABE* transcriptional fusions in the *S. sonnei* strains. Biofilm formation (**d**), EPS production (**e**), and cytotoxicity (f) in *S. sonnei* were analyzed. The extract was dissolved in dimethyl sulfoxide (DMSO), and the same volume of DMSO used as the solvent for the compounds served as a control. Indole-3-ethanol: IEt. The data are presented as the means ± SDs and are representative of three independent experiments. The error bars indicate SDs. *P* values reflect one-way ANOVA tests used to determine the significance of the results. The source data are provided as a source data file.

To assess the specificity and dose dependency of the impact of indole-3-ethanol on *S. sonnei*, various concentrations of this compound were tested for their effects on biofilm and EPS synthesis. Using reporter systems (P*wza-lux*, P*g4c-lux*, and P*csgB-lux*) linked to critical biofilm and virulence genes, dose-dependent inhibition was observed (Fig. 3a through c). Quantitative analyses confirmed the reduction in biofilm formation and EPS production with the addition of indole-3-ethanol in a dose-dependent manner (Fig. 3d and e). Furthermore, cytotoxicity assays revealed that indole-3-ethanol also reduced *S. sonnei*-induced cytotoxicity in the A549 cell line, suggesting that indole-3-ethanol not only interferes with the physiology but also directly attenuates the pathogenicity of *S. sonnei* (Fig. 3f). Additionally, we measured the effect of indole-3-ethanol on the growth rate of *S. sonnei* and found that the addition of exogenous indole-3-ethanol had little effect on its growth rate (Fig. S5). Moreover, the impact of indole-3-ethanol on the dispersion of *S. sonnei* biofilms was also investigated. Results indicated that indole-3-ethanol could promote the dispersion of *S. sonnei* biofilms in a dose-dependent manner, further strengthening its role in modulating biofilm-related behaviors of the pathogen (Fig. S6). In addition, we also tested commercially synthesized indole-3-ethanol, which displayed the same effect as indole-3-ethanol produced by *E. cloacae*.

### Indole-3-ethanol inhibits the QS signaling systems in *S. sonnei*

To elucidate the mechanism by which indole-3-ethanol inhibits the virulence phenotypes of *S. sonnei*, we continued to test whether indole-3-ethanol interferes with the signaling systems involved in the regulation of biofilm formation and EPS synthesis. Our recent study revealed that the enzyme encoded by the *trpE* gene is responsible for the synthesis of the QS molecule anthranilic acid in *Ralstonia solanacearum*, whereas the *ubiC* gene is involved in the synthesis of 4-HBA in *S. sonnei* (12, 31). RNA sequencing (RNA-Seq) and reverse transcription quantitative PCR (RT-qPCR) revealed that both genes were significantly downregulated following indole-3-ethanol treatment (Fig. S7a and Table S1). Furthermore, the reporter systems (P*trpE-lux* and P*ubiC-lux*) exhibited reduced transcriptional activity after treatment with 100 µM indole-3-ethanol. Liquid chromatography–mass spectrometry (LC-MS) analysis demonstrated a clear decrease in the levels of anthranilic acid and 4-HBA in the ethyl acetate extracts of *S. sonnei* under the same treatment conditions (Fid.S7b through e). Considering that both anthranilic acid and 4-HBA are derived from chorismate, it is hypothesized that indole-3-ethanol inhibits the biosynthesis of these critical QS molecules, thereby suppressing pathogenic phenotypes. Additionally, RNA-Seq and RT-qPCR analyses revealed that the addition of exogenous indole-3-ethanol inhibited the expression of the *tssG* gene, which is a component of the type VI secretion system, and several other genes associated with EPS biosynthesis, including *gmd*, *cpsG*, *manB*, *wza*, *fruB*, *rhaA*, and *rhaB*, were also downregulated (Fig. S7a and f).

### YjgB is responsible for indole-3-ethanol biosynthesis in *E. cloacae*

**Fig 4:**
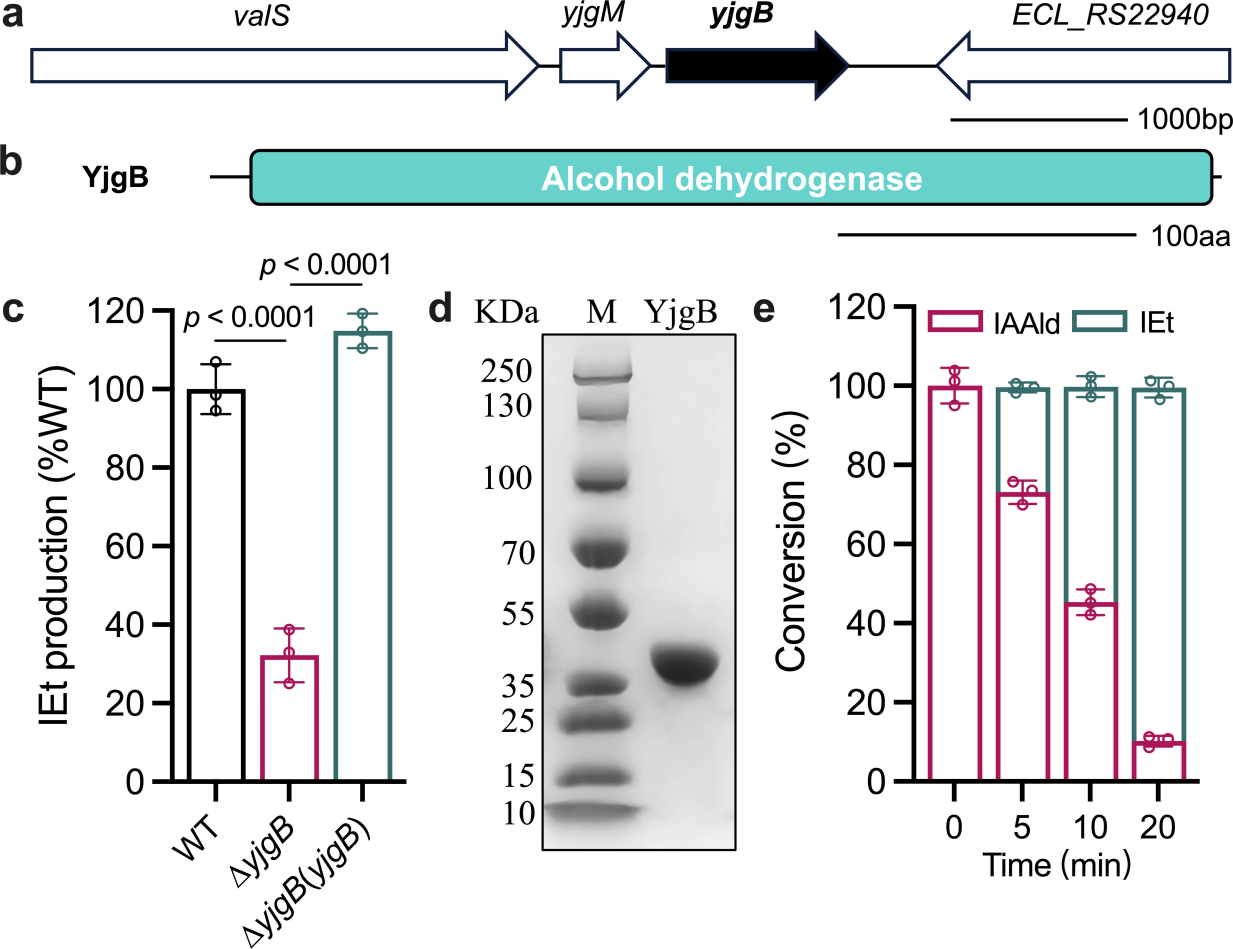
Analysis of the enzyme activity of YjgB in the synthesis of indole-3-ethanol. (**a**) Genomic organization of the *yjgB* region in *E. cloacae* subsp. *cloacae* ATCC 13047. (**b**) Domain architecture of the YjgB protein. (**c**) Detection of indole-3-ethanol production via the LC-MS assay. (**d**) SDS-PAGE analysis of the YjgB protein. (**e**) Analysis of the production of indole-3-ethanol by YjgB for indole-3-acetaldehyde transformation. Indole-3-ethanol: IEt. Indole-3-acetaldehyde: IAAld. The data are presented as the means ± SDs and are representative of three independent experiments. The error bars indicate SDs. *P* values reflect one-way ANOVA tests used to determine the significance of the results. The source data are provided as a source data file.

The alcohol dehydrogenase encoded by *yjgB* in *Escherichia coli* has been identified as a key enzyme involved in the biosynthesis of indole-3-ethanol (38). To identify the gene responsible for the biosynthesis of indole-3-ethanol, a search for *yjgB* homologs in the genome sequence of *E. cloacae* subsp. *cloacae* ATCC 13047 was conducted. The search was performed using the Basic Local Alignment Search Tool (BLAST) program from the National Center for Biotechnology Information, leading to the identification of *ECL_RS22935* (Fig. 4a and b). In-frame deletion of *yjgB* almost completely abolished indole-3-ethanol production (Fig. 4c) but did not affect the bacterial growth rate in Luria-Bertani (LB) medium (Fig. S8). To further confirm the enzymatic activity observed *in vitro*, the fusion protein YjgB, containing 339 amino acids with a calculated molecular weight of 36.2 kDa, was purified via affinity chromatography (Fig. 4d). *In vitro* enzymatic assays demonstrated that YjgB directly catalyzed the conversion of indole-3-acetaldehyde to indole-3-ethanol (Fig. 4e; Fig. S9).

### Deletion of *yjgB* impairs biological functions in *E. cloacae*

**Fig 5:**
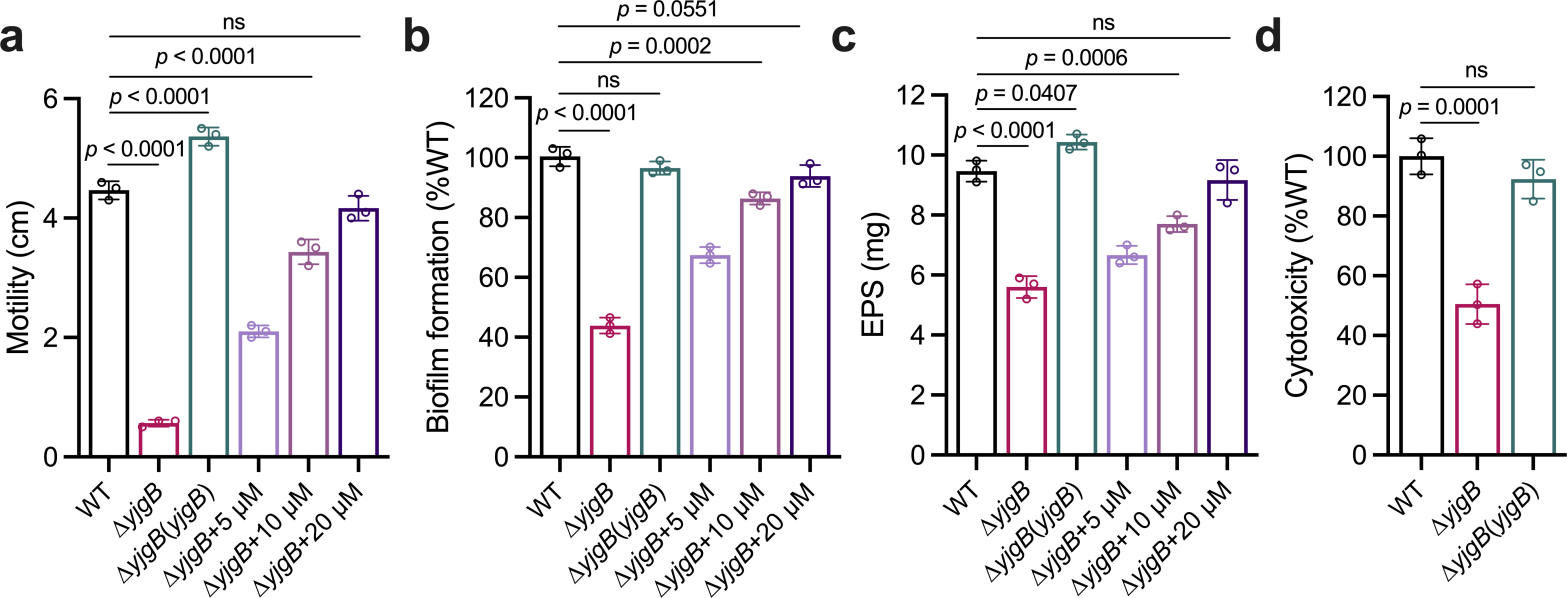
Effect of *yjgB* and indole-3-ethanol on the virulence-regulated phenotypes of *E. cloacae*. Motility (**a**), biofilm formation (**b**), and EPS production (**c**) in *E. cloacae* wild-type, *yjgB* deletion mutant, and *yjgB* complemented strains and the *yjgB* deletion mutant in response to the addition of different concentrations of indole-3-ethanol were analyzed. (**d**) The cell cytotoxicity of the *E. cloacae* wild-type, *yjgB* deletion mutant, and *yjgB* complemented strains was evaluated via a lactate dehydrogenase assay. Indole-3-ethanol: IEt. The data are presented as the means ± SDs and are representative of three independent experiments. The error bars indicate SDs. *P* values reflect one-way ANOVA tests used to determine the significance of the results. The source data are provided as a source data file.

It was determined that indole-3-ethanol inhibits the biofilm formation and EPS synthesis of *S. sonnei* through interspecies interference, and we further investigated whether this compound plays a role in regulating the biological functions of *E. cloacae*. Since in-frame deletion of *yjgB* nearly completely abolished the production of indole-3-ethanol, we subsequently tested biofilm formation, motility, and EPS production in the *yjgB* deletion mutant. The results indicated that the deletion of *yjgB* significantly impaired these phenotypes, particularly motility (Fig. 5) but had little effect on the growth of bacterial cells (Fig.S8). Interestingly, the overexpression of *yjgB* or the exogenous addition of 20 µM indole-3-ethanol restored the phenotypes of the *yjgB* deletion mutant to those of the wild-type strain. Furthermore, compared with that of the wild-type *E. cloacae* strain, the cytotoxicity was decreased by 49% when A549 cells were incubated with the *yjgB* deletion mutant at 8 h postinoculation (Fig. 5d). These results suggest that indole-3-ethanol plays a crucial role in regulating the biological functions and pathogenicity of *E. cloacae* through intraspecies signaling.

### The production of indole-3-ethanol is cell density dependent and autoinduced

**Fig 6:**
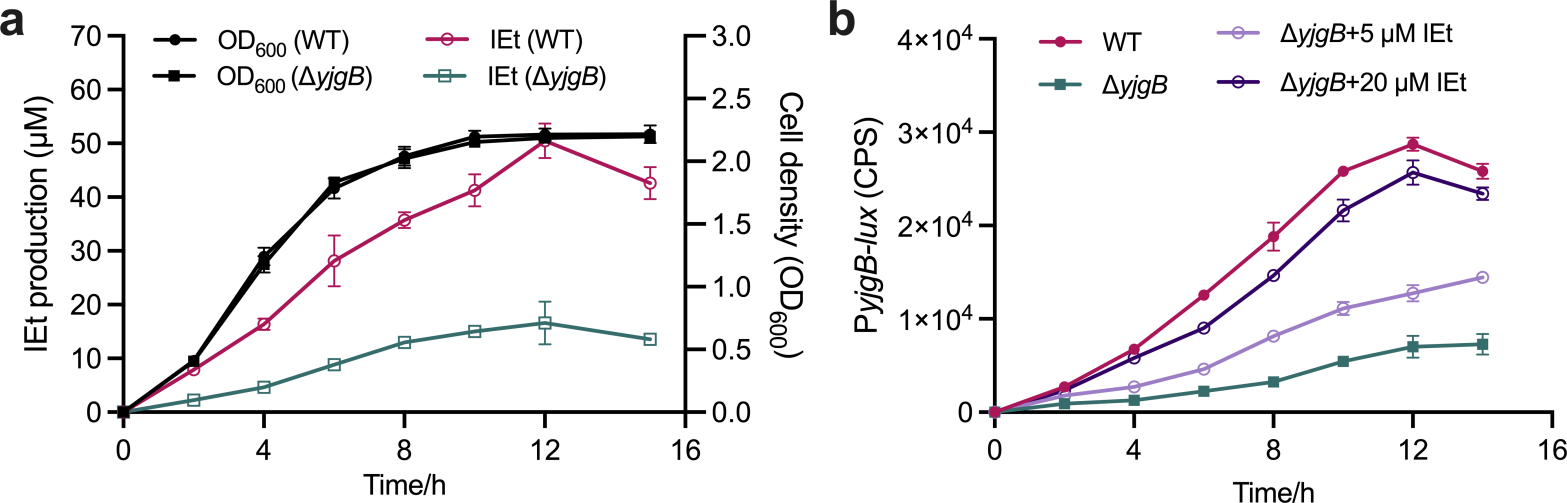
Analysis of indole-3-ethanol production and *yjgB* transcriptional expression. (**a**) Time-course analysis of indole-3-ethanol accumulation and cell growth in the *E. cloacae* wild-type strain and the *yjgB* deletion mutant in liquid medium. (**b**) Gene expression levels of *yjgB* in the *E. cloacae* wild-type strain, the *yjgB* deletion mutant, and the *yjgB* deletion mutant supplemented with indole-3-ethanol. The gene expression levels of *yjgB* were evaluated by assessing light production (counts per second [cps]) by *yjgB-luxCDABE* transcriptional fusions in the *E. cloacae* strains. Indole-3-ethanol: IEt. The data are presented as the means ± SDs and are representative of three independent experiments. The error bars indicate the SDs. The source data are provided as a source data file.

To explore whether the biosynthesis of indole-3-ethanol is correlated with cell density, we first analyzed the time course of indole-3-ethanol production by measuring its concentration at different growth stages. During the initial growth stage, the production of indole-3-ethanol resulted in relatively low yields; however, at higher cell densities, there was a dramatic increase in indole-3-ethanol production after 4 h, reaching a peak of 50.5 µM at 12 h, after which the concentration of indole-3-ethanol began to decrease (Fig. 6a; Fig. S10).

We subsequently examined the transcriptional profile of *yjgB*, which is essential for indole-3-ethanol production. A 495 bp DNA sequence encompassing the *yjgB* promoter region was genetically engineered to be transcriptionally coupled with the *luxCDABE* operon and subsequently transformed into both the wild-type and *yjgB* deletion mutant. The transcription level of *yjgB* in the wild-type strain gradually increased and peaked at 12 h post-inoculation, followed by a decrease (Fig. 6b), which was strongly correlated with the accumulation profile of indole-3-ethanol.

To determine whether the transcription of *yjgB* is autoregulated by indole-3-ethanol, we compared the transcriptional profiles of *yjgB* in the wild-type strain, *yjgB* deletion mutant, and *yjgB* deletion mutant supplemented with indole-3-ethanol. The results indicated that the promoter activity in the mutant strain was significantly lower than that in the wild-type strain. Additionally, the addition of exogenous indole-3-ethanol at a final concentration of 20 µM restored the promoter activity of *yjgB* in the *yjgB* deletion mutant to nearly the wild-type level, suggesting that the production of indole-3-ethanol may be autoregulated at the transcriptional level (Fig. 6b).

### Indole-3-ethanol controls the expression levels of a wide range of genes

To further investigate the regulatory role of *yjgB* in controlling bacterial biological functions, we analyzed and compared the transcriptomes of the wild-type strain and the *yjgB* deletion mutant using RNA-Seq. Differential gene expression analysis revealed that 26 genes were upregulated, whereas 156 genes were downregulated in the *yjgB* mutant compared with the wild-type strain (Fig. S11a). These differentially expressed genes were associated with various biological functions, including motility and cell attachment, stress tolerance, virulence, regulation, membrane components, transport, metabolism, multidrug resistance, and signal transduction (Fig. S11b).

Interestingly, among these differentially expressed genes, several flagella-related genes (such as *fliI*, *fliK*, *fliP*, *flhB*, *flgE*, *flgF*, *flgH*, *flgI*, *motA*, and *motB*) and a methyl-accepting chemotaxis protein (*mcp*) were downregulated in the *yjgB* deletion mutant (Fig. 5a). Additionally, some genes associated with bacterial secretion systems (such as *vasG*, *impL*, and *gspD*) were downregulated. Moreover, the transcription levels of genes related to carbohydrate metabolism (such as *ECL_RS11710*, *ECL_RS08815*, *ECL_RS25290*, *ECL_RS22560*, and *ECL_RS00045*) were downregulated, which may affect the synthesis of EPS. These findings suggest that *yjgB* may play a critical regulatory role in bacterial motility, virulence, and metabolic processes (Fig. S11 and Table S2).

## DISCUSSION

QS is a sophisticated cell-cell communication mechanism in both bacteria and fungi that coordinates various complex behaviors by producing and sensing diffusible signaling molecules (39, 40). A variety of QS signals, such as AHLs, DSFs, PQS, AI-2, and anthranilic acid, have been identified. Among these, indole and its derivatives are considered key QS signaling molecules in various bacterial species, regulating microbial behavior and facilitating both interspecies and intraspecies interactions (41–43). Studies have shown that indole and 7-hydroxyindole regulate the gene expression and virulence of *Pseudomonas aeruginosa* by affecting the *mexGHI-opmD* efflux pump (44). The degradation of indole-3-acetic acid (IAA), a plant hormone, involves specific enzymes such as LacA and LacB, which are encoded by the *iac* gene cluster, increasing chemotaxis and survival in *Pseudomonas putida* 1290 (45). Other indole derivatives, including indole-3-acetonitrile, reduce biofilm formation in *E. coli* O157:H7 and attenuate the virulence of *P. aeruginosa* (46). Our previous study identified a novel indole biosynthesis enzyme, AbiS, and demonstrated the critical role of indole signaling in regulating the physiology and virulence of *A. baumannii* (13). We also discovered several other indole derivatives, including IAA, 2-(2,2-di[1H-indol-3-yl]ethyl)aniline, and methyl 2-(1H-indol-3-yl)acetate, which significantly inhibited the motility and cytotoxicity of *P. aeruginosa* (13). In this study, we elucidated the significant biological functions of indole-3-ethanol in *E. cloacae* and revealed its inhibitory effects on the biofilm formation and EPS production in *S. sonnei*, as well as the dispersion activity on the biofilm biomass of *S. sonnei*. The transcriptomic analysis further revealed that, compared with the *E. cloacae* wild-type strain, the deletion of the indole-3-ethanol synthase-encoding gene *yjgB* significantly altered the expression of approximately 200 genes. Overall, our findings, along with those of previous studies, support the notion that indoles and their derivatives, collectively termed “indole-family QS signals,” are likely widespread among bacteria and regulate a variety of critical physiological processes. This research not only deepens our understanding of indole-3-ethanol-mediated signaling but also highlights the potential universality of indole-family QS signals in bacterial communications.

Previous studies have identified indole-3-ethanol as a QS signaling molecule with a pivotal role in regulating fungal physiology (18–23). Indole-3-ethanol has been shown to influence critical processes such as hyphal development, biofilm formation, and the yeast-to-hyphae transition, which are key determinants of its pathogenicity and adaptability in *C. albicans* (20). Indole-3-ethanol extends its regulatory influence beyond *C. albicans* to other fungi, such as *S. cerevisiae* and *S. spartinae*, by impacting morphogenesis, virulence, and resistance to antifungal agents, indicating its broader significance in fungal biology (15, 19, 23, 47). Simultaneously, it mediates interspecies communication, facilitating interactions and coordination among diverse fungal species (23). Our study demonstrated that indole-3-ethanol is involved not only in intraspecies bacterial signaling but also in interspecies communication among bacteria. These findings suggest that, in natural environments, indole-3-ethanol acts as a broad-spectrum signaling molecule, playing roles in both intraspecies and interspecies communication within fungi and bacteria. Given the frequent coexistence of bacteria and fungi in natural microbial ecosystems, it is likely that indole-3-ethanol also mediates cross-kingdom communication between bacteria and fungi. On the basis of our findings and those of previous studies, indole-3-ethanol serves as a crucial interspecies signal, and its role in cross-kingdom communication and function in microbial ecology warrants further investigation.

In this study, we discovered that the alcohol dehydrogenase YjgB catalyzes the conversion of indole-3-acetaldehyde to indole-3-ethanol. Both *in vivo* and *in vitro* experiments confirmed that YjgB has enzymatic activity for indole-3-ethanol synthesis in *E. cloacae*. Amino acid sequence comparisons revealed a high degree of homology between YjgB in *E. cloacae* and the alcohol dehydrogenase YjgB identified in *E. coli*, suggesting that this enzymatic function is evolutionarily conserved across species. BLAST analysis further revealed that homologs of YjgB are highly conserved across various *Enterobacter* genotypes, such as *E. asburiae*, *E. huaxiensis*, *E. mori*, and *E. sichuanensis*, with high identities of more than 98%. YjgB also exhibits widespread conservation in several other bacterial species, including *Leclercia pneumoniae*, *Klebsiella pneumoniae*, *Lelliottia nimipressuralis*, and *Citrobacter portucalensis* (Table S3), indicating its broader ecological significance. Similarly, in the fungus *S. cerevisiae*, indole-3-ethanol is synthesized by the alcohol dehydrogenase ADH1, which catalyzes the conversion of indole-3-acetaldehyde to indole-3-ethanol (48). BLAST analysis further revealed that homologs of this enzyme are widely distributed across various fungal species, such as *Botrytis cinerea*, *Nakaseomyces glabratus*, *Armillaria ostoyae*, and *Yarrowia lipolytica*, with sequence identities exceeding 82% (Table S4), suggesting that indole-3-ethanol may also be widespread in fungi. Overall, these findings indicate that indole-3-ethanol functions as a highly conserved QS signal widely present in bacterial and fungal taxa.

In bacteria, indole may function through various receptors, including the two-component systems BaeSR and CpxAR in *E. coli* (49), EmhR in *Pseudomonas fluorescens* (50), the sensor histidine kinase QseC in *Lysobacter enzymogenes* (51), DksA in *Vibrio cholera*e (52), pyruvate kinase in *Stigmatella aurantiaca* (53), and IsrR in enterohemorrhagic *E. coli* (54). These receptors regulate quorum sensing, virulence, and physiological adaptation mediated by indoles across different bacterial species. In our recent study, we identified a novel indole response regulator, AbiR, in *A. baumannii*, which contains a CheY-homologous REC domain and an HTH DNA-binding domain (14). In addition to indole, its derivatives, such as IAA, indole-3-acetonitrile, and indole-3-carbinol, function as signaling molecules that regulate processes such as gene expression, biofilm formation, and pathogenicity in bacteria (45, 46, 55, 56). However, the specific receptors for these compounds in bacterial systems remain largely unidentified, making fully elucidating their signaling pathways and mechanisms challenging. Since indole-3-ethanol is also an indole derivative, we employed the BLAST program to search for homologs of known indole receptors in *E. cloacae*, aiming to identify potential receptors for indole-3-ethanol. Our analysis revealed seven potential homologs with sequence identities ranging from 33.65% to 83.91% with established indole receptors (Table S5). These findings highlight the complexity of the signaling pathways of indole-family QS signals in bacteria.

In conclusion, our study highlights the presence of the indole-3-ethanol signaling system in various bacterial species, but the detailed signaling pathways and regulatory mechanisms involved remain to be elucidated. Our primary focus in this study was on the role of indole-3-ethanol in mediating intraspecies signaling in *E. cloacae* and interspecies communication with *S. sonnei*. These findings provide a foundation for further research into the regulatory mechanisms by which indole-family QS signals influence pathogenicity and microbial interactions.

## MATERIALS AND METHODS

### Bacterial strains and growth conditions

The bacterial strains and plasmids used in this study are listed in Table 1. All plasmids and strains used in this study were sequenced. *E. cloacae* subsp. *cloacae* ATCC 13047 and *S. sonnei* were cultured in LB medium (10 g/L tryptone, 5 g/L yeast extract, and 10 g/L NaCl; pH 7.4) or LB agar medium (LB medium containing 15 g/L agar) and grown at 37°C. The following antibiotics were added as necessary: kanamycin (100 µg/mL) and ampicillin (100 µg/mL). Bacterial growth was assessed by measuring the optical density at a wavelength of 600 nm.

**TABLE 1:** Bacterial strains and plasmids used in this study

Strain or plasmid	Phenotype and/or characteristic(s)^*a*^	Source or reference
*E. cloacae* strains		
subsp. *cloacae* ATCC 13047	Wild-type strain of *E. cloacae*	Laboratory collection
Δ*yjgB*	Indole-3-ethanol-minus mutant derived from ATCC 13047 with *yjgB* being deleted	This study
Δ*yjgB*(*yjgB*)	Mutant *yjgB* harboring the expression construct *pBBR-yjgB*	This study
*E. cloacae* (P*yjgB-lux*)	*E. cloacae* harboring the reporter construct P*yjgB-lux*	This study
*S. sonnei* strains		
CMCC51592	Wild-type strain of *S. sonnei*	Laboratory collection
*S. sonnei* (P*wza-lux*)	*S. sonnei* harboring the reporter construct P*wza-lux*	This study
*S. sonnei* (P*g4c-lux*)	*S. sonnei* harboring the reporter construct P*g4c-lux*	This study
*S. sonnei* (P*csgB-lux*)	*S. sonnei* harboring the reporter construct P*csgB-lux*	This study
*S. sonnei* (P*trpE-lux*)	*S. sonnei* harboring the reporter construct P*trpE-lux*	Laboratory collection
*S. sonnei* (P*ubiC-lux*)	*S. sonnei* harboring the reporter construct P*ubiC-lux*	Laboratory collection
*E. coli* strains		
DH5α	*supE44 lacU169(80lacZ M15) hsdR17 recA1 endA1 gyrA96 thi-1 relA1 pir*	Laboratory collection
BL21(DE3)	*F-ompT hsdS (rB-mB-) dcm+ Tet^r^ gal (DE3) endA*	Laboratory collection
Plasmids		
pBBR1-MCS2	Broad-host-range cloning vector, Kan^r^	Laboratory collection
pET28(a)+	Expression vector, Kan^r^	Laboratory collection
pKD4	Template for amplifying the *kan* gene	Laboratory collection
pKD46-tet	λ Red recombinase expression, Amp^r^, Tet^r^	Laboratory collection
pCP20	FLP^*b*^ recombinase expression, Amp^r^	Laboratory collection
pMS402	Expression reporter plasmid carrying the promoterless *LuxCDABE*	Laboratory collection
pET-*yjgB*	pET28 containing *yjgB*	This study
P*wza-lux*	pMS402 containing the promoter of *wza*	This study
P*g4c-lux*	pMS402 containing the promoter of *g4c*	This study
P*csgB-lux*	pMS402 containing the promoter of *csgB*	This study
P*trpE-lux*	pMS402 containing the promoter of *trpE*	Laboratory collection
P*ubiC-lux*	pMS402 containing the promoter of *ubiC*	Laboratory collection

^*a*^ Kan^r^, Amp^r^, and Tet^r^ indicate resistance to kanamycin, ampicillin, and tetracycline, respectively.

^*b*^ FLP, Flippase recombination enzyme.

### Purification and structural analysis

*E. cloacae* ATCC 13,047 cells were cultured in LB medium for 12 h and then centrifuged at 4,000 rpm before the supernatant was extracted with an equal volume of ethyl acetate. The ethyl acetate phase was concentrated by evaporation to obtain the extract, which was subsequently dissolved in methanol. The extract was analyzed by HPLC using a C18 reverse-phase column (Atlantis T3 column, 5 µm, 4.6 mm × 250 mm) with a gradient elution of acetonitrile-water (from 5:95 to 100:0 [vol/vol]) at a flow rate of 1 mL/min. The active fractions were further purified by HPLC on a semipreparative C18 reverse-phase column and eluted with a gradient of acetonitrile:water (from a volume ratio of 20:80 to 60:40) at a flow rate of 3 mL/min. Peaks were detected using a UV detector at 210 nm and collected and assayed.

The ^1^H and ^13^C NMR spectra in CD_3_OD solution were obtained using a Bruker AV-500 (Bruker Instrument, Inc., Zurich, Switzerland) spectrometer operating at 500 MHz for ^1^H or 125 MHz for ^13^C. Ultrahigh-performance liquid chromatography (UHPLC)-ESI-MS/MS was performed in an LC-30A UHPLC system (Shimadzu Corporation, Kyoto, Japan) with a Waters C18 column (1.8 µm, 150 × 2.1 mm) and a Shimadzu 8060 QQQ-MS mass spectrometer with an ESI source interface (Shimadzu Corporation, Kyoto, Japan).

### Construction of the *E. cloacae* mutant and complemented strains

All primer sequences are summarized in Table 2. The *yjgB* deletion mutant was generated via the λ Red recombinase system, which is supported by the pKD46-Tet plasmid, which encodes three essential proteins (Exo, Beta, and Gam) for homologous recombination. The kanamycin-resistant pKD4 plasmid served as the template for designing primers to encompass the 39 bp homologous arm sequences. The pCP20 plasmid was utilized to excise the kanamycin resistance. Moreover, the target gene was integrated into the plasmid using pBBR1-MCS2 to obtain the complemented strains. The resulting constructs were introduced into *E. cloacae* deletion mutants via electroporation.

**TABLE 2:** PCR primers used in this study^*a*^

Primer	Sequence (5′−3′)
For deletion	
*yjgB*-KO-F	CTCTACACTCTCAGTTCACACCATAACAGGGGAAACACGGTGTAGGCTGGAGCTGCTTC
*yjgB*-KO-R	TGGGGAACCCTCAGCCCTGTGGGAGAGGGGTGTGTCACAATGGGAATTAGCCATGGTCC
For *in trans* expression	
*yjgB*-pBBR-F	CCCAAGCTTGATGTCGAAGATAAAAAGCTACGC
*yjgB*-pBBR-R	GGGGATCCTTAAAAATCTGCTTTCAACACCA
For reporter	
P*yjgB-lux*-F	CCGCTCGAGGTCATCGCCAAAGAGCGTG
P*yjgB-lux*-R	CGGGATCCGATCCGACGCATCGTCGTC
P*wza-lux*-F	CCGCTCGAGATCGGCAATCCATTCCATAGT
P*wza-lux*-R	CGGGATCCTGTTTATTTATCACTTTGGCAGAGTAAT
P*g4c-lux*-F	CCGCTCGAGTATGACGCTGCTTGTTTAAAGC
P*g4c-lux*-R	CGGGATCCCGCATGGACAATACGTACG
P*csgB-lux*-F	CCGCTCGAGGATAACAGCGTATTTACGTGG
P*csgB*-lux-R	CGGGATCCGTTGTCACCCTGGACCTG
For recombinant protein expression	
YjgB-P28-F	CAGCAAATGGGTCGCGGATCCATGTCGAAGATAAAAAGCTACGCC
YjgB-P28-R	TTGTCGACGGAGCTCGAATTCTTAAAAATCTGCTTTCAACACCACA
For deletion confirmation	
Out-*yjgB*-F	TGCGAAGTCAGGATGCTGAA
Out-*yjgB*-R	TGTGGGAGAGGGGTGTGTCA
in-*yjgB*-F	TATCCACTGGTCGCCGGAC
in-*yjgB*-R	AAGACGGTAATGCCGCCG
For RT-qPCR analysis *E. cloacae*	
*fliI*-F	CTATCCGCCGTCTGTCTT
*fliI*-R	CTCCGTCAGCACCGTATA
*fliK*-F	TAACCATCCGCCAGAAAC
*fliK*-R	ATGCTCGCTGACGATATG
*fliP*-F	CCTTCCAGATTGGCTTCA
*fliP*-R	CAGCACAAACAGCATCAG
*flhB*-F	CAGATGGTGTCGGAGATG
*flhB*-R	GCAGCGGTTCATACATCA
*flgE*-F	AACAACATCGCCAACTCT
*flgE*-R	AGAAGCACCATTCATCAGT
*flgF*-F	GTTTCACGACACCACCAT
*flgF*-R	TCCTGATCCACTTCAATAGC
*flgH*-F	GAGAAGCAGATCGCCATC
*flgH*-R	TCGTTGATGTAGCCGTTG
*flgI*-F	GCGAAGGTGATTGTGAAC
*flgI*-R	GCTGGCTGATGATGTTGT
*motA*-F	GACTACTTCCGTCTGATGAT
*motA*-R	GCACTTCCAGCTCTTCTT
*motB*-F	ATTGCCGATTACTTCCGTAT
*motB*-R	GCCGCTTGAGAATGTCAT
*mcp*-F	TCTCCACATTGACTCTTCTG
*mcp*-R	CTGTTCGCCAATCTCCTG
*vasG*-F	AAGAGGTACAGCAGGAAGA
*vasG*-R	CGTTAGCGGTGACATCAA
*impL*-F	GCAATACGGCAGTCTCAT
*impL*-R	GGCTCCACTTGTCATTGA
*evgS*-F	TCGCTGTTGCTGCTTATC
*evgS*-R	GACACCGTTCCTTCAATCA
*gspD*-F	TGCGTGATGATGACAACTA
*gspD*-R	GCGTTATCGGTGTTCTCA
*ECL_02390-*F	CCGTACTACTACAACCACAA
*ECL_02390-*R	CGTCAATACTCAACACAACT
*ECL_01788-*F	CCTTAATGCCGTTGTATTCG
*ECL_01788-*R	AATCCCTTTCAGCGTTTCA
*ECL_05072-*F	GCAACATCGGTATTCAACT
*ECL_05072-*R	GCTCCTTAGTCAGTCGTT
*ECL_05073-*F	GGCACCCGCAATATGAAA
*ECL_05073-*R	TACGCTGGCAGTACATCT
*ECL_04525-*F	GCTGAAGGTACTGGAACTG
*ECL_04525-*R	TATACGGATGGCTGGAGATA
*ECL_00009-*F	GAGAGCAGGCAATCAGAA
*ECL_00009-*R	GACAGGAACAGGCAGATAA
*ECL_RS18095*-F	ATTGAGCAGCACCACACT
*ECL_RS18095*-R	GCCAGCATAACGATCACAT
*ECL_RS01435*-F	AGAGAACTGACGAGAAGAAC
*ECL_RS01435*-R	TTGCGAAGACACGGATAG
*sdhD*-F	CGCTGTTCTCCATTCTTATTC
*sdhD-*R	CACACCACAACGAATCCA
*ECL_RS20500*-F	GGTTCTTCCTCTACTACTTCA
*ECL_RS20500*-R	CCACCTCTTCCAGATTGTC
*ECL_RS18085*-F	GCCTGATTGCCTTAATTGG
*ECL_RS18085*-R	AACATACCGCCTAATGGATT
*rpoB*-F	AACTCTGCGTGCTGATAAG
*rpoB*-R	GGATACATCTCGTCTTCGTTA
*S. sonnei*	
*sucD-*F	TCTGGAAGCTATTGATGCAG
*sucD*-R	CGATCATACGAACGCCGG
*sdhD*-F	CCACCAGTGGCGAACTGA
*sdhD*-R	GGTCAACACCTGCCACAT
*sdhD*-F	CCACCAGTGGCGAACTGA
*sdhD*-R	GGTCAACACCTGCCACAT
*argT*-F	TTCATCTTGTAACGCAGCA
*argT*-R	TAACGAGACCTGGCGTAG
*zntR*-F	CTATCCGTGCTTCGACTT
*zntR*-R	ATCCGCATCGATCCTGAA
*msrB*-F	CTGCATAACAAGCGTGAC
*msrB*-R	TTCACTCACCGGTTCGTA
*kdpB*-F	CACTGAGAATGGCGGAGT
*kdpB*-R	CTTCGCCATTATTCCGGC
*citE*-F	GAGGTTGTGCAACAGATCAA
*citE*-R	CAGGCGTTCGATACCGTC
*citX*-F	GATGACTATGTTCAAGCTC
*citX*-R	TGGCAAATTCAGGAGCAG
*cpsG*-F	CACATCACCGCGAGATTC
*cpsG*-R	CGGTGGATCGCACCGATG
*eutG*-F	GCCAGACCGTGTGGAATA
*eutG*-R	ATGTATGGCGGGAATGGC
*fruB*-F	ATGGTATTGACCAGCATG
*fruB*-R	TGCTGACCAGCGATGATG
*gmd*-F	TCGAGGTCATTAGCCACC
*gmd*-R	GTTACTTCCGTCCGGCTG
*hyaB*-F	ACGATCAGAATGTGATCA
*hyaB*-R	CGCAGATACGTTCAACGA
*leuA*-F	GATATTGTTCAGTACGTGC
*leuA*-R	TTGTCGCCAACTACAACG
*pta*-F	AGTCGTTCTGGTTGAAGG
*pta*-R	TGAGACATAACGAAGACG
*ubic*-F	TATGTGCCGATGGTGAAC
*ubic-*R	CGATGATGTGAACAGATAGC
*rhaA*-F	TATTGCGTGTACCAATGA
*rhaA*-R	AGTGAGATTGTTCGTCAC
*rhaB*-F	TCATGCAACACATCGGCATA
*rhaB*-R	TCCTGAGGCGATGTGCAG
*trpE*-F	ATTGCAGAATCTGTTGAAT
*trpE*-R	GCCACAAGGTCATAAGAG
*tssG*-F	TTCTTCCGGTGTGCCGAG
*tssG*-R	TGTCCTTCATCCTGCGCG
*wza*-F	CATCGGACATATCCAGCT
*wza*-R	GAAGGTATCGACATGACC
*hisG*-F	GTGATGACTCACGCGAAT
*hisG*-R	CACGCACGCGCAGAATAT

^*a*^ Restriction enzyme sites are underlined. F, forward; R, reverse.

### Construction of reporter strains and measurement of activity

The plasmid pMS402, which carries a promoterless *luxCDABE* reporter gene cluster, was utilized to construct promoter-*luxCDABE* reporter fusions as previously described (57, 58). The target promoter was amplified via PCR and subsequently cloned upstream of the *lux* gene at the BamHI-XhoI site on the pMS402 plasmid. The resulting plasmid was introduced into *E. cloacae* via electroporation. Promoter activity was measured using the Infinite E PLEX Microchor Board detector and is expressed as luminescence (counts per second).

### Phenotypic analysis

*E. cloacae* biofilms were quantified in 96-well plates. A single colony of each strain was inoculated and cultured overnight in LB medium at 37°C with agitation. The bacterial cells were diluted to an optical density (OD_600_) of 0.1 and then added to 96-well plates. After incubation at 37°C for 24 h, the medium was removed, and the wells were gently washed three times with phosphate-buffered saline (PBS). This was followed by staining with 0.2% crystal violet for 30 min. After several washes with ddH_2_O, 150 µL of 95% ethanol was added. The formation of biofilms was quantified by measuring the optical density of the solution at 590 nm (OD_590_).

A similar approach was used to quantify *S. sonnei* biofilms but with some modifications. The bacterial cells were diluted to an OD_600_ of 0.05, and different concentrations of extract (0, 1, and 10 µg/mL) or indole-3-ethanol (0, 6.25, 12.5, 25, 50, and 100 µM) were added to the wells along with the bacterial suspension. The plates were incubated at 37°C for 36 h. The subsequent processing was the same as before.

To assess the impact of indole-3-ethanol on *S. sonnei* biofilm dispersion, the formed biofilms after static culture at 37°C for 36 h were treated with different concentrations of indole-3-ethanol (0, 6.25, 12.5, 25, 50, and 100 µM) for 4 h at 37°C. After treatment, the medium was discarded, and the wells were washed three times with PBS. The subsequent processing was the same as before.

Motility was assessed on semisolid agar. The bacteria were allowed to migrate in modified M9 medium (59, 60). The medium comprised 0.26% agar, M9 minimal salts (BD), 22 mM (0.4%) glucose, 3 mM sodium succinate, 0.25% glycerol, and 20 µg/mL (0.002%) of each amino acid: L-histidine (0.13 mM), M-methionine (0.13 mM), D-threonine (0.17 mM), and L-leucine (0.15 mM). The plates were incubated at 37°C for 24 h before the diameter of the movement trace was measured.

To quantify the production of EPS, bacteria were inoculated into LB medium. A 30 mL culture aliquot (OD_600_ = 3.0) was collected and centrifuged at 12,000 rpm for 30 min to remove the precipitate. The collected supernatants were mixed with four volumes of absolute ethanol, and the mixture was incubated at 4°C overnight. The mixture was subsequently centrifuged at 4°C and 12,000 rpm for 30 min. The supernatant was discarded, and the precipitate was isolated and air-dried at 55°C. The mass of the precipitate was determined, and the experiment was repeated three times.

Cell cytotoxicity was assessed by measuring the release of lactate dehydrogenase (LDH) from A549 cells. A549 cells were cultured at a density of 1 × 10^5^ cells/well in 96-well tissue culture plates with Dulbecco’s modified Eagle’s medium (DMEM) supplemented with 10% fetal bovine serum (FBS). Bacterial cells were grown in LB medium at 37°C overnight, centrifuged, and resuspended in DMEM containing 1% FBS. A549 cells were infected with bacteria at a concentration of 10^9^ CFU/mL for 8 h. The release of LDH was determined via a CytoTox 96 assay kit (Promega, Wisconsin, USA). The cytotoxicity assay results were quantified by measuring the absorbance at 490 nm and calculating the cytotoxicity relative to that of the uninfected control group.

### Protein expression and purification

Enzyme expression and purification were carried out via methods described in previous studies with modifications (61). Using the primers listed in Table 2, we amplified the coding region of *yjgB* and cloned it into the expression vector pET-28a(+). The construct was then transformed into the *E. coli* strain BL21(DE3). The transformed cells were cultured in 10 mL of LB medium supplemented with 100 µg/mL kanamycin and incubated overnight at 37°C with shaking at 220 rpm. The overnight culture was diluted 1:100 into fresh LB medium, with ZnCl_2_ added to a final concentration of 0.1 mM. The culture was incubated at 37°C for 3 h at 220 rpm, followed by the addition of 0.5 mM isopropyl beta-D-thiogalactopyranoside and further incubation at 15°C for 21 h at 150 rpm. The cells were harvested via centrifugation and resuspended in 25 mM Tris-HCl buffer (pH 8.0) containing 300 mM NaCl and 10% glycerol. The crude extract was prepared via a cell disruptor and centrifuged at 8,000 rpm for 30 min at 4°C to remove insoluble material. The fusion protein was purified via a HisTrap affinity column (#SA035010, Smart-Lifesciences) according to the manufacturer’s instructions, and the eluted fractions were analyzed via SDS-PAGE.

### Enzyme activity assay

The assay was conducted with 10 µM purified protein (final concentration) in reaction buffer with a total volume of 100 µL. The reaction mixture contained 50 mM Tris-HCl and 0.2 mM NADPH, pH 8.0. The reaction was initiated by adding indole-3-acetaldehyde to a final concentration of 200 µM as the substrate and incubated at 37°C. The products were analyzed using a Shimadzu LC-20AT HPLC system equipped with a UV/Vis detector set to 210 nm. Separation was performed on a reversed-phase T3 Waters column (2.1 × 40 mm; 5 µm) at a flow rate of 1 mL/min. The mobile phase consisted of 10% acetonitrile and 90% water.

### RNA-Seq and RT-qPCR analysis

The bacterial cells were cultured to an OD_600_ of 1.0 and then harvested in biological triplicate using three independent cultures per condition. RNA extraction was performed via the Eastep Super Total RNA Extraction Kit (Promega Corporation, Madison, USA). cDNA synthesis and RT-qPCR analysis were performed with three technical replicates per biological replicate using the ChamQ Universal SYBR qPCR Master Mix (Vazyme, Nanjing, China) on a 7300 Plus Real-Time PCR System (Thermo Fisher Scientific, New York, USA) following the manufacturer’s instructions. The expression levels of target genes in each experiment were normalized to the transcription levels of 16S RNA. Relative expression levels of target genes were calculated via the comparative CT (2^−ΔΔCT^) method (62). Double-stranded cDNA synthesis and high-throughput RNA-Seq were performed according to established protocols (63). For RNA-Seq library preparation, two biological replicates per strain/condition were independently processed through parallel sequencing runs. For RNA-Seq data analysis, the index of the reference genome (GenBank accession numbers CP053751 and GCA_000025565.1) was built using Bowtie2 v2.2.6, and paired-end clean reads were aligned to the reference genome via Bowtie2. A *q* value (false discovery rate) of <0.05 and a fold change of >1 were used to identify the significantly differentially expressed genes. The *hisG* gene of *S. sonnei* and the *rpoB gene* of *E. cloacae* were used as internal controls.

### Statistical analysis

No statistical method was used to predetermine the sample size. No data were excluded from the analyses. The data are presented as the means ± SDs. Statistical analyses were performed via GraphPad Prism 10 software (version 10.3.1). *P* < 0.05 was considered statistically significant. ns, not significant. Statistical significance was determined on the basis of precise *P* values obtained from one-way ANOVA or unpaired *t* tests. Biological replicates and numbers of independent experiments are provided in the legends. All experiments presented as representative HPLC graphs or gels were repeated at least three times with similar results.

## Data Availability

The RNA-Seq data generated in this study have been deposited in the NCBI Sequence Read Archive (SRA) database under accession numbers PRJNA1227863 and PRJNA1227901. Data supporting the findings of this study are available within the paper and its supplemental material.

## SUPPLEMENTAL MATERIAL

Supplemental material aem.00510-25-s0001.docx Figures S1 to S11; Tables S1 to S5.

ASM does not own the copyrights to Supplemental Material that may be linked to, or accessed through, an article. The authors have granted ASM a non-exclusive, world-wide license to publish the Supplemental Material files. Please contact the corresponding author directly for reuse.
