# Supplementary material for: A cell-cell communication signal from Enterobacter cloacae interfering with the signaling systems and virulence in Shigella sonnei
Source: Appl Environ Microbiol. 2025 May 12;91(6):e00510-25. doi: 10.1128/aem.00510-25 (PMC12175533; doi:10.1128/aem.00510-25)
Supplement: Supplemental material — Figures S1 to S11; Tables S1 to S5. [file aem.00510-25-s0001.docx]

**Supporting Information**

**A cell-cell communication signal from *Enterobacter cloacae* interfering with the signaling systems and virulence in *Shigella sonnei***

Xiayu Chen^1,4^, Mingfang Wang^1,2,4^, Zhuoxian Zhao^1^, Xiwen Ling^1^, Ganjin Peng^1^, Binbin Cui^1,3^, Qiaoping Wang^1^, Bing Gu^2^, Yinyue Deng^1^*

*^1^School of Pharmaceutical Sciences (Shenzhen), Shenzhen Campus of Sun Yat-sen University, Sun Yat-sen University, Shenzhen 518107, China*

*^2^Department of Clinical Laboratory Medicine,Guangdong Provincial People’s Hospital (GuangdongAcademy of Medical Sciences), Southern Medical University,Guangzhou, Guangdong 510000, China*

*^3^Pharmacy Department, The Affiliated LiHuiLi Hospital of Ningbo University, Ningbo 315046, China*

*^4^These authors contributed equally:* *Xiayu Chen and* *Mingfang Wang.*

**Corresponding author:**

Yinyue Deng: dengyle@mail.sysu.edu.cn

**Running title: Indole-3-ethanol is a QS signal in *E. cloacae***

**Supplementary Figures and Tables**


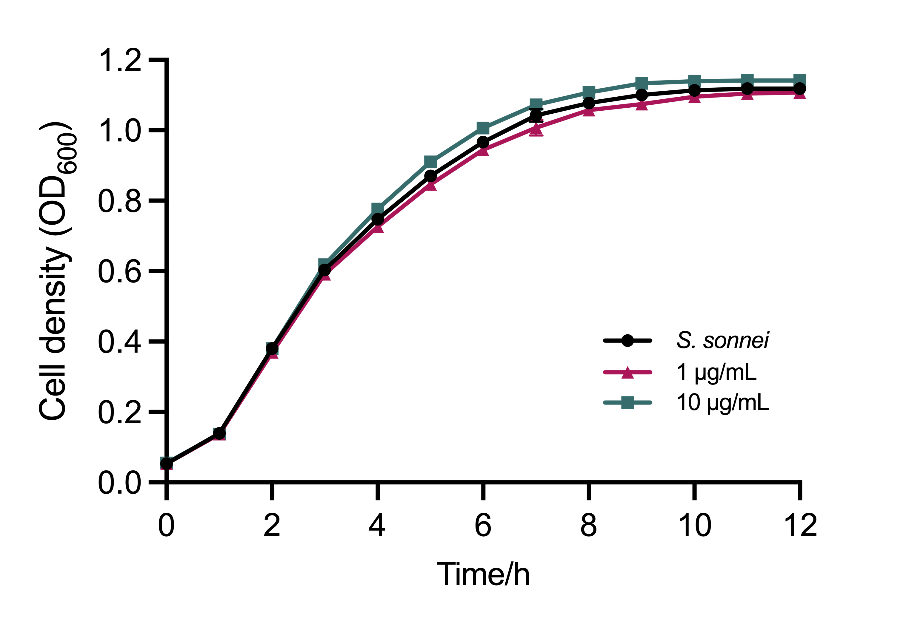


**Supplementary Figure 1.** **The growth curve of *S. sonnei* in the absence and presence of the *E. cloacae* extract.** The data are presented as the means ± SDs and are representative of three independent experiments. The error bars indicate SDs. The source data are provided as a source data file.


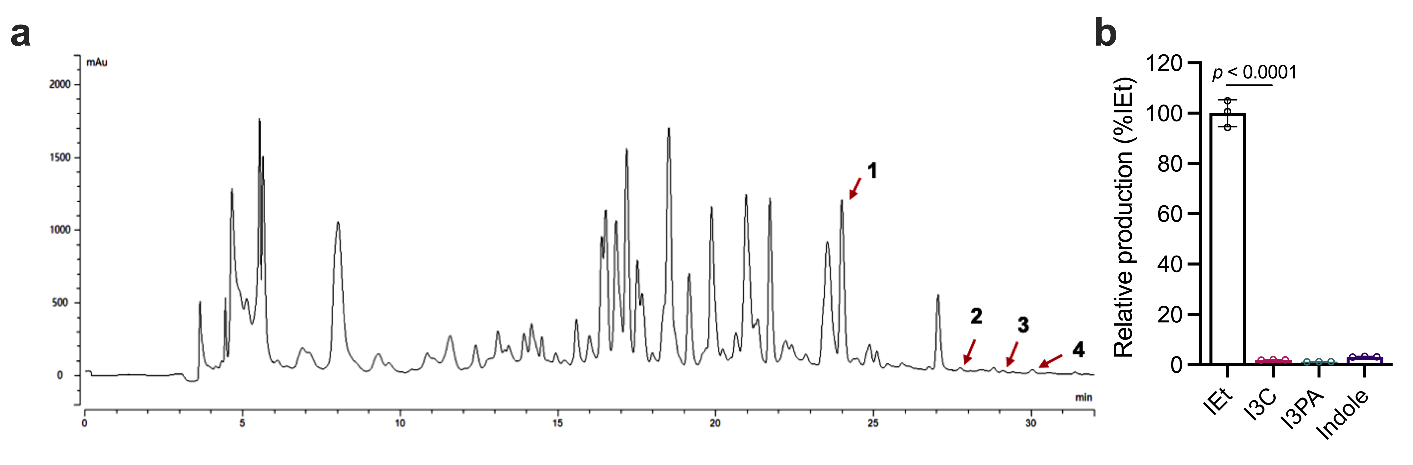


**Supplementary Figure 2.** **Active compounds isolated from *E. cloacae* extract.** **a** Chromatogram of the bioactive fractions of *E. cloacae* extract. Peak 1: IEt; peak 2: I3C; peak 3: I3PA; peak 4: indole. **b** Comparison of the relative production of the bioactive fractions of *E. cloacae* extract. Indole-3-ethanol: IEt, indole-3-pyruvic acid: I3PA, indole-3-carbinol: I3C. The data are presented as the means ± SDs and are representative of three independent experiments. The error bars indicate SDs. *P* values reflect one-way ANOVA tests used to determine the significance of the results. The source data are provided as a source data file.


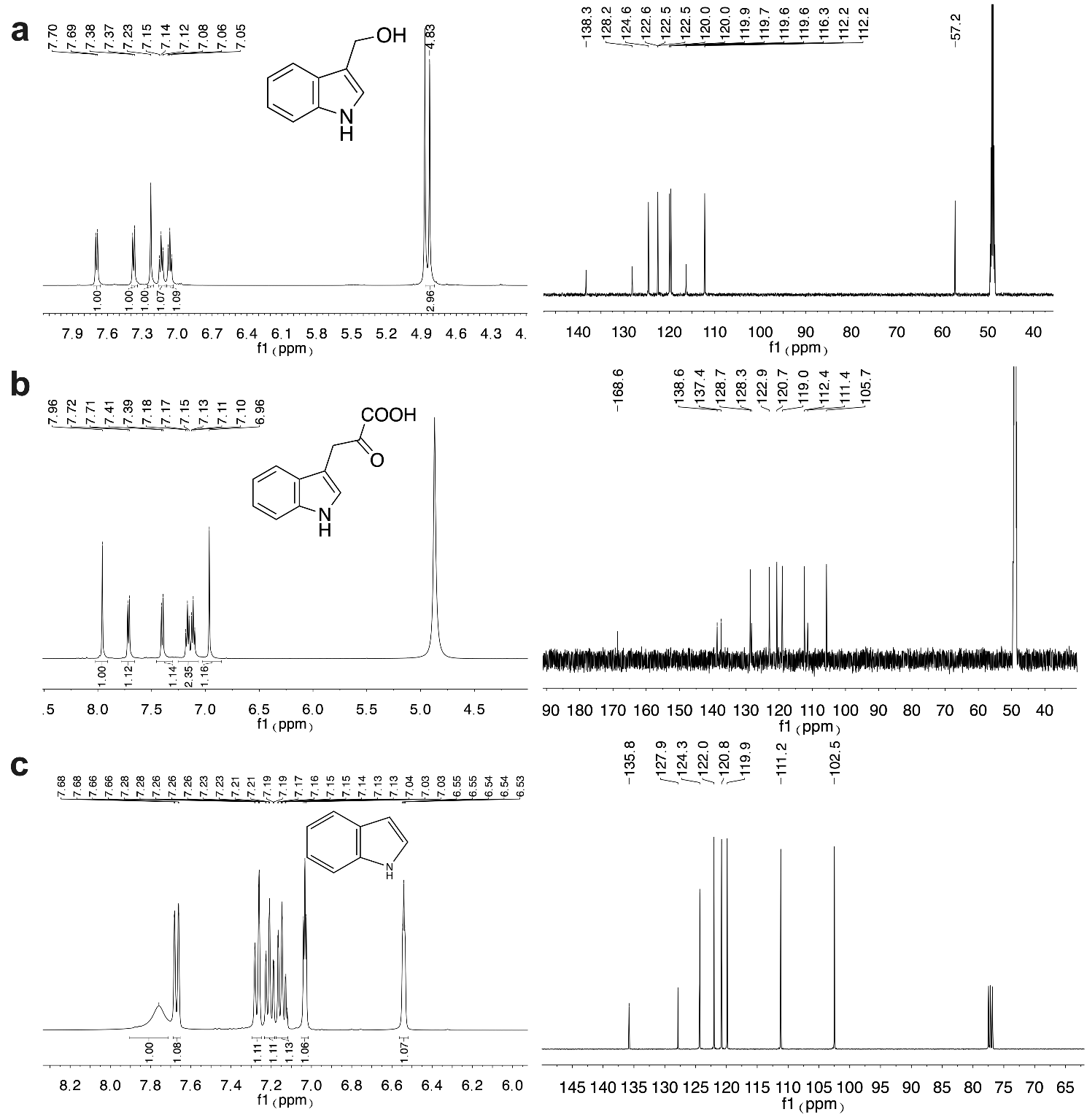


**Supplementary Figure 3. Structural characterization of indole and its derivatives. a** ^1^H and ^13^C NMR spectra of indole-3-carbinol. **b** ^1^H and ^13^C NMR spectra of indole-3-pyruvic acid. **c** ^1^H and ^13^C NMR spectra of indole.

**
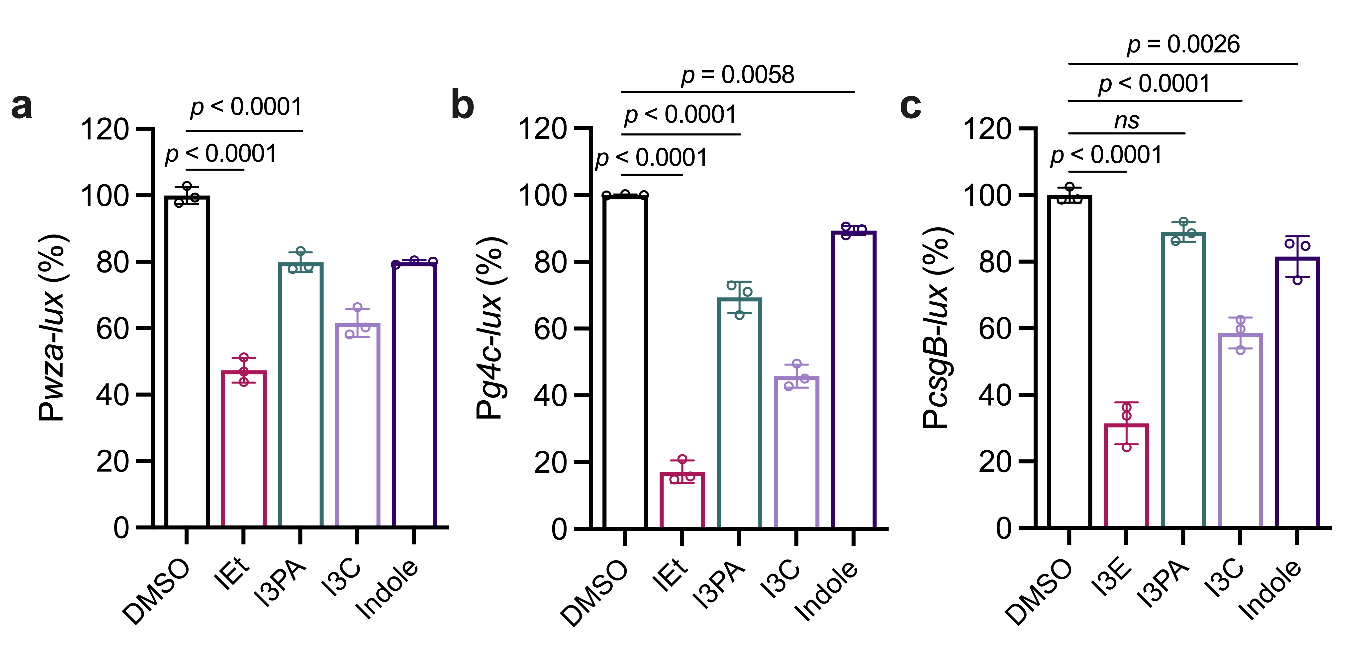
**

**Supplementary Figure 4. Effects of indole and its derivatives on the transcription of EPS biosynthesis-related and biofilm-related genes in *S. sonnei*.** The transcriptional expression of EPS biosynthesis-related and biofilm-related genes, *wza* (**a**), *g4c* (**b**), and *csgB* (**c**), were analyzed in the absence and presence of 100 µM indole-3-ethanol, indole-3-pyruvic acid, indole-3-carbinol, and indole, respectively. The gene expression levels of *wza*, *g4c* and *csgB* were evaluated by assessing light production (counts per second [cps]) by *wza-luxCDABE*, *g4c*-*luxCDABE* and *csgB-luxCDABE* transcriptional fusions in the *S. sonnei* strains. Indole-3-ethanol: IEt, indole-3-pyruvic acid: I3PA, indole-3-carbinol: I3C. The data are presented as the means ± SDs and are representative of three independent experiments. The error bars indicate SDs. *P* values reflect one-way ANOVA tests used to determine the significance of the results. The source data are provided as a source data file.

**
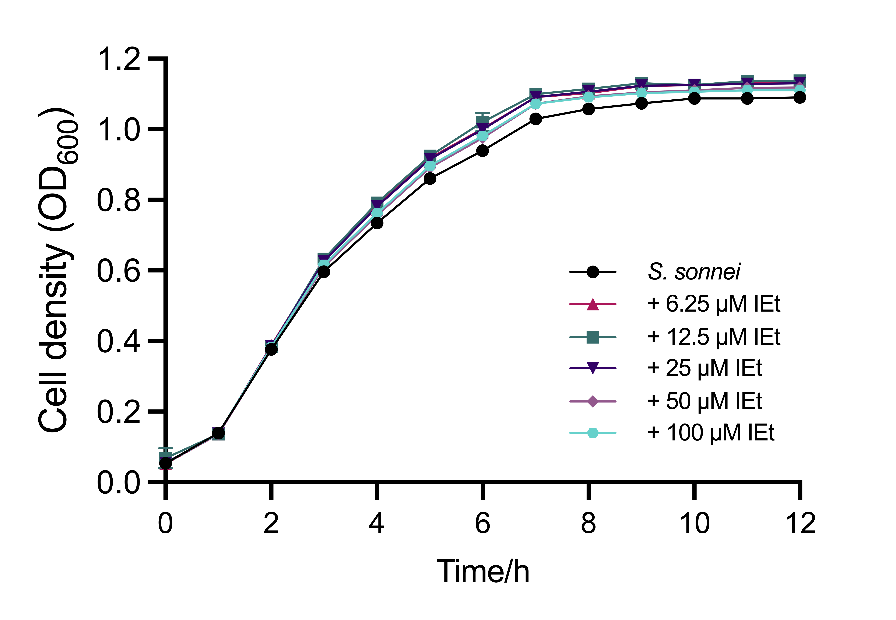
**

**Supplementary Figure 5. Growth curves of *S. sonnei* treated with different concentrations of indole-3-ethanol.** Indole-3-ethanol: IEt. The data are presented as the means ± SDs and are representative of three independent experiments. The error bars indicate SDs. The source data are provided as a source data file.


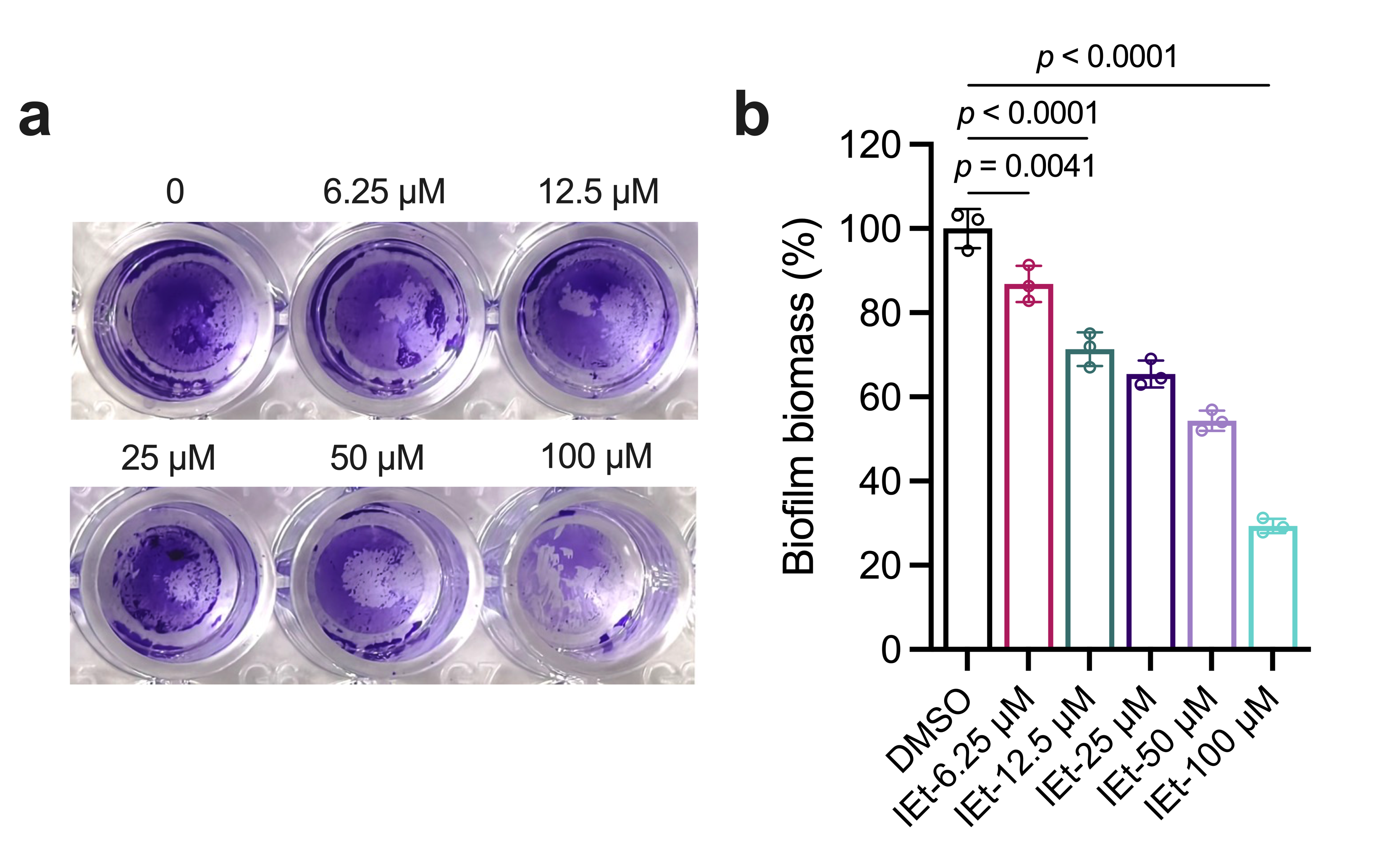


**Supplementary Figure 6. Effects of different concentrations of indole-3-ethanol on *S. sonnei* biofilm dispersion. a** Representative images of *S. sonnei* formed biofilms treated with different concentrations of indole-3-ethanol (0, 6.25, 12.5, 25, 50, and 100 µM) for 4 hours at 37°C, stained with crystal violet. **b** Relative quantitative analysis of biofilm biomass using the crystal violet assay, expressed as a percentage relative to the untreated control (DMSO). Indole-3-ethanol: IEt. The data are presented as mean ± SDs and are representative of three independent experiments. The error bars indicate SDs. *P* values reflect one-way ANOVA tests used to determine the significance of the results. The source data are provided as a source data file.


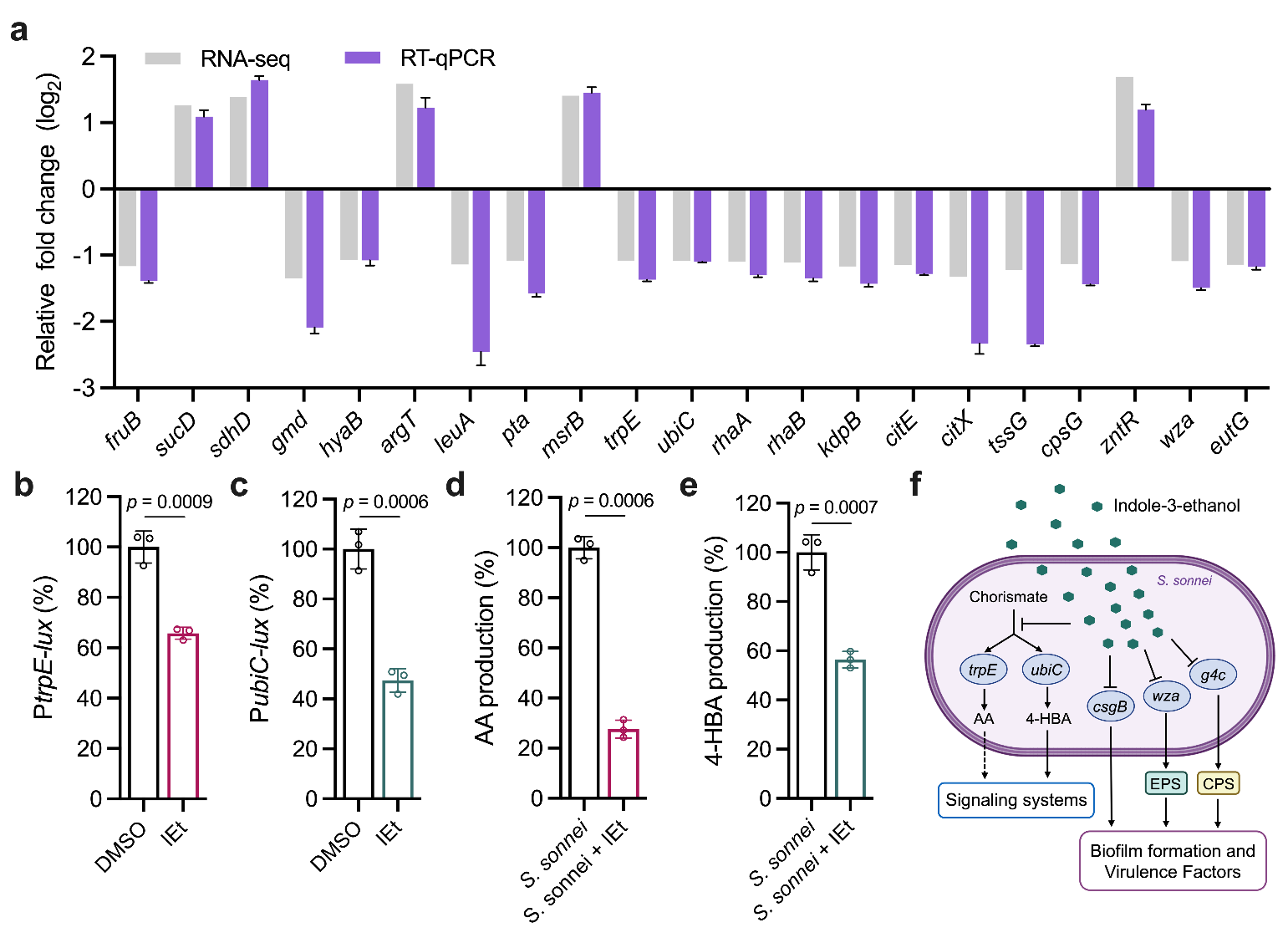


**Supplementary Figure 7.** **Impact of exogenous indole-3-ethanol on pathogenic phenotypes and signal production in *S. sonnei***. **a** RT‒qPCR results showing that the expression levels of genes related to biofilm formation and EPS biosynthesis were significantly decreased in *S. sonnei* treated with 100 μM indole-3-ethanol. The transcriptional expression of *trpE* (**b**) and *ubiC* (**c**), were analyzed in the absence and presence of 100 µM indole-3-ethanol. The gene expression levels of *trpE* and *ubiC* were evaluated by assessing light production (counts per second [cps]) by *trpE-luxCDABE* and *ubiC*-*luxCDABE* transcriptional fusions in the *S. sonnei* strains. Effect of the addition of exogenous indole-3-ethanol on the production of anthranilic acid (**d**) and 4-hydroxybenzoic acid (**e**) in *S. sonnei*, with DMSO used as a control. **f** A schematic diagram of the signal transduction pathways involved in biofilm formation, EPS, and CPS synthesis. The blue border indicates that the expression level of the gene encoding this protein was significantly inhibited by indole-3-ethanol. Indole-3-ethanol: IEt, anthranilic acid: AA, 4-hydroxybenzoic acid: 4-HBA. CPS: capsular polysaccharide. The data are presented as the means ± SDs and are representative of three independent experiments. The error bars indicate SDs. *P* values reflect unpaired t tests used to determine the significance of the results. The source data are provided as a source data file.


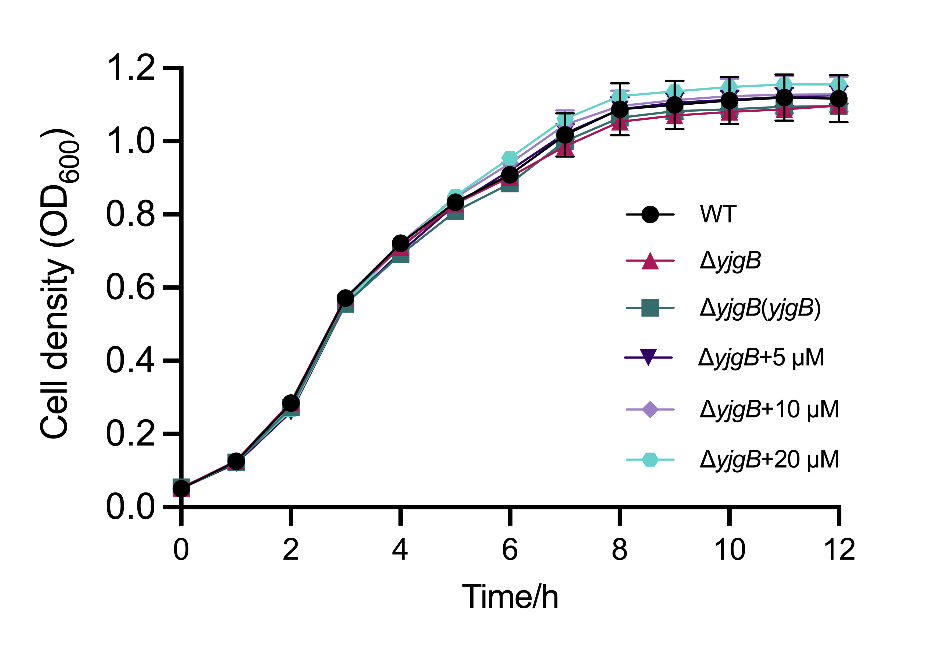


**Supplementary Figure 8. The growth curves of *E. cloacae*, the *yjgB* deletion mutant, the complementary strain, and the *yjgB* deletion mutant with the addition of different concentrations of indole-3-ethanol.** The data are presented as the means ± SDs and are representative of three independent experiments. The error bars indicate SDs. The source data are provided as a source data file.


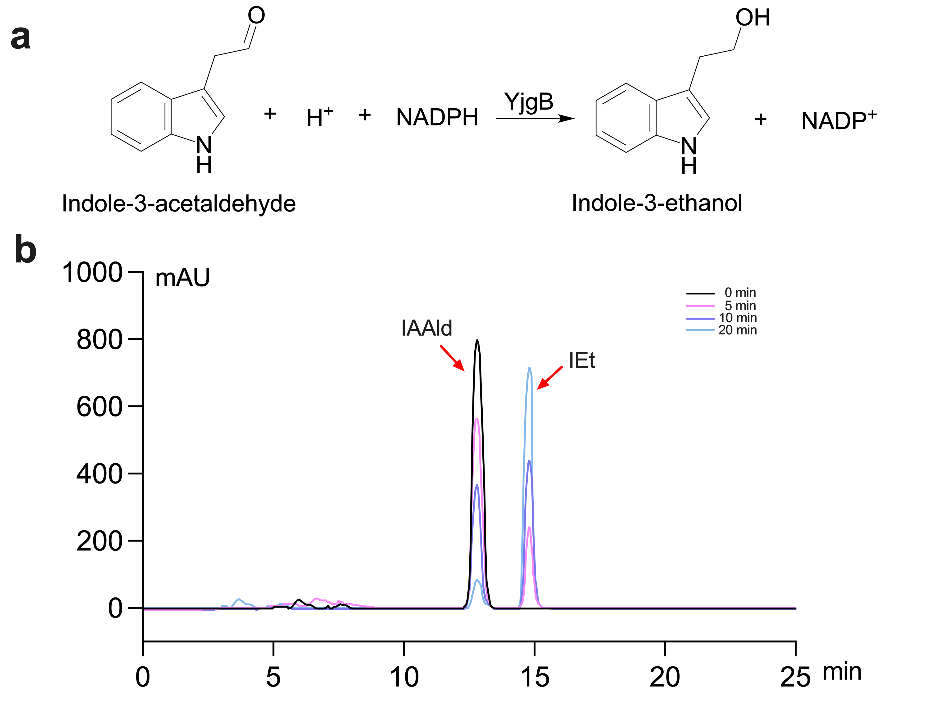


**Supplementary Figure 9. Catalytic process mediated by YjgB.** **a** Synthesis of indole-3-ethanol catalyzed by YjgB. **b** HPLC analysis of the YjgB-catalyzed conversion of indole-3-acetaldehyde (peak 1, IAAld) to indole-3-ethanol (peak 2, IEt).


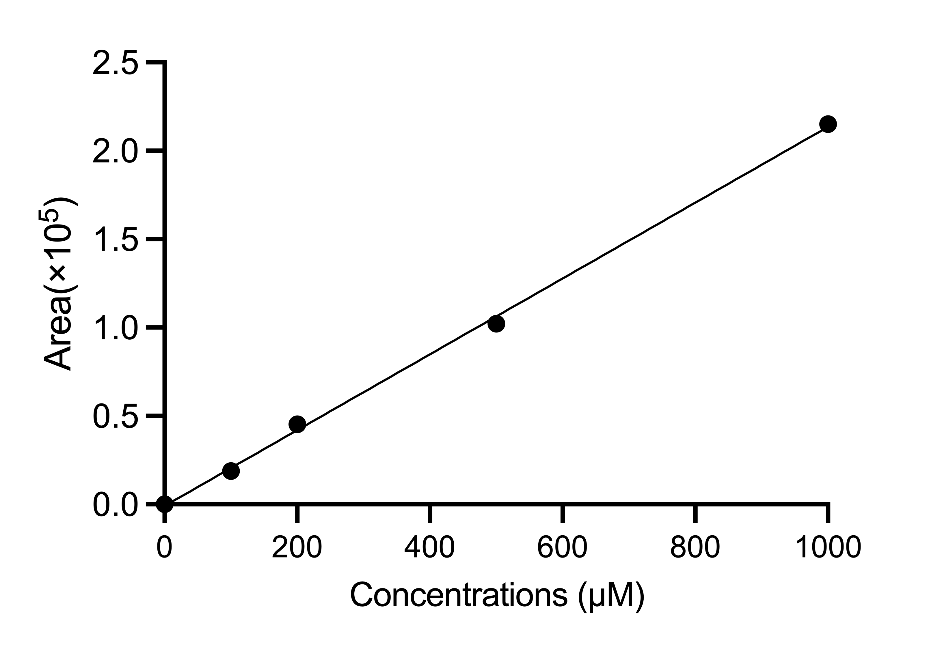


**Supplementary Figure 10. Calibration curves were generated by plotting the peak area (Y) versus the concentration (X, μM) of the standard solutions of indole-3-ethanol.** The regression equation of indole-3-ethanol was *Y*=21.45*X*-87.37, and the linear *R^2^*=0.9988 (*n*=5). The source data are provided as a source data file.

**
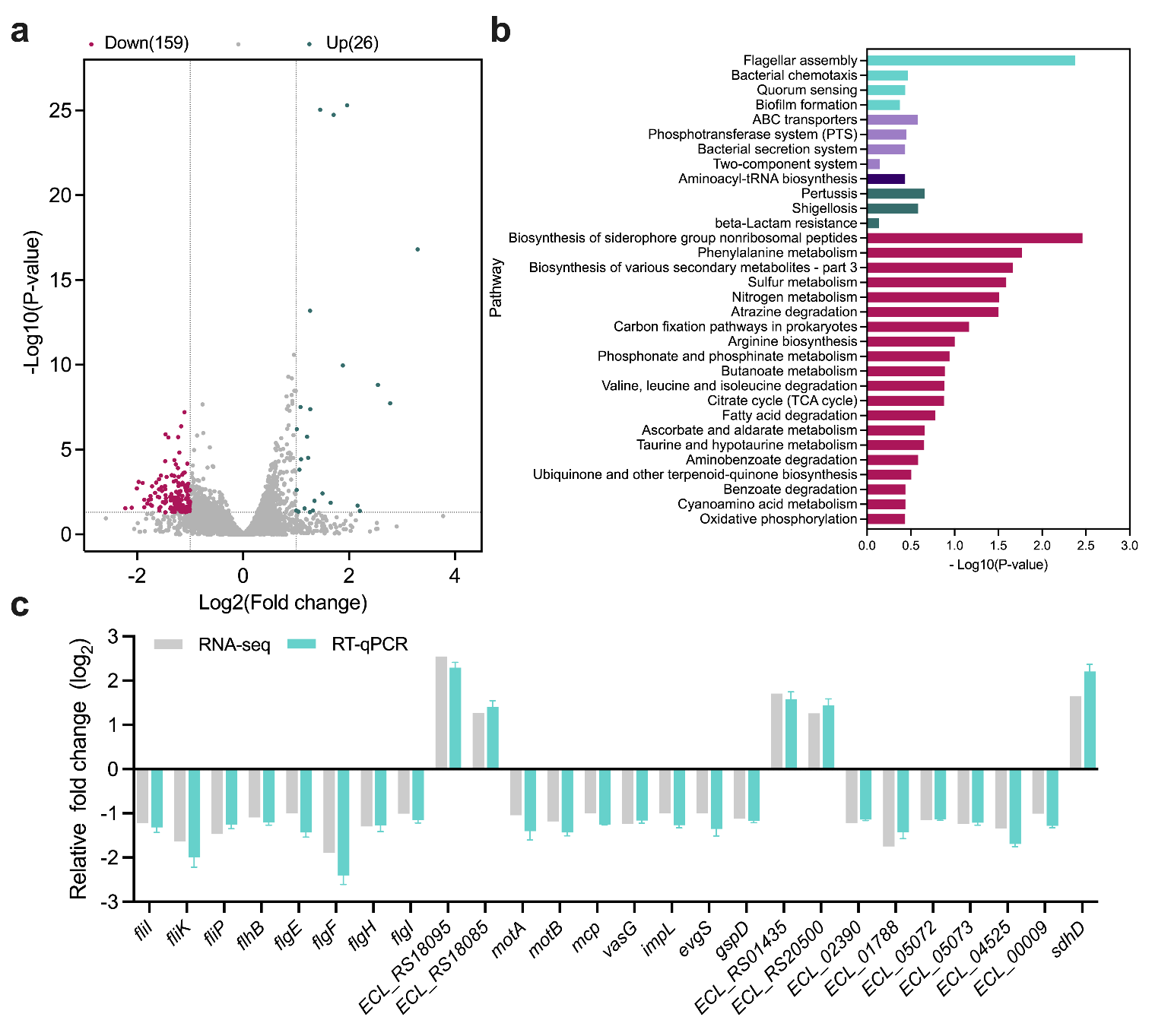
**

**Supplementary Figure** **11. Differential gene expression profiles between the *yjgB* deletion mutant strain and the wild-type strain as measured by RNA-Seq (log2-fold change ≥1). a** The number of genes upregulated and downregulated in the Δ*yjgB* strain compared with the wild-type strain. **b** GO term enrichment analysis of genes differentially expressed between the ∆*yjgB* and wild-type strains. **c** RT‒qPCR analysis of the genes whose expression differed between the *yjgB* deletion mutant strain and the wild-type strain. The data are presented as the means ± SDs and are representative of three independent experiments. The error bars indicate SDs. The source data are provided as a source data file.

**Supplementary Table 1** List of differentially expressed genes after the addition of indole-3-ethanol or DMSO in *S. sonnei* (Log_2_-fold change ≥ 1). Significantly differentially expressed genes were identified via Cufflinks after Benjamini‒Hochberg correction. The fold change is the ratio of gene expression with the addition of indole-3-ethanol to that with the addition of DMSO, measured in FPKM.

| **Gene ID^a^** | **Log_2_-fold change** | **Description** |
| --- | --- | --- |
| COO90_RS11635 | -4.809907155 | YobH family protein |
| COO90_RS03200 | -2.638781145 | RpiB/LacA/LacB family sugar-phosphate isomerase |
| COO90_RS18270 | -2.358119013 | ethanolamine utilization microcompartment protein EutN |
| COO90_RS15745 | -1.635169619 | BMC domain-containing protein |
| COO90_RS14040 | -1.590024574 | Ail/Lom family outer membrane beta-barrel protein |
| COO90_RS17645 | -1.577346198 | phage tail protein |
| COO90_RS09485 | -1.469284011 | NADPH-dependent FMN reductase |
| COO90_RS18950 | -1.43704154 | IS66 family transposase |
| COO90_RS18450 | -1.425725955 | hydrogenase 4 assembly chaperone HyfJ |
| COO90_RS09955 | -1.414598369 | NADH-dependent FMN reductase RutF |
| COO90_RS19550 | -1.411543849 | phage terminase small subunit P27 family |
| COO90_RS04950 | -1.379590323 | electron transfer flavoprotein FixA |
| COO90_RS19360 | -1.371343981 | prophage tail fiber N-terminal domain-containing protein |
| COO90_RS15950 | -1.347883994 | GDP-mannose 4,6-dehydratase |
| COO90_RS07570 | -1.321525091 | citrate lyase holo-[acyl-carrier protein] synthase |
| COO90_RS19325 | -1.318665748 | phage GP46 family protein |
| COO90_RS12990 | -1.30997531 | ATP-binding protein |
| COO90_RS17980 | -1.295863043 | PTS fructose transporter subunit IIC |
| COO90_RS00975 | -1.257364153 | cellulose biosynthesis protein BcsQ |
| COO90_RS17905 | -1.252470206 | formyl-CoA transferase |
| COO90_RS04920 | -1.251846468 | crotonobetainyl-CoA hydratase |
| COO90_RS20115 | -1.240291817 | HPr family phosphocarrier protein |
| COO90_RS07415 | -1.231005188 | enterochelin esterase |
| COO90_RS20575 | -1.230714613 | DUF2509 family protein |
| COO90_RS05975 | -1.221238738 | type VI secretion system baseplate subunit TssG |
| COO90_RS04230 | -1.212606365 | iron-dicitrate ABC transporter permease FecC |
| COO90_RS18290 | -1.211014145 | ethanolamine utilization acetate kinase EutQ |
| COO90_RS06445 | -1.206269025 | taurine ABC transporter substrate-binding protein |
| COO90_RS20025 | -1.205234144 | formate hydrogenlyase subunit HycB |
| COO90_RS02875 | -1.196733523 | capsule biosynthesis GfcC family protein |
| COO90_RS09965 | -1.19228782 | pyrimidine utilization protein D |
| COO90_RS06455 | -1.185489344 | taurine ABC transporter permease TauC |
| COO90_RS07980 | -1.166987162 | potassium-transporting ATPase subunit KdpB |
| COO90_RS16590 | -1.163384304 | fused PTS fructose transporter subunit IIA/HPr protein |
| COO90_RS17610 | -1.161182000 | phage minor tail protein L |
| COO90_RS07580 | -1.149210859 | citrate (pro-3S)-lyase subunit beta |
| COO90_RS18255 | -1.148997983 | ethanolamine utilization ethanol dehydrogenase EutG |
| COO90_RS13360 | -1.147986409 | ABC transporter permease |
| COO90_RS23395 | -1.142655118 | aminotransferase class I/II-fold pyridoxal phosphate-dependent enzyme |
| COO90_RS06450 | -1.141601464 | taurine ABC transporter ATP-binding subunit |
| COO90_RS05115 | -1.14064242 | 2-isopropylmalate synthase |
| COO90_RS18150 | -1.140448421 | sulfate/thiosulfate ABC transporter permease CysW |
| COO90_RS15925 | -1.130009317 | colanic acid biosynthesis phosphomannomutase CpsG |
| COO90_RS00180 | -1.126483434 | transcriptional regulator UhpA |
| COO90_RS00900 | -1.124004142 | dipeptide ABC transporter permease DppC |
| COO90_RS23960 | -1.120881915 | nickel ABC transporter permease subunit NikC |
| COO90_RS03800 | -1.119862014 | L-ribulose-5-phosphate 3-epimerase UlaE |
| COO90_RS18230 | -1.118103178 | ethanolamine utilization microcompartment protein EutL |
| COO90_RS18755 | -1.11649941 | ABC transporter permease |
| COO90_RS19690 | -1.116391847 | L-2-hydroxyglutarate oxidase |
| COO90_RS07470 | -1.115156488 | enterobactin biosynthesis bifunctional isochorismatase/aryl carrier protein EntB |
| COO90_RS02205 | -1.110967735 | rhamnulokinase |
| COO90_RS03090 | -1.106664304 | guanine/hypoxanthine transporter GhxP |
| COO90_RS04955 | -1.10613557 | electron transfer flavoprotein subunit alpha/FixB family protein |
| COO90_RS20010 | -1.102024844 | formate hydrogenlyase subunit HycE |
| COO90_RS04430 | -1.101135863 | 5-carboxymethyl-2-hydroxymuconate semialdehyde dehydrogenase |
| COO90_RS02200 | -1.093866226 | L-rhamnose isomerase |
| COO90_RS06295 | -1.093843805 | carbamate kinase family protein |
| COO90_RS10350 | -1.092051567 | flagellar basal body P-ring protein FlgI |
| COO90_RS15995 | -1.089031827 | polysaccharide export protein Wza |
| COO90_RS10355 | -1.087877661 | flagellar assembly peptidoglycan hydrolase FlgJ |
| COO90_RS02955 | -1.084423937 | chorismate lyase |
| COO90_RS20020 | -1.083142706 | formate hydrogenlyase subunit 3 |
| COO90_RS14670 | -1.081367215 | anthranilate synthase component I |
| COO90_RS18280 | -1.08090382 | phosphate acetyltransferase |
| COO90_RS09680 | -1.070753022 | Ni/Fe-hydrogenase large subunit |
| COO90_RS21745 | -1.067895512 | L-tartrate/succinate antiporter |
| COO90_RS10035 | 1.237866577 | phosphate starvation-inducible protein PhoH |
| COO90_RS08140 | 1.265737201 | succinate--CoA ligase subunit alpha |
| COO90_RS08110 | 1.389788458 | succinate dehydrogenase membrane anchor subunit |
| COO90_RS11920 | 1.404462277 | peptide-methionine (R)-S-oxide reductase MsrB |
| COO90_RS02320 | 1.410320526 | septal ring assembly protein ZapB |
| COO90_RS17355 | 1.58609851 | lysine/arginine/ornithine ABC transporter substrate-binding protein ArgT |
| COO90_RS23010 | 1.692563619 | Zn(2+)-responsive transcriptional regulator |
| COO90_RS14560 | 2.019851965 | stress response translation initiation inhibitor YciH |
| COO90_RS09495 | 2.694528074 | fimbrial protein |
| COO90_RS18765 | 2.893392619 | substrate-binding domain-containing protein |
| COO90_RS17580 | 3.801341706 | tail fiber assembly protein |
| COO90_RS13005 | 5.664925049 | hypothetical protein |
| COO90_RS10895 | 5.834409136 | DUF2594 family protein YecF |
| COO90_RS16115 | 5.87843513 | protein YegR |

^a^ Gene ID is cited from https://ftp.ncbi.nlm.nih.gov/genomes/all/GCF/013/201/485/GCF_013201485.1_ASM1320148v1/. Annotations and functional assignments are from *Shigella sonnei* strain 506.

**Supplementary Table 2** List of genes differentially expressed in the *yjgB* deletion mutant compared with the wild-type strain (Log_2_-fold change ≥ 1). Significantly differentially expressed genes were identified via Cufflinks after Benjamini‒Hochberg correction. The fold change is the ratio of the mutant FPKM to the wild-type FPKM.

| **Gene ID^b^** | **Log_2_-fold change** | **Description** |
| --- | --- | --- |
| *ECL_RS06595* | -2.221725685 | hypothetical protein |
| *ECL_RS16725* | -2.103112072 | putative colanic acid biosynthesis acetyltransferase WcaB |
| *ECL_RS03550* | -2.003883789 | amino acid transporter LysE |
| *ECL_RS01705* | -1.971458765 | ribose 1,5-bisphosphokinase |
| *ECL_RS16170* | -1.895092154 | Flagellar basal-body rod protein FlgF |
| *ECL_RS03415* | -1.865074021 | DoxX family protein |
| *ECL_RS02050* | -1.86465716 | type IV pilus biogenesis protein PilP |
| *ECL_RS20375* | -1.806394022 | putative flavoprotein |
| *ECL_RS01735* | -1.780916348 | carbon-phosphorus lyase complex subunit |
| *ECL_RS08815* | -1.747695108 | beta-phosphoglucomutase |
| *ECL_RS10550* | -1.744906251 | enoyl-CoA hydratase |
| *ECL_RS07020* | -1.739326604 | hypothetical protein |
| *ECL_RS08440* | -1.722033285 | gp18 |
| *ECL_RS15335* | -1.71711252 | iron-enterobactin transporter ATP-binding protein |
| *ECL_RS07915* | -1.660603463 | nitrate ABC transporter, inner membrane subunit |
| *ECL_RS16285* | -1.639709584 | flagellar hook-length control protein |
| *ECL_RS07910* | -1.611684836 | nitrate ABC transporter, substrate-binding protein |
| *ECL_RS21870* | -1.588355289 | urease accessory protein UreD |
| *ECL_RS11750* | -1.586039353 | iron complex transport system substrate-binding protein |
| *ECL_RS13015* | -1.584839817 | allantoate transport membrane protein |
| *ECL_RS02200* | -1.582897066 | hypothetical protein |
| *ECL_RS05030* | -1.580185609 | hypothetical protein |
| *ECL_RS20925* | -1.56075929 | hypothetical protein |
| *ECL_RS15305* | -1.551525045 | enterobactin 2,3-dihydroxybenzoate-AMP ligase/S-dihydroxybenzoyltransferase |
| *ECL_RS07500* | -1.541247076 | putative ABC phosphate/phosphonate transporter, periplasmic ligand binding protein |
| *ECL_RS10205* | -1.530741341 | RtxD/HlyD/CyaD protein of type I secretion system |
| *ECL_RS21420* | -1.514667779 | phosphoribosyl-dephospho-CoA transferase |
| *ECL_RS10795* | -1.476014126 | putative ABC transporter, ATP-binding protein |
| *ECL_RS10940* | -1.4734656 | gamma-glutamylputrescine oxidoreductase |
| *ECL_RS21230* | -1.469103235 | major facilitator superfamily transporter |
| *ECL_RS16260* | -1.46761069 | Flagellar biosynthetic protein FliP |
| *ECL_RS15255* | -1.46633898 | putative alcohol dehydrogenase |
| *ECL_RS08305* | -1.462261043 | phage portal protein, lambda family |
| *ECL_RS21445* | -1.462217009 | 5-oxopent-3-ene-1,2,5-tricarboxylate decarboxylase |
| *ECL_RS13120* | -1.457496563 | iron complex transport system ATP-binding protein |
| *ECL_RS05470* | -1.453414962 | sulfonate/nitrate/taurine transport system substrate-binding protein |
| *ECL_RS20210* | -1.443800246 | nitric oxide reductase |
| *ECL_RS21440* | -1.410080098 | citrate lyase ligase |
| *ECL_RS01720* | -1.378133308 | phosphonate C-P lyase system protein PhnK |
| *ECL_RS11770* | -1.374417289 | 3-oxoadipate CoA-transferase subunit A |
| *ECL_RS00460* | -1.366430375 | fimbrial protein FimI |
| *ECL_RS09215* | -1.36308599 | luciferase family protein |
| *ECL_RS14495* | -1.355030632 | putative amino acid transporter |
| *ECL_RS09460* | -1.352505356 | peptide/nickel transport system permease protein |
| *ECL_RS21715* | -1.344873596 | iron complex transport system permease protein |
| *ECL_RS22560* | -1.341877082 | tagatose-6-phosphate ketose/aldose isomerase |
| *ECL_RS01980* | -1.341860888 | hypothetical protein |
| *ECL_RS08445* | -1.336291976 | gp19 |
| *ECL_RS15845* | -1.321476571 | phage antitermination protein Q |
| *ECL_RS16880* | -1.308336328 | hypothetical protein |
| *ECL_RS21210* | -1.304492806 | L-aspartate dehydrogenase |
| *ECL_RS21235* | -1.300688285 | putative ferredoxin reductase subunit |
| *ECL_RS16160* | -1.299235859 | Flagellar L-ring protein, FlgH |
| *ECL_RS22470* | -1.296157474 | hypothetical protein |
| *ECL_RS08785* | -1.295303275 | multiple sugar transport system permease protein |
| *ECL_RS02065* | -1.290886238 | type IV prepilin |
| *ECL_RS05485* | -1.288813047 | taurine dioxygenase |
| *ECL_RS11780* | -1.285850799 | acetyl-CoA acetyltransferase |
| *ECL_RS05020* | -1.271002588 | hypothetical protein |
| *ECL_RS16375* | -1.270724398 | aerobactin siderophore biosynthesis protein LucB |
| *ECL_RS21205* | -1.268970207 | putative aldehyde dehydrogenase |
| *ECL_RS03120* | -1.266850095 | putative pyridoxal phosphate-dependent enzyme |
| *ECL_RS09640* | -1.264924335 | copper/silver efflux system outer membrane protein CusC |
| *ECL_RS16720* | -1.264392985 | putative glycosyl transferase |
| *ECL_RS08900* | -1.244546342 | type VI secretion system protein VasG |
| *ECL_RS05035* | -1.238942641 | hypothetical protein |
| *ECL_RS25295* | -1.238841345 | L-rhamnose isomerase |
| *ECL_RS16685* | -1.238058691 | colanic acid biosynthesis glycosyl transferase WcaI |
| *ECL_RS21220* | -1.231864999 | putative hydrolase |
| *ECL_RS21900* | -1.231030935 | urease accessory protein UreF |
| *ECL_RS02045* | -1.228820205 | pilin accessory protein PilO |
| *ECL_RS21215* | -1.22658335 | putative short chain dehydrogenase |
| *ECL_RS16090* | -1.226484588 | extracellular solute-binding protein |
| *ECL_RS21425* | -1.224854076 | citrate lyase, alpha subunit |
| *ECL_RS13275* | -1.224124095 | alkanesulfonate monooxygenase |
| *ECL_RS20255* | -1.221832395 | NADH ubiquinone oxidoreductase |
| *ECL_RS20270* | -1.221147059 | formate hydrogenlyase subunit 4 |
| *ECL_RS16295* | -1.219919026 | flagellum-specific ATP synthase |
| *ECL_RS19860* | -1.216389861 | TolC family type I secretion outer membrane protein |
| *ECL_RS11710* | -1.215401271 | putative glycoside hydrolase |
| *ECL_RS20265* | -1.204222783 | NADH dehydrogenase (ubiquinone) |
| *ECL_RS16370* | -1.203820681 | Aerobactin siderophore biosynthesis protein IucA |
| *ECL_RS24270* | -1.198652742 | hypothetical protein |
| *ECL_RS19870* | -1.197914778 | HlyD family type I secretion membrane fusion protein |
| *ECL_RS16655* | -1.195456032 | colanic acid biosynthesis glycosyl transferase WcaL |
| *ECL_RS13090* | -1.188365225 | putative flavoprotein monooxygenase |
| *ECL_RS01730* | -1.18686871 | phosphonate metabolism PhnI |
| *ECL_RS15580* | -1.186724395 | major facilitator transporter |
| *ECL_RS16110* | -1.183868314 | chemotaxis protein MotB |
| *ECL_RS15350* | -1.180463302 | enterobactin/ferric enterobactin esterase |
| *ECL_RS10780* | -1.179398035 | putative allophanate hydrolase |
| *ECL_RS26730* | -1.173860776 | TniA protein |
| *ECL_RS15320* | -1.173393234 | enterobactin exporter EntS |
| *ECL_RS07925* | -1.169218176 | nitrite reductase (NAD(P)H) large subunit |
| *ECL_RS26350* | -1.165680513 | hypothetical protein |
| *ECL_RS10805* | -1.1597747 | putative ABC transporter, periplasmic protein |
| *ECL_RS08735* | -1.158206734 | Lipase secretion C |
| *ECL_RS25290* | -1.155896605 | rhamnulokinase |
| *ECL_RS09475* | -1.155783611 | hypothetical protein |
| *ECL_RS13075* | -1.152302658 | guanine deaminase |
| *ECL_RS05475* | -1.150354752 | sulfonate/nitrate/taurine transport system ATP-binding protein |
| *ECL_RS01880* | -1.147657846 | Macrolide-specific ABC-type efflux carrier |
| *ECL_RS06800* | -1.145188462 | branched-chain amino acid transport system permease protein |
| *ECL_RS01675* | -1.143945557 | ribose ABC superfamily ATP binding component |
| *ECL_RS01745* | -1.137504181 | phosphonate metabolism transcriptional regulator PhnF |
| *ECL_RS16085* | -1.13413814 | major facilitator superfamily metabolite/H(+) symporter |
| *ECL_RS08940* | -1.131058407 | type VI secretion system protein VasJ |
| *ECL_RS03535* | -1.126806357 | secretion protein HlyD family protein |
| *ECL_RS13745* | -1.124303487 | general secretion pathway protein D |
| *ECL_RS11025* | -1.123687239 | NAD-dependent epimerase/dehydratase family protein |
| *ECL_RS19920* | -1.121978931 | short chain dehydrogenase |
| *ECL_RS10580* | -1.118274746 | phenylacetyl-CoA oxygenase, alpha subunit |
| *ECL_RS15310* | -1.116152449 | isochorismate synthase |
| *ECL_RS21450* | -1.105484849 | citrate carrier protein |
| *ECL_RS05090* | -1.104396184 | peptide/nickel transport system ATP-binding protein |
| *ECL_RS19865* | -1.102134606 | type I secretion system ATPase family protein |
| *ECL_RS10560* | -1.096489569 | phenylacetate-CoA oxygenase, NAD(P)H oxidoreductase component |
| *ECL_RS00270* | -1.093697134 | multidrug efflux transport outer membrane protein EefC |
| *ECL_RS16340* | -1.093109985 | flagellar biosynthetic protein FlhB |
| *ECL_RS09445* | -1.090099601 | peptide/nickel transport system ATP-binding protein |
| *ECL_RS02095* | -1.089588516 | hypothetical protein |
| *ECL_RS15295* | -1.088422787 | 2,3-dihydroxybenzoate-2,3-dehydrogenase |
| *ECL_RS11785* | -1.078746147 | citrate transporter |
| *ECL_RS04625* | -1.077511856 | iron-hydroxamate transporter permease subunit |
| *ECL_RS11715* | -1.072897105 | putative symporter |
| *ECL_RS18795* | -1.072866709 | hypothetical protein |
| *ECL_RS07475* | -1.072829066 | AraC family transcriptional regulator |
| *ECL_RS07930* | -1.072488551 | nitrate reductase catalytic subunit |
| *ECL_RS08805* | -1.06404024 | oxidoreductase domain-containing protein |
| *ECL_RS04450* | -1.06193963 | hypothetical protein |
| *ECL_RS19225* | -1.061727136 | anaerobic dimethyl sulfoxide reductase subunit C |
| *ECL_RS03395* | -1.06018898 | hypothetical protein |
| *ECL_RS10775* | -1.059640982 | allophanate hydrolase |
| *ECL_RS14735* | -1.058990097 | PTS system, fructose-specific II ABC component |
| *ECL_RS14255* | -1.053606844 | RND efflux system, outer membrane lipoprotein |
| *ECL_RS15630* | -1.053193282 | TonB-dependent siderophore receptor |
| *ECL_RS03265* | -1.051618167 | phage ornithine carbamoyltransferase 2, chain F |
| *ECL_RS03620* | -1.050786981 | putative 4-hydroxyphenylacetate permease |
| *ECL_RS08895* | -1.050229369 | OmpA/MotB domain-containing protein |
| *ECL_RS16115* | -1.043051884 | chemotaxis protein MotA |
| *ECL_RS09920* | -1.042227694 | branched-chain amino acid transport system permease protein |
| *ECL_RS03380* | -1.041767931 | tryptophanyl-tRNA synthetase II |
| *ECL_RS10210* | -1.041323344 | type I secretion membrane protein, ATP binding protein, protein export |
| *ECL_RS03410* | -1.035977977 | hypothetical protein |
| *ECL_RS11745* | -1.034191519 | putative hemin transport protein |
| *ECL_RS12865* | -1.020043174 | putative monooxygenase RutA |
| *ECL_RS23595* | -1.019717886 | triphosphoribosyl-dephospho-CoA synthase |
| *ECL_RS11555* | -1.015880059 | D-methionine transport system ATP-binding protein |
| *ECL_RS13055* | -1.013038968 | allantoinase |
| *ECL_RS03115* | -1.012627933 | dihydroorotase |
| *ECL_RS16155* | -1.007809904 | Flagellar P-ring protein 1, FlgI |
| *ECL_RS00045* | -1.006573353 | 2-keto-3-deoxygalactonate kinase |
| *ECL_RS02030* | -1.004934918 | pilus biogenesis protein PilL |
| *ECL_RS12525* | -1.004393363 | major facilitator transporter |
| *ECL_RS02055* | -1.003932402 | type IV secretion system protein |
| *ECL_RS22180* | -1.00272359 | two-component system, NarL family, sensor histidine kinase EvgS |
| *ECL_RS16175* | -1.002093154 | Flagellar basal body FlaE domain protein |
| *ECL_RS08935* | -1.001919525 | type VI secretion system protein ImpL |
| *ECL_RS16230* | -1.000966707 | methyl-accepting chemotaxis protein |
| *ECL_RS07780* | 1.00540878 | hypothetical protein |
| *ECL_RS05790* | 1.01199207 | cytochrome o ubiquinol oxidase subunit II |
| *ECL_RS25425* | 1.01572692 | putative phosphatase |
| *ECL_RS12235* | 1.04542817 | hypothetical protein |
| *ECL_RS14750* | 1.06591632 | succinyl-CoA synthetase, beta subunit |
| *ECL_RS20495* | 1.08542653 | N-acylglucosamine-6-phosphate 2-epimerase |
| *ECL_RS15635* | 1.09450992 | hypothetical protein |
| *ECL_RS05305* | 1.16066323 | conserved hypothetical protein |
| *ECL_RS05255* | 1.20962958 | conserved hypothetical protein |
| *ECL_RS14780* | 1.22810904 | succinate dehydrogenase cytochrome b-556 subunit |
| *ECL_RS27070* | 1.26088733 | hypothetical protein |
| *ECL_RS20500* | 1.26362221 | PTS system, glucose-like IIB subunint |
| *ECL_RS18085* | 1.26882193 | ascorbate-specific PTS system enzyme IIC |
| *ECL_RS27445* | 1.34946994 | hypothetical protein |
| *ECL_RS06235* | 1.45508169 | major type 1 subunit fimbrin (pilin) |
| *ECL_RS06315* | 1.4982609 | hypothetical protein |
| *ECL_RS14775* | 1.65216508 | succinate dehydrogenase hydrophobic membrane anchor protein |
| *ECL_RS01435* | 1.70843529 | maltose-inducible porin |
| *ECL_RS22930* | 1.88430746 | GCN5-related N-acetyltransferase |
| *ECL_RS01440* | 1.96312433 | maltose regulon periplasmic protein |
| *ECL_RS22190* | 2.15976109 | hypothetical protein |
| *ECL_RS20990* | 2.20505876 | hypothetical protein |
| *ECL_RS18095* | 2.54528001 | ascorbate-specific PTS system enzyme IIA |
| *ECL_RS11205* | 2.7755667 | hypothetical protein |
| *ECL_RS11195* | 3.29441343 | hypothetical protein |

^b^ Gene ID is cited from https://www.ncbi.nlm.nih.gov/datasets/taxonomy/716541/names/. Annotations and functional assignments are from *Enterobacter cloacae* subsp*. cloacae* ATCC 13047.

**Supplementary Table 3** Analysis of YjgB homologs in various bacterial species

| **Scientific Name** | **Per. Ident (%)** | **Accession No.** |
| --- | --- | --- |
| ***Acanthopleuribacter*** |  |  |
| [*A. pedis*](https://www.ncbi.nlm.nih.gov/Taxonomy/Browser/wwwtax.cgi?id=442870) | 51.78 | WP_207862319.1 |
| ***Acidobacteriaceae*** |  |  |
| [*A. bacterium*](https://www.ncbi.nlm.nih.gov/Taxonomy/Browser/wwwtax.cgi?id=2052142) | 49.25 | [HEY1964286.1](https://www.ncbi.nlm.nih.gov/protein/HEY1964286.1?report=genbank&log$=prottop&blast_rank=6&RID=K0PWJNZ5016) |
| ***Acidobacteriota*** |  |  |
| [*A. bacterium*](https://www.ncbi.nlm.nih.gov/Taxonomy/Browser/wwwtax.cgi?id=1978231) | 56.76 | [MDR3762635.1](https://www.ncbi.nlm.nih.gov/protein/MDR3762635.1?report=genbank&log$=prottop&blast_rank=9&RID=K0PWJNZ5016) |
| ***Acinetobacter*** |  |  |
| [*A. baretiae*](https://www.ncbi.nlm.nih.gov/Taxonomy/Browser/wwwtax.cgi?id=2605383) | 63.69 | [WP_196422525.1](https://www.ncbi.nlm.nih.gov/protein/WP_196422525.1?report=genbank&log$=prottop&blast_rank=11&RID=K0CP96ZG013) |
| [*A. baumannii*](https://www.ncbi.nlm.nih.gov/Taxonomy/Browser/wwwtax.cgi?id=470) | 62.54 | [MDC4409984.1](https://www.ncbi.nlm.nih.gov/protein/MDC4409984.1?report=genbank&log$=prottop&blast_rank=1&RID=K0NX35DA013) |
| [*A. calcoaceticus*](https://www.ncbi.nlm.nih.gov/Taxonomy/Browser/wwwtax.cgi?id=471) | 62.61 | [WNY31673.1](https://www.ncbi.nlm.nih.gov/protein/WNY31673.1?report=genbank&log$=prottop&blast_rank=13&RID=K0CP96ZG013) |
| [*A. courvalinii*](https://www.ncbi.nlm.nih.gov/Taxonomy/Browser/wwwtax.cgi?id=280147) | 62.24 | [WP_199949185.1](https://www.ncbi.nlm.nih.gov/protein/WP_199949185.1?report=genbank&log$=prottop&blast_rank=15&RID=K0CP96ZG013) |
| [*A. defluvii*](https://www.ncbi.nlm.nih.gov/Taxonomy/Browser/wwwtax.cgi?id=1871111) | 62.54 | [WP_065995386.1](https://www.ncbi.nlm.nih.gov/protein/WP_065995386.1?report=genbank&log$=prottop&blast_rank=19&RID=K0CP96ZG013) |
| [*A. haemolyticus*](https://www.ncbi.nlm.nih.gov/Taxonomy/Browser/wwwtax.cgi?id=29430) | 62.83 | [WP_125502503.1](https://www.ncbi.nlm.nih.gov/protein/WP_125502503.1?report=genbank&log$=prottop&blast_rank=20&RID=K0CP96ZG013) |
| [*A. indicus*](https://www.ncbi.nlm.nih.gov/Taxonomy/Browser/wwwtax.cgi?id=756892) | 64.01 | [WP_075167945.1](https://www.ncbi.nlm.nih.gov/protein/WP_075167945.1?report=genbank&log$=prottop&blast_rank=21&RID=K0CP96ZG013) |
| [*A. junii*](https://www.ncbi.nlm.nih.gov/Taxonomy/Browser/wwwtax.cgi?id=40215) | 62.83 | [WP_042892128.1](https://www.ncbi.nlm.nih.gov/protein/WP_042892128.1?report=genbank&log$=prottop&blast_rank=24&RID=K0CP96ZG013) |
| [*A. lactucae*](https://www.ncbi.nlm.nih.gov/Taxonomy/Browser/wwwtax.cgi?id=1785128) | 61.36 | [WP_125698576.1](https://www.ncbi.nlm.nih.gov/protein/WP_125698576.1?report=genbank&log$=prottop&blast_rank=33&RID=K0CP96ZG013) |
| [*A. lwoffii*](https://www.ncbi.nlm.nih.gov/Taxonomy/Browser/wwwtax.cgi?id=28090) | 63.50 | [WP_222112408.1](https://www.ncbi.nlm.nih.gov/protein/WP_222112408.1?report=genbank&log$=prottop&blast_rank=36&RID=K0CP96ZG013) |
| [*A. oleivorans*](https://www.ncbi.nlm.nih.gov/Taxonomy/Browser/wwwtax.cgi?id=1148157) | 62.24 | [WP_174764969.1](https://www.ncbi.nlm.nih.gov/protein/WP_174764969.1?report=genbank&log$=prottop&blast_rank=37&RID=K0CP96ZG013) |
| [*A. pecorum*](https://www.ncbi.nlm.nih.gov/Taxonomy/Browser/wwwtax.cgi?id=2762215) | 63.20 | [WP_191730834.1](https://www.ncbi.nlm.nih.gov/protein/WP_191730834.1?report=genbank&log$=prottop&blast_rank=38&RID=K0CP96ZG013) |
| [*A. pittii*](https://www.ncbi.nlm.nih.gov/Taxonomy/Browser/wwwtax.cgi?id=48296) | 62.54 | [WP_373883644.1](https://www.ncbi.nlm.nih.gov/protein/WP_373883644.1?report=genbank&log$=prottop&blast_rank=43&RID=K0CP96ZG013) |
| [*A. pollinis*](https://www.ncbi.nlm.nih.gov/Taxonomy/Browser/wwwtax.cgi?id=2605270) | 62.24 | [WP_195771708.1](https://www.ncbi.nlm.nih.gov/protein/WP_195771708.1?report=genbank&log$=prottop&blast_rank=46&RID=K0CP96ZG013) |
| [*A. rathckeae*](https://www.ncbi.nlm.nih.gov/Taxonomy/Browser/wwwtax.cgi?id=2605272) | 65.88 | [WP_195768210.1](https://www.ncbi.nlm.nih.gov/protein/WP_195768210.1?report=genbank&log$=prottop&blast_rank=48&RID=K0CP96ZG013) |
| [*A. Taxon 24*](https://www.ncbi.nlm.nih.gov/Taxonomy/Browser/wwwtax.cgi?id=2839056) | 63.50 | [WP_131289165.1](https://www.ncbi.nlm.nih.gov/protein/WP_131289165.1?report=genbank&log$=prottop&blast_rank=72&RID=K0CP96ZG013) |
| [*A. terrae*](https://www.ncbi.nlm.nih.gov/Taxonomy/Browser/wwwtax.cgi?id=2731247) | 63.20 | [WP_171543673.1](https://www.ncbi.nlm.nih.gov/protein/WP_171543673.1?report=genbank&log$=prottop&blast_rank=73&RID=K0CP96ZG013) |
| [*A. terrestris*](https://www.ncbi.nlm.nih.gov/Taxonomy/Browser/wwwtax.cgi?id=2529843) | 63.80 | [WP_131384673.1](https://www.ncbi.nlm.nih.gov/protein/WP_131384673.1?report=genbank&log$=prottop&blast_rank=76&RID=K0CP96ZG013) |
| [*A. towneri*](https://www.ncbi.nlm.nih.gov/Taxonomy/Browser/wwwtax.cgi?id=202956) | 63.42 | [WP_253107121.1](https://www.ncbi.nlm.nih.gov/protein/WP_253107121.1?report=genbank&log$=prottop&blast_rank=82&RID=K0CP96ZG013) |
| [*A. variabilis*](https://www.ncbi.nlm.nih.gov/Taxonomy/Browser/wwwtax.cgi?id=70346) | 63.20 | [MBO3660773.1](https://www.ncbi.nlm.nih.gov/protein/MBO3660773.1?report=genbank&log$=prottop&blast_rank=90&RID=K0CP96ZG013) |
| [*A. venetianus*](https://www.ncbi.nlm.nih.gov/Taxonomy/Browser/wwwtax.cgi?id=52133) | 75.67 | [QNH53065.1](https://www.ncbi.nlm.nih.gov/protein/QNH53065.1?report=genbank&log$=prottop&blast_rank=91&RID=K0CP96ZG013) |
| ***Aquipseudomonas*** |  |  |
| [*A. alcaligenes*](https://www.ncbi.nlm.nih.gov/Taxonomy/Browser/wwwtax.cgi?id=43263) | 51.54 | [WP_187804446.1](https://www.ncbi.nlm.nih.gov/protein/WP_187804446.1?report=genbank&log$=prottop&blast_rank=1&RID=K0TMZCRX013) |
| [*A. campi*](https://www.ncbi.nlm.nih.gov/Taxonomy/Browser/wwwtax.cgi?id=2731681) | 50.44 | [WP_173209564.1](https://www.ncbi.nlm.nih.gov/protein/WP_173209564.1?report=genbank&log$=prottop&blast_rank=1&RID=K0TPGXUH013) |
| ***Blastocatellia*** |  |  |
| [*B. bacterium*](https://www.ncbi.nlm.nih.gov/Taxonomy/Browser/wwwtax.cgi?id=2052146) | 53.87 | [HXU09525.1](https://www.ncbi.nlm.nih.gov/protein/HXU09525.1?report=genbank&log$=prottop&blast_rank=36&RID=K0PWJNZ5016) |
| ***Bryobacteraceae*** |  |  |
| [*B. bacterium*](https://www.ncbi.nlm.nih.gov/Taxonomy/Browser/wwwtax.cgi?id=2212468) | 50.77 | [HEY1756173.1](https://www.ncbi.nlm.nih.gov/protein/HEY1756173.1?report=genbank&log$=prottop&blast_rank=43&RID=K0PWJNZ5016) |
| ***Bryobacterales*** |  |  |
| [*B. bacterium*](https://www.ncbi.nlm.nih.gov/Taxonomy/Browser/wwwtax.cgi?id=2026791) | 49.85 | [MCU1294225.1](https://www.ncbi.nlm.nih.gov/protein/MCU1294225.1?report=genbank&log$=prottop&blast_rank=45&RID=K0PWJNZ5016) |
| ***Candidatus*** |  |  |
| [*C. Acidiferrales bacterium*](https://www.ncbi.nlm.nih.gov/Taxonomy/Browser/wwwtax.cgi?id=2952895) | 51.35 | [HXB59501.1](https://www.ncbi.nlm.nih.gov/protein/HXB59501.1?report=genbank&log$=prottop&blast_rank=46&RID=K0PWJNZ5016) |
| [*C. Pantoea deserta*](https://www.ncbi.nlm.nih.gov/Taxonomy/Browser/wwwtax.cgi?id=1869313) | 72.70 | [WP_123802314.1](https://www.ncbi.nlm.nih.gov/protein/WP_123802314.1?report=genbank&log$=prottop&blast_rank=1&RID=K0SMEWNA016) |
| [*C. Pantoea formicae*](https://www.ncbi.nlm.nih.gov/Taxonomy/Browser/wwwtax.cgi?id=2608355) | 75.07 | [WP_167134346.1](https://www.ncbi.nlm.nih.gov/protein/WP_167134346.1?report=genbank&log$=prottop&blast_rank=2&RID=K0SMEWNA016) |
| [*C. Pantoea soli*](https://www.ncbi.nlm.nih.gov/Taxonomy/Browser/wwwtax.cgi?id=3098669) | 76.26 | [WP_145891353.1](https://www.ncbi.nlm.nih.gov/protein/WP_145891353.1?report=genbank&log$=prottop&blast_rank=3&RID=K0SMEWNA016) |
| ***Cedecea*** |  |  |
| [*C. colo*](https://www.ncbi.nlm.nih.gov/Taxonomy/Browser/wwwtax.cgi?id=2552946) | 88.50 | [WP_167614615.1](https://www.ncbi.nlm.nih.gov/protein/WP_167614615.1?report=genbank&log$=prottop&blast_rank=6&RID=K0PXMHZ6013) |
| [*C. davisae*](https://www.ncbi.nlm.nih.gov/Taxonomy/Browser/wwwtax.cgi?id=158484) | 87.61 | [WP_016538309.1](https://www.ncbi.nlm.nih.gov/protein/WP_016538309.1?report=genbank&log$=prottop&blast_rank=14&RID=K0PXMHZ6013) |
| [*C. lapagei*](https://www.ncbi.nlm.nih.gov/Taxonomy/Browser/wwwtax.cgi?id=158823) | 87.32 | [WP_126354447.1](https://www.ncbi.nlm.nih.gov/protein/WP_126354447.1?report=genbank&log$=prottop&blast_rank=31&RID=K0PXMHZ6013) |
| [*C. lapagei*](https://www.ncbi.nlm.nih.gov/Taxonomy/Browser/wwwtax.cgi?id=158823) | 84.66 | [WP_213715489.1](https://www.ncbi.nlm.nih.gov/protein/WP_213715489.1?report=genbank&log$=prottop&blast_rank=32&RID=K0PXMHZ6013) |
| [*C. neteri*](https://www.ncbi.nlm.nih.gov/Taxonomy/Browser/wwwtax.cgi?id=158822) | 87.32 | [WP_061277840.1](https://www.ncbi.nlm.nih.gov/protein/WP_061277840.1?report=genbank&log$=prottop&blast_rank=38&RID=K0PXMHZ6013) |
| [*C. sulfonylureivorans*](https://www.ncbi.nlm.nih.gov/Taxonomy/Browser/wwwtax.cgi?id=3051154) | 87.02 | [WP_202301487.1](https://www.ncbi.nlm.nih.gov/protein/WP_202301487.1?report=genbank&log$=prottop&blast_rank=81&RID=K0PXMHZ6013) |
| ***Citrobacter*** |  |  |
| [*C. cronae*](https://www.ncbi.nlm.nih.gov/Taxonomy/Browser/wwwtax.cgi?id=1748967) | 94.40 | [WP_204525605.1](https://www.ncbi.nlm.nih.gov/protein/WP_204525605.1?report=genbank&log$=prottop&blast_rank=33&RID=K0PMXG8F013) |
| [*C. freundii*](https://www.ncbi.nlm.nih.gov/Taxonomy/Browser/wwwtax.cgi?id=546) | 94.99 | [WP_058842281.1](https://www.ncbi.nlm.nih.gov/protein/WP_058842281.1?report=genbank&log$=prottop&blast_rank=35&RID=K0PMXG8F013) |
| [*C. koseri*](https://www.ncbi.nlm.nih.gov/Taxonomy/Browser/wwwtax.cgi?id=545) | 93.81 | [WP_275376195.1](https://www.ncbi.nlm.nih.gov/protein/WP_275376195.1?report=genbank&log$=prottop&blast_rank=70&RID=K0PMXG8F013) |
| [*C. portucalensis*](https://www.ncbi.nlm.nih.gov/Taxonomy/Browser/wwwtax.cgi?id=1639133) | 95.28 | [WP_208744757.1](https://www.ncbi.nlm.nih.gov/protein/WP_208744757.1?report=genbank&log$=prottop&blast_rank=72&RID=K0PMXG8F013) |
| [*C. werkmanii*](https://www.ncbi.nlm.nih.gov/Taxonomy/Browser/wwwtax.cgi?id=67827) | 94.99 | [WP_200012620.1](https://www.ncbi.nlm.nih.gov/protein/WP_200012620.1?report=genbank&log$=prottop&blast_rank=90&RID=K0PMXG8F013) |
| [*C. youngae*](https://www.ncbi.nlm.nih.gov/Taxonomy/Browser/wwwtax.cgi?id=133448) | 94.40 | [WP_172742789.1](https://www.ncbi.nlm.nih.gov/protein/WP_172742789.1?report=genbank&log$=prottop&blast_rank=95&RID=K0PMXG8F013) |
| ***Ectopseudomonas*** |  |  |
| [*E. alcaliphila JAB1*](https://www.ncbi.nlm.nih.gov/Taxonomy/Browser/wwwtax.cgi?id=741155) | 50.30 | [APU31821.1](https://www.ncbi.nlm.nih.gov/protein/APU31821.1?report=genbank&log$=prottop&blast_rank=1&RID=K0TNNY5W013) |
| [*E. chengduensis*](https://www.ncbi.nlm.nih.gov/Taxonomy/Browser/wwwtax.cgi?id=489632) | 51.18 | [MDH1211441.1](https://www.ncbi.nlm.nih.gov/protein/MDH1211441.1?report=genbank&log$=prottop&blast_rank=1&RID=K0TTSW9M013) |
| [*E. guguanensis*](https://www.ncbi.nlm.nih.gov/Taxonomy/Browser/wwwtax.cgi?id=1198456) | 50.74 | [WP_024309543.1](https://www.ncbi.nlm.nih.gov/protein/WP_024309543.1?report=genbank&log$=prottop&blast_rank=1&RID=K0U1EX0K013) |
| [*E. hydrolytica*](https://www.ncbi.nlm.nih.gov/Taxonomy/Browser/wwwtax.cgi?id=2493633) | 50.15 | [WP_195882408.1](https://www.ncbi.nlm.nih.gov/protein/WP_195882408.1?report=genbank&log$=prottop&blast_rank=3&RID=K0U1XNHZ016) |
| ***Edaphobacter*** |  |  |
| [*E. paludis*](https://www.ncbi.nlm.nih.gov/Taxonomy/Browser/wwwtax.cgi?id=3035702) | 54.57 | [WP_348267144.1](https://www.ncbi.nlm.nih.gov/protein/WP_348267144.1?report=genbank&log$=prottop&blast_rank=56&RID=K0PWJNZ5016) |
| ***Enterobacter*** |  |  |
| [*E. asburiae*](https://www.ncbi.nlm.nih.gov/Taxonomy/Browser/wwwtax.cgi?id=61645) | 98.53 | [WP_337037906.1](https://www.ncbi.nlm.nih.gov/protein/WP_337037906.1?report=genbank&log$=prottop&blast_rank=18&RID=K0REHPUX013) |
| [*E. bugandensis*](https://www.ncbi.nlm.nih.gov/Taxonomy/Browser/wwwtax.cgi?id=881260) | 97.94 | [WP_248199503.1](https://www.ncbi.nlm.nih.gov/protein/WP_248199503.1?report=genbank&log$=prottop&blast_rank=33&RID=K0REHPUX013) |
| [*E. chengduensis*](https://www.ncbi.nlm.nih.gov/Taxonomy/Browser/wwwtax.cgi?id=2494701) | 97.64 | [WP_032642400.1](https://www.ncbi.nlm.nih.gov/protein/WP_032642400.1?report=genbank&log$=prottop&blast_rank=35&RID=K0REHPUX013) |
| [*E. chuandaensis*](https://www.ncbi.nlm.nih.gov/Taxonomy/Browser/wwwtax.cgi?id=2497875) | 98.23 | [WP_333016898.1](https://www.ncbi.nlm.nih.gov/protein/WP_333016898.1?report=genbank&log$=prottop&blast_rank=37&RID=K0REHPUX013) |
| [*E. hormaechei*](https://www.ncbi.nlm.nih.gov/Taxonomy/Browser/wwwtax.cgi?id=158836) | 98.23 | [WP_375602026.1](https://www.ncbi.nlm.nih.gov/protein/WP_375602026.1?report=genbank&log$=prottop&blast_rank=80&RID=K0REHPUX013) |
| [*E. huaxiensis*](https://www.ncbi.nlm.nih.gov/Taxonomy/Browser/wwwtax.cgi?id=2494702) | 98.53 | [WP_119936804.1](https://www.ncbi.nlm.nih.gov/protein/WP_119936804.1?report=genbank&log$=prottop&blast_rank=82&RID=K0REHPUX013) |
| [*E. mori*](https://www.ncbi.nlm.nih.gov/Taxonomy/Browser/wwwtax.cgi?id=539813) | 98.53 | [WP_126815471.1](https://www.ncbi.nlm.nih.gov/protein/WP_126815471.1?report=genbank&log$=prottop&blast_rank=84&RID=K0REHPUX013) |
| [*E. roggenkampii*](https://www.ncbi.nlm.nih.gov/Taxonomy/Browser/wwwtax.cgi?id=1812935) | 97.94 | [WP_248246140.1](https://www.ncbi.nlm.nih.gov/protein/WP_248246140.1?report=genbank&log$=prottop&blast_rank=88&RID=K0REHPUX013) |
| [*E. sichuanensis*](https://www.ncbi.nlm.nih.gov/Taxonomy/Browser/wwwtax.cgi?id=2071710) | 98.53 | [WP_333091574.1](https://www.ncbi.nlm.nih.gov/protein/WP_333091574.1?report=genbank&log$=prottop&blast_rank=91&RID=K0REHPUX013) |
| ***Escherichia*** |  |  |
| [*E. albertii*](https://www.ncbi.nlm.nih.gov/Taxonomy/Browser/wwwtax.cgi?id=208962) | 90.27 | [WP_273810133.1](https://www.ncbi.nlm.nih.gov/protein/WP_273810133.1?report=genbank&log$=prottop&blast_rank=1&RID=K0RJFEXW013) |
| [*E. coli*](https://www.ncbi.nlm.nih.gov/Taxonomy/Browser/wwwtax.cgi?id=562) | 98.53 | [MWO99173.1](https://www.ncbi.nlm.nih.gov/protein/MWO99173.1?report=genbank&log$=prottop&blast_rank=9&RID=K0RDXY9R013) |
| [*E. fergusonii*](https://www.ncbi.nlm.nih.gov/Taxonomy/Browser/wwwtax.cgi?id=564) | 91.45 | [WP_279284623.1](https://www.ncbi.nlm.nih.gov/protein/WP_279284623.1?report=genbank&log$=prottop&blast_rank=97&RID=K0RDXY9R013) |
| ***Granulicella*** |  |  |
| [*G. rosea*](https://www.ncbi.nlm.nih.gov/Taxonomy/Browser/wwwtax.cgi?id=474952) | 50.16 | [WP_089407621.1](https://www.ncbi.nlm.nih.gov/protein/WP_089407621.1?report=genbank&log$=prottop&blast_rank=58&RID=K0PWJNZ5016) |
| ***Klebsiella*** |  |  |
| [*K. electrica*](https://www.ncbi.nlm.nih.gov/Taxonomy/Browser/wwwtax.cgi?id=1259973) | 88.50 | [WP_131050796.1](https://www.ncbi.nlm.nih.gov/protein/WP_131050796.1?report=genbank&log$=prottop&blast_rank=15&RID=K0SA3M1W013) |
| [*K. grimontii*](https://www.ncbi.nlm.nih.gov/Taxonomy/Browser/wwwtax.cgi?id=2058152) | 88.50 | [WP_049088884.1](https://www.ncbi.nlm.nih.gov/protein/WP_049088884.1?report=genbank&log$=prottop&blast_rank=16&RID=K0SA3M1W013) |
| [*K. michiganensis*](https://www.ncbi.nlm.nih.gov/Taxonomy/Browser/wwwtax.cgi?id=1134687) | 88.50 | [HEJ8620393.1](https://www.ncbi.nlm.nih.gov/protein/HEJ8620393.1?report=genbank&log$=prottop&blast_rank=31&RID=K0SA3M1W013) |
| [*K. oxytoca*](https://www.ncbi.nlm.nih.gov/Taxonomy/Browser/wwwtax.cgi?id=571) | 88.79 | [HCB1847866.1](https://www.ncbi.nlm.nih.gov/protein/HCB1847866.1?report=genbank&log$=prottop&blast_rank=56&RID=K0SA3M1W013) |
| [*K. pasteurii*](https://www.ncbi.nlm.nih.gov/Taxonomy/Browser/wwwtax.cgi?id=2587529) | 87.91 | [WP_142468504.1](https://www.ncbi.nlm.nih.gov/protein/WP_142468504.1?report=genbank&log$=prottop&blast_rank=77&RID=K0SA3M1W013) |
| [*K. pneumoniae*](https://www.ncbi.nlm.nih.gov/Taxonomy/Browser/wwwtax.cgi?id=573) | 98.23 | [SSW79356.1](https://www.ncbi.nlm.nih.gov/protein/SSW79356.1?report=genbank&log$=prottop&blast_rank=81&RID=K0SA3M1W013) |
| [*K. quasipneumoniae*](https://www.ncbi.nlm.nih.gov/Taxonomy/Browser/wwwtax.cgi?id=1463165) | 88.20 | [WP_124074385.1](https://www.ncbi.nlm.nih.gov/protein/WP_124074385.1?report=genbank&log$=prottop&blast_rank=84&RID=K0SA3M1W013) |
| [*K. spallanzanii*](https://www.ncbi.nlm.nih.gov/Taxonomy/Browser/wwwtax.cgi?id=2587528) | 88.50 | [WP_142464499.1](https://www.ncbi.nlm.nih.gov/protein/WP_142464499.1?report=genbank&log$=prottop&blast_rank=94&RID=K0SA3M1W013) |
| [*K. variicola*](https://www.ncbi.nlm.nih.gov/Taxonomy/Browser/wwwtax.cgi?id=244366) | 87.91 | [WP_250376159.1](https://www.ncbi.nlm.nih.gov/protein/WP_250376159.1?report=genbank&log$=prottop&blast_rank=98&RID=K0SA3M1W013) |
| ***Kluyvera*** |  |  |
| [*K. ascorbata*](https://www.ncbi.nlm.nih.gov/Taxonomy/Browser/wwwtax.cgi?id=51288) | 89.09 | [WP_035892793.1](https://www.ncbi.nlm.nih.gov/protein/WP_035892793.1?report=genbank&log$=prottop&blast_rank=13&RID=K0RF3BVK013) |
| [*K. cryocrescens*](https://www.ncbi.nlm.nih.gov/Taxonomy/Browser/wwwtax.cgi?id=580) | 88.50 | [WP_313158720.1](https://www.ncbi.nlm.nih.gov/protein/WP_313158720.1?report=genbank&log$=prottop&blast_rank=50&RID=K0RF3BVK013) |
| [*K. georgiana*](https://www.ncbi.nlm.nih.gov/Taxonomy/Browser/wwwtax.cgi?id=73098) | 89.09 | [WP_065358342.1](https://www.ncbi.nlm.nih.gov/protein/WP_065358342.1?report=genbank&log$=prottop&blast_rank=68&RID=K0RF3BVK013) |
| [*K. intermedia*](https://www.ncbi.nlm.nih.gov/Taxonomy/Browser/wwwtax.cgi?id=61648) | 89.38 | [HAU8267321.1](https://www.ncbi.nlm.nih.gov/protein/HAU8267321.1?report=genbank&log$=prottop&blast_rank=71&RID=K0RF3BVK013) |
| [*K. sichuanensis*](https://www.ncbi.nlm.nih.gov/Taxonomy/Browser/wwwtax.cgi?id=2725494) | 88.79 | [WP_368300057.1](https://www.ncbi.nlm.nih.gov/protein/WP_368300057.1?report=genbank&log$=prottop&blast_rank=90&RID=K0RF3BVK013) |
| ***Kosakonia*** |  |  |
| [*K. arachidis*](https://www.ncbi.nlm.nih.gov/Taxonomy/Browser/wwwtax.cgi?id=551989) | 89.38 | [WP_090122138.1](https://www.ncbi.nlm.nih.gov/protein/WP_090122138.1?report=genbank&log$=prottop&blast_rank=22&RID=K0S5NBUD016) |
| [*K. cowanii*](https://www.ncbi.nlm.nih.gov/Taxonomy/Browser/wwwtax.cgi?id=208223) | 89.33 | [WP_312604852.1](https://www.ncbi.nlm.nih.gov/protein/WP_312604852.1?report=genbank&log$=prottop&blast_rank=30&RID=K0S5NBUD016) |
| [*K. oryzae*](https://www.ncbi.nlm.nih.gov/Taxonomy/Browser/wwwtax.cgi?id=497725) | 90.27 | [WP_064568631.1](https://www.ncbi.nlm.nih.gov/protein/WP_064568631.1?report=genbank&log$=prottop&blast_rank=52&RID=K0S5NBUD016) |
| [*K. oryziphila*](https://www.ncbi.nlm.nih.gov/Taxonomy/Browser/wwwtax.cgi?id=1005667) | 89.97 | [WP_090135615.1](https://www.ncbi.nlm.nih.gov/protein/WP_090135615.1?report=genbank&log$=prottop&blast_rank=55&RID=K0S5NBUD016) |
| [*K. pseudosacchari*](https://www.ncbi.nlm.nih.gov/Taxonomy/Browser/wwwtax.cgi?id=1646340) | 90.27 | [WP_193821690.1](https://www.ncbi.nlm.nih.gov/protein/WP_193821690.1?report=genbank&log$=prottop&blast_rank=58&RID=K0S5NBUD016) |
| [*K. radicincitans*](https://www.ncbi.nlm.nih.gov/Taxonomy/Browser/wwwtax.cgi?id=283686) | 90.27 | [WP_043955346.1](https://www.ncbi.nlm.nih.gov/protein/WP_043955346.1?report=genbank&log$=prottop&blast_rank=64&RID=K0S5NBUD016) |
| [*K. sacchari*](https://www.ncbi.nlm.nih.gov/Taxonomy/Browser/wwwtax.cgi?id=1158459) | 89.97 | [WP_289892207.1](https://www.ncbi.nlm.nih.gov/protein/WP_289892207.1?report=genbank&log$=prottop&blast_rank=74&RID=K0S5NBUD016) |
| ***Leclercia*** |  |  |
| [*L. adecarboxylata*](https://www.ncbi.nlm.nih.gov/Taxonomy/Browser/wwwtax.cgi?id=83655) | 96.17 | [WP_285129167.1](https://www.ncbi.nlm.nih.gov/protein/WP_285129167.1?report=genbank&log$=prottop&blast_rank=15&RID=K0SAUZ5A013) |
| [*L. pneumoniae*](https://www.ncbi.nlm.nih.gov/Taxonomy/Browser/wwwtax.cgi?id=2815358) | 98.82 | [WP_040073857.1](https://www.ncbi.nlm.nih.gov/protein/WP_040073857.1?report=genbank&log$=prottop&blast_rank=56&RID=K0SAUZ5A013) |
| [*L. tamurae*](https://www.ncbi.nlm.nih.gov/Taxonomy/Browser/wwwtax.cgi?id=2926467) | 94.99 | [WP_391488334.1](https://www.ncbi.nlm.nih.gov/protein/WP_391488334.1?report=genbank&log$=prottop&blast_rank=92&RID=K0SAUZ5A013) |
| ***Lelliottia*** |  |  |
| [*L. amnigena*](https://www.ncbi.nlm.nih.gov/Taxonomy/Browser/wwwtax.cgi?id=61646) | 97.64 | [WP_219347334.1](https://www.ncbi.nlm.nih.gov/protein/WP_219347334.1?report=genbank&log$=prottop&blast_rank=20&RID=K0SJZXDC013) |
| [*L. aquatilis*](https://www.ncbi.nlm.nih.gov/Taxonomy/Browser/wwwtax.cgi?id=2080838) | 97.35 | [WP_202674711.1](https://www.ncbi.nlm.nih.gov/protein/WP_202674711.1?report=genbank&log$=prottop&blast_rank=56&RID=K0SJZXDC013) |
| [*L. nimipressuralis*](https://www.ncbi.nlm.nih.gov/Taxonomy/Browser/wwwtax.cgi?id=69220) | 97.94 | [WP_134347392.1](https://www.ncbi.nlm.nih.gov/protein/WP_134347392.1?report=genbank&log$=prottop&blast_rank=61&RID=K0SJZXDC013) |
| [*L. wanjuensis*](https://www.ncbi.nlm.nih.gov/Taxonomy/Browser/wwwtax.cgi?id=3050585) | 97.35 | [WP_285157614.1](https://www.ncbi.nlm.nih.gov/protein/WP_285157614.1?report=genbank&log$=prottop&blast_rank=87&RID=K0SJZXDC013) |
| ***Mangrovibacter*** |  |  |
| [*M. phragmitis*](https://www.ncbi.nlm.nih.gov/Taxonomy/Browser/wwwtax.cgi?id=1691903) | 87.61 | [WP_369938615.1](https://www.ncbi.nlm.nih.gov/protein/WP_369938615.1?report=genbank&log$=prottop&blast_rank=7&RID=K0SM2UP1016) |
| [*M. yixingensis*](https://www.ncbi.nlm.nih.gov/Taxonomy/Browser/wwwtax.cgi?id=1529639) | 87.02 | [WP_226574577.1](https://www.ncbi.nlm.nih.gov/protein/WP_226574577.1?report=genbank&log$=prottop&blast_rank=33&RID=K0SM2UP1016) |
| ***Mannheimia*** |  |  |
| [*M. haemolytica*](https://www.ncbi.nlm.nih.gov/Taxonomy/Browser/wwwtax.cgi?id=75985) | 43.18 | [TRC07628.1](https://www.ncbi.nlm.nih.gov/protein/TRC07628.1?report=genbank&log$=prottop&blast_rank=35&RID=K0SKNSHA013) |
| ***Pantoea*** |  |  |
| [*P. alhagi*](https://www.ncbi.nlm.nih.gov/Taxonomy/Browser/wwwtax.cgi?id=1891675) | 75.96 | [ARJ43912.1](https://www.ncbi.nlm.nih.gov/protein/ARJ43912.1?report=genbank&log$=prottop&blast_rank=24&RID=K0SMEWNA016) |
| [*P. allii*](https://www.ncbi.nlm.nih.gov/Taxonomy/Browser/wwwtax.cgi?id=574096) | 75.00 | [WP_241568058.1](https://www.ncbi.nlm.nih.gov/protein/WP_241568058.1?report=genbank&log$=prottop&blast_rank=26&RID=K0SMEWNA016) |
| [*P. ananatis*](https://www.ncbi.nlm.nih.gov/Taxonomy/Browser/wwwtax.cgi?id=553) | 75.30 | [WP_264253217.1](https://www.ncbi.nlm.nih.gov/protein/WP_264253217.1?report=genbank&log$=prottop&blast_rank=27&RID=K0SMEWNA016) |
| [*P. cypripedii*](https://www.ncbi.nlm.nih.gov/Taxonomy/Browser/wwwtax.cgi?id=55209) | 73.59 | [WP_084880429.1](https://www.ncbi.nlm.nih.gov/protein/WP_084880429.1?report=genbank&log$=prottop&blast_rank=40&RID=K0SMEWNA016) |
| [*P. dispersa*](https://www.ncbi.nlm.nih.gov/Taxonomy/Browser/wwwtax.cgi?id=59814) | 76.26 | [WP_182688990.1](https://www.ncbi.nlm.nih.gov/protein/WP_182688990.1?report=genbank&log$=prottop&blast_rank=42&RID=K0SMEWNA016) |
| [*P. endophytica*](https://www.ncbi.nlm.nih.gov/Taxonomy/Browser/wwwtax.cgi?id=92488) | 73.89 | [WP_210080083.1](https://www.ncbi.nlm.nih.gov/protein/WP_210080083.1?report=genbank&log$=prottop&blast_rank=49&RID=K0SMEWNA016) |
| [*P. eucrina*](https://www.ncbi.nlm.nih.gov/Taxonomy/Browser/wwwtax.cgi?id=472693) | 75.37 | [WP_337026360.1](https://www.ncbi.nlm.nih.gov/protein/WP_337026360.1?report=genbank&log$=prottop&blast_rank=50&RID=K0SMEWNA016) |
| [*P. osteomyelitidis*](https://www.ncbi.nlm.nih.gov/Taxonomy/Browser/wwwtax.cgi?id=3230026) | 74.78 | [MFH8136021.1](https://www.ncbi.nlm.nih.gov/protein/MFH8136021.1?report=genbank&log$=prottop&blast_rank=52&RID=K0SMEWNA016) |
| [*P. phytobeneficialis*](https://www.ncbi.nlm.nih.gov/Taxonomy/Browser/wwwtax.cgi?id=2052056) | 74.78 | [WP_208725999.1](https://www.ncbi.nlm.nih.gov/protein/WP_208725999.1?report=genbank&log$=prottop&blast_rank=53&RID=K0SMEWNA016) |
| [*P. rodasii*](https://www.ncbi.nlm.nih.gov/Taxonomy/Browser/wwwtax.cgi?id=1076549) | 75.96 | [WP_100699794.1](https://www.ncbi.nlm.nih.gov/protein/WP_100699794.1?report=genbank&log$=prottop&blast_rank=54&RID=K0SMEWNA016) |
| [*P. rwandensis*](https://www.ncbi.nlm.nih.gov/Taxonomy/Browser/wwwtax.cgi?id=1076550) | 75.67 | [WP_084933956.1](https://www.ncbi.nlm.nih.gov/protein/WP_084933956.1?report=genbank&log$=prottop&blast_rank=57&RID=K0SMEWNA016) |
| [*P. septica*](https://www.ncbi.nlm.nih.gov/Taxonomy/Browser/wwwtax.cgi?id=472695) | 72.40 | [WP_312142688.1](https://www.ncbi.nlm.nih.gov/protein/WP_312142688.1?report=genbank&log$=prottop&blast_rank=58&RID=K0SMEWNA016) |
| [*P. stewartii*](https://www.ncbi.nlm.nih.gov/Taxonomy/Browser/wwwtax.cgi?id=66269) | 74.70 | [WP_054634074.1](https://www.ncbi.nlm.nih.gov/protein/WP_054634074.1?report=genbank&log$=prottop&blast_rank=90&RID=K0SMEWNA016) |
| [*P. wallisii*](https://www.ncbi.nlm.nih.gov/Taxonomy/Browser/wwwtax.cgi?id=1076551) | 74.78 | [WP_128601761.1](https://www.ncbi.nlm.nih.gov/protein/WP_128601761.1?report=genbank&log$=prottop&blast_rank=91&RID=K0SMEWNA016) |
| ***Pseudomonas*** |  |  |
| [*P. aeruginosa*](https://www.ncbi.nlm.nih.gov/Taxonomy/Browser/wwwtax.cgi?id=287) | 92.25 | [MBN0624069.1](https://www.ncbi.nlm.nih.gov/protein/MBN0624069.1?report=genbank&log$=prottop&blast_rank=1&RID=K0T5U75D016) |
| [*P. allokribbensis*](https://www.ncbi.nlm.nih.gov/Taxonomy/Browser/wwwtax.cgi?id=2774460) | 51.04 | [WP_192560640.1](https://www.ncbi.nlm.nih.gov/protein/WP_192560640.1?report=genbank&log$=prottop&blast_rank=1&RID=K0TB8AEJ013) |
| [*P. anguilliseptica*](https://www.ncbi.nlm.nih.gov/Taxonomy/Browser/wwwtax.cgi?id=53406) | 52.55 | [WP_269380036.1](https://www.ncbi.nlm.nih.gov/protein/WP_269380036.1?report=genbank&log$=prottop&blast_rank=2&RID=K0TBMDMT016) |
| [*P. benzenivorans*](https://www.ncbi.nlm.nih.gov/Taxonomy/Browser/wwwtax.cgi?id=556533) | 50.88 | [WP_255837157.1](https://www.ncbi.nlm.nih.gov/protein/WP_255837157.1?report=genbank&log$=prottop&blast_rank=1&RID=K0TBZ4FJ016) |
| [*P. berkeleyensis*](https://www.ncbi.nlm.nih.gov/Taxonomy/Browser/wwwtax.cgi?id=2726956) | 50.30 | [WP_179622548.1](https://www.ncbi.nlm.nih.gov/protein/WP_179622548.1?report=genbank&log$=prottop&blast_rank=1&RID=K0TCH627013) |
| [*P. entomophila*](https://www.ncbi.nlm.nih.gov/Taxonomy/Browser/wwwtax.cgi?id=312306) | 50.60 | [WP_011533867.1](https://www.ncbi.nlm.nih.gov/protein/WP_011533867.1?report=genbank&log$=prottop&blast_rank=1&RID=K0TUFK02016) |
| [*P. fitomaticsae*](https://www.ncbi.nlm.nih.gov/Taxonomy/Browser/wwwtax.cgi?id=2837969) | 50.45 | [WP_230737399.1](https://www.ncbi.nlm.nih.gov/protein/WP_230737399.1?report=genbank&log$=prottop&blast_rank=1&RID=K0TEJ7R9016) |
| [*P. fitomaticsae*](https://www.ncbi.nlm.nih.gov/Taxonomy/Browser/wwwtax.cgi?id=2837969) | 50.45 | [WP_230737399.1](https://www.ncbi.nlm.nih.gov/protein/WP_230737399.1?report=genbank&log$=prottop&blast_rank=1&RID=K0TVG964013) |
| [*P. fluorescens*](https://www.ncbi.nlm.nih.gov/Taxonomy/Browser/wwwtax.cgi?id=294) | 51.93 | [WP_064120983.1](https://www.ncbi.nlm.nih.gov/protein/WP_064120983.1?report=genbank&log$=prottop&blast_rank=1&RID=K0TF21E9013) |
| [*P. fluorescens*](https://www.ncbi.nlm.nih.gov/Taxonomy/Browser/wwwtax.cgi?id=294) | 51.93 | [WP_064120983.1](https://www.ncbi.nlm.nih.gov/protein/WP_064120983.1?report=genbank&log$=prottop&blast_rank=1&RID=K0TWAEXJ013) |
| [*P. gozinkensis*](https://www.ncbi.nlm.nih.gov/Taxonomy/Browser/wwwtax.cgi?id=2774461) | 50.45 | [WP_192564644.1](https://www.ncbi.nlm.nih.gov/protein/WP_192564644.1?report=genbank&log$=prottop&blast_rank=1&RID=K0U10AZN013) |
| [*P. indoloxydans*](https://www.ncbi.nlm.nih.gov/Taxonomy/Browser/wwwtax.cgi?id=404407) | 50.59 | [WP_108233862.1](https://www.ncbi.nlm.nih.gov/protein/WP_108233862.1?report=genbank&log$=prottop&blast_rank=1&RID=K0U2DTFU013) |
| [*P. izuensis*](https://www.ncbi.nlm.nih.gov/Taxonomy/Browser/wwwtax.cgi?id=2684212) | 50.44 | [WP_096510534.1](https://www.ncbi.nlm.nih.gov/protein/WP_096510534.1?report=genbank&log$=prottop&blast_rank=1&RID=K0U5C1XG013) |
| [*P. putida*](https://www.ncbi.nlm.nih.gov/Taxonomy/Browser/wwwtax.cgi?id=303) | 50.77 | [WP_110962296.1](https://www.ncbi.nlm.nih.gov/protein/WP_110962296.1?report=genbank&log$=prottop&blast_rank=1&RID=K0UANB3Z013) |
| ***Pyrinomonadaceae*** |  |  |
| [*P. bacterium*](https://www.ncbi.nlm.nih.gov/Taxonomy/Browser/wwwtax.cgi?id=2283092) | 54.44 | [MEO6590294.1](https://www.ncbi.nlm.nih.gov/protein/MEO6590294.1?report=genbank&log$=prottop&blast_rank=62&RID=K0PWJNZ5016) |
| ***Raoultella*** |  |  |
| [*R. ornithinolytica*](https://www.ncbi.nlm.nih.gov/Taxonomy/Browser/wwwtax.cgi?id=54291) | 89.88 | [WP_213895214.1](https://www.ncbi.nlm.nih.gov/protein/WP_213895214.1?report=genbank&log$=prottop&blast_rank=11&RID=K0UD3WSG016) |
| [*R. planticola*](https://www.ncbi.nlm.nih.gov/Taxonomy/Browser/wwwtax.cgi?id=575) | 89.09 | [WP_143718803.1](https://www.ncbi.nlm.nih.gov/protein/WP_143718803.1?report=genbank&log$=prottop&blast_rank=41&RID=K0UD3WSG016) |
| [*R. scottia*](https://www.ncbi.nlm.nih.gov/Taxonomy/Browser/wwwtax.cgi?id=3040937) | 87.32 | [WP_346430507.1](https://www.ncbi.nlm.nih.gov/protein/WP_346430507.1?report=genbank&log$=prottop&blast_rank=70&RID=K0UD3WSG016) |
| [*R. terrigena*](https://www.ncbi.nlm.nih.gov/Taxonomy/Browser/wwwtax.cgi?id=577) | 87.91 | [WP_123709820.1](https://www.ncbi.nlm.nih.gov/protein/WP_123709820.1?report=genbank&log$=prottop&blast_rank=83&RID=K0UD3WSG016) |
| ***Salmonella enterica subsp.*** |  |  |
| [*S. enterica*](https://www.ncbi.nlm.nih.gov/Taxonomy/Browser/wwwtax.cgi?id=59201) | 92.33 | [EAA7382666.1](https://www.ncbi.nlm.nih.gov/protein/EAA7382666.1?report=genbank&log$=prottop&blast_rank=70&RID=K0UDRR8U016) |
| [*S. enterica serovar Bovismorbificans*](https://www.ncbi.nlm.nih.gov/Taxonomy/Browser/wwwtax.cgi?id=58097) | 94.40 | [MBJ6034982.1](https://www.ncbi.nlm.nih.gov/protein/MBJ6034982.1?report=genbank&log$=prottop&blast_rank=74&RID=K0UDRR8U016) |
| [*S. enterica serovar Braenderup*](https://www.ncbi.nlm.nih.gov/Taxonomy/Browser/wwwtax.cgi?id=149391) | 90.86 | [EDW5363107.1](https://www.ncbi.nlm.nih.gov/protein/EDW5363107.1?report=genbank&log$=prottop&blast_rank=75&RID=K0UDRR8U016) |
| [*S. enterica serovar Choleraesuis*](https://www.ncbi.nlm.nih.gov/Taxonomy/Browser/wwwtax.cgi?id=119912) | 90.86 | [EBX8086574.1](https://www.ncbi.nlm.nih.gov/protein/EBX8086574.1?report=genbank&log$=prottop&blast_rank=76&RID=K0UDRR8U016) |
| [*S. enterica serovar Kentucky*](https://www.ncbi.nlm.nih.gov/Taxonomy/Browser/wwwtax.cgi?id=192955) | 90.56 | [EBW6256446.1](https://www.ncbi.nlm.nih.gov/protein/EBW6256446.1?report=genbank&log$=prottop&blast_rank=77&RID=K0UDRR8U016) |
| [*S. enterica serovar Koketime*](https://www.ncbi.nlm.nih.gov/Taxonomy/Browser/wwwtax.cgi?id=2564632) | 91.15 | [EAA7090671.1](https://www.ncbi.nlm.nih.gov/protein/EAA7090671.1?report=genbank&log$=prottop&blast_rank=78&RID=K0UDRR8U016) |
| [*S. enterica serovar Lisboa*](https://www.ncbi.nlm.nih.gov/Taxonomy/Browser/wwwtax.cgi?id=2564697) | 90.86 | [EAC0927610.1](https://www.ncbi.nlm.nih.gov/protein/EAC0927610.1?report=genbank&log$=prottop&blast_rank=79&RID=K0UDRR8U016) |
| [*S. enterica serovar Llandoff*](https://www.ncbi.nlm.nih.gov/Taxonomy/Browser/wwwtax.cgi?id=2564701) | 91.74 | [ECG4948149.1](https://www.ncbi.nlm.nih.gov/protein/ECG4948149.1?report=genbank&log$=prottop&blast_rank=80&RID=K0UDRR8U016) |
| [*S. enterica serovar Manhattan*](https://www.ncbi.nlm.nih.gov/Taxonomy/Browser/wwwtax.cgi?id=340189) | 90.86 | [EBY8073716.1](https://www.ncbi.nlm.nih.gov/protein/EBY8073716.1?report=genbank&log$=prottop&blast_rank=81&RID=K0UDRR8U016) |
| [*S. enterica serovar Oranienburg*](https://www.ncbi.nlm.nih.gov/Taxonomy/Browser/wwwtax.cgi?id=28147) | 92.04 | [EAU5127998.1](https://www.ncbi.nlm.nih.gov/protein/EAU5127998.1?report=genbank&log$=prottop&blast_rank=82&RID=K0UDRR8U016) |
| [*S. enterica serovar Rough O:k:1,5*](https://www.ncbi.nlm.nih.gov/Taxonomy/Browser/wwwtax.cgi?id=2724873) | 91.15 | [ECS6014657.1](https://www.ncbi.nlm.nih.gov/protein/ECS6014657.1?report=genbank&log$=prottop&blast_rank=84&RID=K0UDRR8U016) |
| [*S. enterica serovar Toulon*](https://www.ncbi.nlm.nih.gov/Taxonomy/Browser/wwwtax.cgi?id=149389) | 91.15 | [EBY0372655.1](https://www.ncbi.nlm.nih.gov/protein/EBY0372655.1?report=genbank&log$=prottop&blast_rank=85&RID=K0UDRR8U016) |
| [*S. houtenae*](https://www.ncbi.nlm.nih.gov/Taxonomy/Browser/wwwtax.cgi?id=59205) | 92.92 | [ECJ5920789.1](https://www.ncbi.nlm.nih.gov/protein/ECJ5920789.1?report=genbank&log$=prottop&blast_rank=86&RID=K0UDRR8U016) |
| ***Shigella*** |  |  |
| [*S. boydii*](https://www.ncbi.nlm.nih.gov/Taxonomy/Browser/wwwtax.cgi?id=621) | 90.86 | [WP_208926602.1](https://www.ncbi.nlm.nih.gov/protein/WP_208926602.1?report=genbank&log$=prottop&blast_rank=34&RID=K0UE6YT3016) |
| [*S. dysenteriae*](https://www.ncbi.nlm.nih.gov/Taxonomy/Browser/wwwtax.cgi?id=622) | 90.56 | [WP_005016196.1](https://www.ncbi.nlm.nih.gov/protein/WP_005016196.1?report=genbank&log$=prottop&blast_rank=39&RID=K0UE6YT3016) |
| [*S. flexneri*](https://www.ncbi.nlm.nih.gov/Taxonomy/Browser/wwwtax.cgi?id=766154) | 94.69 | [EIQ53054.1](https://www.ncbi.nlm.nih.gov/protein/EIQ53054.1?report=genbank&log$=prottop&blast_rank=72&RID=K0UE6YT3016) |
| [*S. sonnei*](https://www.ncbi.nlm.nih.gov/Taxonomy/Browser/wwwtax.cgi?id=624) | 91.15 | [EKM4385291.1](https://www.ncbi.nlm.nih.gov/protein/EKM4385291.1?report=genbank&log$=prottop&blast_rank=75&RID=K0UE6YT3016) |
| ***Terriglobales*** |  |  |
| [*T. bacterium*](https://www.ncbi.nlm.nih.gov/Taxonomy/Browser/wwwtax.cgi?id=2282142) | 55.08 | [HTE87363.1](https://www.ncbi.nlm.nih.gov/protein/HTE87363.1?report=genbank&log$=prottop&blast_rank=97&RID=K0PWJNZ5016) |
| ***Thermoanaerobaculales*** |  |  |
| [*T. bacterium*](https://www.ncbi.nlm.nih.gov/Taxonomy/Browser/wwwtax.cgi?id=2873827) | 49.55 | [MCJ7752926.1](https://www.ncbi.nlm.nih.gov/protein/MCJ7752926.1?report=genbank&log$=prottop&blast_rank=98&RID=K0PWJNZ5016) |
| ***Thermoanaerobaculia*** |  |  |
| [*T. bacterium*](https://www.ncbi.nlm.nih.gov/Taxonomy/Browser/wwwtax.cgi?id=2651171) | 52.62 | [HEY8131203.1](https://www.ncbi.nlm.nih.gov/protein/HEY8131203.1?report=genbank&log$=prottop&blast_rank=100&RID=K0PWJNZ5016) |
| ***Vibrio*** |  |  |
| [*V. agarivorans*](https://www.ncbi.nlm.nih.gov/Taxonomy/Browser/wwwtax.cgi?id=153622) | 44.88 | [WP_289961467.1](https://www.ncbi.nlm.nih.gov/protein/WP_289961467.1?report=genbank&log$=prottop&blast_rank=15&RID=K0V085G5016) |
| [*V. alginolyticus*](https://www.ncbi.nlm.nih.gov/Taxonomy/Browser/wwwtax.cgi?id=663) | 63.70 | [KPM94990.1](https://www.ncbi.nlm.nih.gov/protein/KPM94990.1?report=genbank&log$=prottop&blast_rank=17&RID=K0V085G5016) |
| [V. mexicanus](https://www.ncbi.nlm.nih.gov/Taxonomy/Browser/wwwtax.cgi?id=1004326) | 44.28 | [WP_047044320.1](https://www.ncbi.nlm.nih.gov/protein/WP_047044320.1?report=genbank&log$=prottop&blast_rank=46&RID=K0V085G5016) |
| [V. parahaemolyticus](https://www.ncbi.nlm.nih.gov/Taxonomy/Browser/wwwtax.cgi?id=670) | 90.31 | [MDF5581116.1](https://www.ncbi.nlm.nih.gov/protein/MDF5581116.1?report=genbank&log$=prottop&blast_rank=60&RID=K0V085G5016) |

**Supplementary Table 4** Analysis of ADH1 homologs in various fungal species

| **Scientific Name** | **Per. Ident (%)** | **Accession No.** |
| --- | --- | --- |
| ***Alternaria*** |  |  |
| [*A. alternata*](https://www.ncbi.nlm.nih.gov/Taxonomy/Browser/wwwtax.cgi?id=5599) | 73.47 | [XM_018527580.1](https://www.ncbi.nlm.nih.gov/nucleotide/XM_018527580.1?report=genbank&log$=nucltop&blast_rank=4&RID=J0JXT6P8013) |
| [*A. arborescens*](https://www.ncbi.nlm.nih.gov/Taxonomy/Browser/wwwtax.cgi?id=156630) | 72.12 | [XM_028651467.1](https://www.ncbi.nlm.nih.gov/nucleotide/XM_028651467.1?report=genbank&log$=nucltop&blast_rank=8&RID=J0JXT6P8013) |
| [*A. arbusti*](https://www.ncbi.nlm.nih.gov/Taxonomy/Browser/wwwtax.cgi?id=232088) | 72.24 | [XM_051441425.1](https://www.ncbi.nlm.nih.gov/nucleotide/XM_051441425.1?report=genbank&log$=nucltop&blast_rank=9&RID=J0JXT6P8013) |
| [*A. atra*](https://www.ncbi.nlm.nih.gov/Taxonomy/Browser/wwwtax.cgi?id=119953) | 69.21 | [XM_043313968.1](https://www.ncbi.nlm.nih.gov/nucleotide/XM_043313968.1?report=genbank&log$=nucltop&blast_rank=10&RID=J0JXT6P8013) |
| [*A. burnsii*](https://www.ncbi.nlm.nih.gov/Taxonomy/Browser/wwwtax.cgi?id=1187904) | 70.19 | [XM_038935686.1](https://www.ncbi.nlm.nih.gov/nucleotide/XM_038935686.1?report=genbank&log$=nucltop&blast_rank=16&RID=J0JXT6P8013) |
| [*A. conjuncta*](https://www.ncbi.nlm.nih.gov/Taxonomy/Browser/wwwtax.cgi?id=181017) | 70.29 | [XM_051466433.1](https://www.ncbi.nlm.nih.gov/nucleotide/XM_051466433.1?report=genbank&log$=nucltop&blast_rank=17&RID=J0JXT6P8013) |
| [*A. dauci*](https://www.ncbi.nlm.nih.gov/Taxonomy/Browser/wwwtax.cgi?id=48095) | 68.42 | [XM_069453143.1](https://www.ncbi.nlm.nih.gov/nucleotide/XM_069453143.1?report=genbank&log$=nucltop&blast_rank=19&RID=J0JXT6P8013) |
| [*A. ethzedia*](https://www.ncbi.nlm.nih.gov/Taxonomy/Browser/wwwtax.cgi?id=181014) | 70.70 | [XM_049377645.1](https://www.ncbi.nlm.nih.gov/nucleotide/XM_049377645.1?report=genbank&log$=nucltop&blast_rank=20&RID=J0JXT6P8013) |
| [*A. hordeiaustralica*](https://www.ncbi.nlm.nih.gov/Taxonomy/Browser/wwwtax.cgi?id=1187925) | 70.90 | [XM_049386807.1](https://www.ncbi.nlm.nih.gov/nucleotide/XM_049386807.1?report=genbank&log$=nucltop&blast_rank=21&RID=J0JXT6P8013) |
| [*A. incomplexa*](https://www.ncbi.nlm.nih.gov/Taxonomy/Browser/wwwtax.cgi?id=1187928) | 72.19 | [XM_051435047.1](https://www.ncbi.nlm.nih.gov/nucleotide/XM_051435047.1?report=genbank&log$=nucltop&blast_rank=22&RID=J0JXT6P8013) |
| [*A. infectoria*](https://www.ncbi.nlm.nih.gov/Taxonomy/Browser/wwwtax.cgi?id=45303) | 71.31 | [XM_051492477.1](https://www.ncbi.nlm.nih.gov/nucleotide/XM_051492477.1?report=genbank&log$=nucltop&blast_rank=23&RID=J0JXT6P8013) |
| [*A. metachromatica*](https://www.ncbi.nlm.nih.gov/Taxonomy/Browser/wwwtax.cgi?id=283354) | 69.82 | [XM_049328398.1](https://www.ncbi.nlm.nih.gov/nucleotide/XM_049328398.1?report=genbank&log$=nucltop&blast_rank=24&RID=J0JXT6P8013) |
| [*A. novae-zelandiae*](https://www.ncbi.nlm.nih.gov/Taxonomy/Browser/wwwtax.cgi?id=430562) | 70.49 | [XM_049403297.1](https://www.ncbi.nlm.nih.gov/nucleotide/XM_049403297.1?report=genbank&log$=nucltop&blast_rank=25&RID=J0JXT6P8013) |
| [*A. postmessia*](https://www.ncbi.nlm.nih.gov/Taxonomy/Browser/wwwtax.cgi?id=1187938) | 73.47 | [XM_051735833.1](https://www.ncbi.nlm.nih.gov/nucleotide/XM_051735833.1?report=genbank&log$=nucltop&blast_rank=27&RID=J0JXT6P8013) |
| [*A. rosae*](https://www.ncbi.nlm.nih.gov/Taxonomy/Browser/wwwtax.cgi?id=1187941) | 71.31 | [XM_046167028.1](https://www.ncbi.nlm.nih.gov/nucleotide/XM_046167028.1?report=genbank&log$=nucltop&blast_rank=28&RID=J0JXT6P8013) |
| [*A. triticimaculans*](https://www.ncbi.nlm.nih.gov/Taxonomy/Browser/wwwtax.cgi?id=297637) | 70.08 | [XM_049361720.1](https://www.ncbi.nlm.nih.gov/nucleotide/XM_049361720.1?report=genbank&log$=nucltop&blast_rank=31&RID=J0JXT6P8013) |
| [*A. ventricosa*](https://www.ncbi.nlm.nih.gov/Taxonomy/Browser/wwwtax.cgi?id=1187951) | 71.72 | [XM_049343054.1](https://www.ncbi.nlm.nih.gov/nucleotide/XM_049343054.1?report=genbank&log$=nucltop&blast_rank=32&RID=J0JXT6P8013) |
| [*A. viburni*](https://www.ncbi.nlm.nih.gov/Taxonomy/Browser/wwwtax.cgi?id=566460) | 69.88 | [XM_049350255.1](https://www.ncbi.nlm.nih.gov/nucleotide/XM_049350255.1?report=genbank&log$=nucltop&blast_rank=33&RID=J0JXT6P8013) |
| [***Armillaria***](https://www.ncbi.nlm.nih.gov/Taxonomy/Browser/wwwtax.cgi?id=47428) |  |  |
| [*A. ostoyae*](https://www.ncbi.nlm.nih.gov/Taxonomy/Browser/wwwtax.cgi?id=47428) | 83.33 | [SJL11947.1](https://www.ncbi.nlm.nih.gov/protein/SJL11947.1?report=genbank&log$=prottop&blast_rank=1&RID=K10G3FA5013) |
| [***Aspergillus***](https://www.ncbi.nlm.nih.gov/Taxonomy/Browser/wwwtax.cgi?id=1448322) |  |  |
| [*A. aculeatinus CBS 121060*](https://www.ncbi.nlm.nih.gov/Taxonomy/Browser/wwwtax.cgi?id=1448322) | 64.68 | [XM_025643949.1](https://www.ncbi.nlm.nih.gov/nucleotide/XM_025643949.1?report=genbank&log$=nucltop&blast_rank=1&RID=HZ9DJGAF013) |
| [*A. aculeatus ATCC 16872*](https://www.ncbi.nlm.nih.gov/Taxonomy/Browser/wwwtax.cgi?id=690307) | 66.31 | [XM_020199960.1](https://www.ncbi.nlm.nih.gov/nucleotide/XM_020199960.1?report=genbank&log$=nucltop&blast_rank=2&RID=HZ9DJGAF013) |
| [*A. affinis*](https://www.ncbi.nlm.nih.gov/Taxonomy/Browser/wwwtax.cgi?id=1070780) | 68.44 | [XM_053101180.1](https://www.ncbi.nlm.nih.gov/nucleotide/XM_053101180.1?report=genbank&log$=nucltop&blast_rank=3&RID=HZ9DJGAF013) |
| [*A. alliaceus*](https://www.ncbi.nlm.nih.gov/Taxonomy/Browser/wwwtax.cgi?id=209559) | 66.03 | [XM_032049090.1](https://www.ncbi.nlm.nih.gov/nucleotide/XM_032049090.1?report=genbank&log$=nucltop&blast_rank=4&RID=HZ9DJGAF013) |
| [*A. bombycis*](https://www.ncbi.nlm.nih.gov/Taxonomy/Browser/wwwtax.cgi?id=109264) | 68.81 | [XM_022539207.1](https://www.ncbi.nlm.nih.gov/nucleotide/XM_022539207.1?report=genbank&log$=nucltop&blast_rank=6&RID=HZ9DJGAF013) |
| [*A. brasiliensis CBS 101740*](https://www.ncbi.nlm.nih.gov/Taxonomy/Browser/wwwtax.cgi?id=767769) | 66.13 | [XM_067627838.1](https://www.ncbi.nlm.nih.gov/nucleotide/XM_067627838.1?report=genbank&log$=nucltop&blast_rank=7&RID=HZ9DJGAF013) |
| [*A. brunneoviolaceus*](https://www.ncbi.nlm.nih.gov/Taxonomy/Browser/wwwtax.cgi?id=1450534) | 64.83 | [XM_025581648.1](https://www.ncbi.nlm.nih.gov/nucleotide/XM_025581648.1?report=genbank&log$=nucltop&blast_rank=8&RID=HZ9DJGAF013) |
| [*A. caelatus*](https://www.ncbi.nlm.nih.gov/Taxonomy/Browser/wwwtax.cgi?id=61420) | 67.60 | [XM_032064456.1](https://www.ncbi.nlm.nih.gov/nucleotide/XM_032064456.1?report=genbank&log$=nucltop&blast_rank=9&RID=HZ9DJGAF013) |
| [*A. campestris IBT 28561*](https://www.ncbi.nlm.nih.gov/Taxonomy/Browser/wwwtax.cgi?id=1392248) | 65.76 | [XM_024836889.1](https://www.ncbi.nlm.nih.gov/nucleotide/XM_024836889.1?report=genbank&log$=nucltop&blast_rank=10&RID=HZ9DJGAF013) |
| [*A. candidus*](https://www.ncbi.nlm.nih.gov/Taxonomy/Browser/wwwtax.cgi?id=41067) | 65.11 | [XM_024819689.1](https://www.ncbi.nlm.nih.gov/nucleotide/XM_024819689.1?report=genbank&log$=nucltop&blast_rank=11&RID=HZ9DJGAF013) |
| [*A. chevalieri*](https://www.ncbi.nlm.nih.gov/Taxonomy/Browser/wwwtax.cgi?id=182096) | 56.20 | [XP_043139786.1](https://www.ncbi.nlm.nih.gov/protein/XP_043139786.1?report=genbank&log$=prottop&blast_rank=1&RID=K10N51XS016) |
| [*A. clavatus NRRL 1*](https://www.ncbi.nlm.nih.gov/Taxonomy/Browser/wwwtax.cgi?id=344612) | 67.72 | [XM_001272452.1](https://www.ncbi.nlm.nih.gov/nucleotide/XM_001272452.1?report=genbank&log$=nucltop&blast_rank=18&RID=HZ9DJGAF013) |
| [*A. costaricaensis CBS 115574*](https://www.ncbi.nlm.nih.gov/Taxonomy/Browser/wwwtax.cgi?id=1448317) | 68.23 | [XM_025683525.1](https://www.ncbi.nlm.nih.gov/nucleotide/XM_025683525.1?report=genbank&log$=nucltop&blast_rank=19&RID=HZ9DJGAF013) |
| [*A. eucalypticola CBS 122712*](https://www.ncbi.nlm.nih.gov/Taxonomy/Browser/wwwtax.cgi?id=1448314) | 67.86 | [XM_025536533.1](https://www.ncbi.nlm.nih.gov/nucleotide/XM_025536533.1?report=genbank&log$=nucltop&blast_rank=20&RID=HZ9DJGAF013) |
| [*A. felis*](https://www.ncbi.nlm.nih.gov/Taxonomy/Browser/wwwtax.cgi?id=1287682) | 56.16 | [KAF7159630.1](https://www.ncbi.nlm.nih.gov/protein/KAF7159630.1?report=genbank&log$=prottop&blast_rank=1&RID=K10PB6M9013) |
| [*A. fijiensis CBS 313.89*](https://www.ncbi.nlm.nih.gov/Taxonomy/Browser/wwwtax.cgi?id=1448319) | 65.04 | [XM_040941940.1](https://www.ncbi.nlm.nih.gov/nucleotide/XM_040941940.1?report=genbank&log$=nucltop&blast_rank=22&RID=HZ9DJGAF013) |
| [*A. fischeri NRRL 181*](https://www.ncbi.nlm.nih.gov/Taxonomy/Browser/wwwtax.cgi?id=331117) | 72.11 | [XM_001262737.1](https://www.ncbi.nlm.nih.gov/nucleotide/XM_001262737.1?report=genbank&log$=nucltop&blast_rank=24&RID=HZ9DJGAF013) |
| [*A. flavus*](https://www.ncbi.nlm.nih.gov/Taxonomy/Browser/wwwtax.cgi?id=5059) | 65.00 | [AAA32683.1](https://www.ncbi.nlm.nih.gov/protein/AAA32683.1?report=genbank&log$=prottop&blast_rank=1&RID=K10PTHKC016) |
| [*A. fumigatus*](https://www.ncbi.nlm.nih.gov/Taxonomy/Browser/wwwtax.cgi?id=746128) | 60.61 | [KAH3019802.1](https://www.ncbi.nlm.nih.gov/protein/KAH3019802.1?report=genbank&log$=prottop&blast_rank=1&RID=K10URJST013) |
| [*A. glaucus CBS 516.65*](https://www.ncbi.nlm.nih.gov/Taxonomy/Browser/wwwtax.cgi?id=1160497) | 70.10 | [XM_022544107.1](https://www.ncbi.nlm.nih.gov/nucleotide/XM_022544107.1?report=genbank&log$=nucltop&blast_rank=54&RID=HZ9DJGAF013) |
| [*A. heteromorphus CBS 117.55*](https://www.ncbi.nlm.nih.gov/Taxonomy/Browser/wwwtax.cgi?id=1448321) | 66.74 | [XM_025539641.1](https://www.ncbi.nlm.nih.gov/nucleotide/XM_025539641.1?report=genbank&log$=nucltop&blast_rank=55&RID=HZ9DJGAF013) |
| [*A. homomorphus CBS 101889*](https://www.ncbi.nlm.nih.gov/Taxonomy/Browser/wwwtax.cgi?id=1450537) | 64.89 | [XM_025692902.1](https://www.ncbi.nlm.nih.gov/nucleotide/XM_025692902.1?report=genbank&log$=nucltop&blast_rank=56&RID=HZ9DJGAF013) |
| [*A. ibericus CBS 121593*](https://www.ncbi.nlm.nih.gov/Taxonomy/Browser/wwwtax.cgi?id=1448316) | 66.95 | [XM_025723191.1](https://www.ncbi.nlm.nih.gov/nucleotide/XM_025723191.1?report=genbank&log$=nucltop&blast_rank=57&RID=HZ9DJGAF013) |
| [*A. japonicus CBS 114.51*](https://www.ncbi.nlm.nih.gov/Taxonomy/Browser/wwwtax.cgi?id=1448312) | 68.75 | [XM_025675387.1](https://www.ncbi.nlm.nih.gov/nucleotide/XM_025675387.1?report=genbank&log$=nucltop&blast_rank=58&RID=HZ9DJGAF013) |
| [*A. lentulus*](https://www.ncbi.nlm.nih.gov/Taxonomy/Browser/wwwtax.cgi?id=293939) | 70.62 | [XM_033559220.1](https://www.ncbi.nlm.nih.gov/nucleotide/XM_033559220.1?report=genbank&log$=nucltop&blast_rank=60&RID=HZ9DJGAF013) |
| [*A. luchuensis*](https://www.ncbi.nlm.nih.gov/Taxonomy/Browser/wwwtax.cgi?id=1069201) | 67.67 | [XM_041683458.1](https://www.ncbi.nlm.nih.gov/nucleotide/XM_041683458.1?report=genbank&log$=nucltop&blast_rank=61&RID=HZ9DJGAF013) |
| [*A. melleus*](https://www.ncbi.nlm.nih.gov/Taxonomy/Browser/wwwtax.cgi?id=138277) | 68.05 | [XM_046094029.1](https://www.ncbi.nlm.nih.gov/nucleotide/XM_046094029.1?report=genbank&log$=nucltop&blast_rank=64&RID=HZ9DJGAF013) |
| [*A. mulundensis*](https://www.ncbi.nlm.nih.gov/Taxonomy/Browser/wwwtax.cgi?id=1810919) | 71.58 | [XM_026747656.1](https://www.ncbi.nlm.nih.gov/nucleotide/XM_026747656.1?report=genbank&log$=nucltop&blast_rank=65&RID=HZ9DJGAF013) |
| [*A. neoniger CBS 115656*](https://www.ncbi.nlm.nih.gov/Taxonomy/Browser/wwwtax.cgi?id=1448310) | 67.86 | [XM_025619467.1](https://www.ncbi.nlm.nih.gov/nucleotide/XM_025619467.1?report=genbank&log$=nucltop&blast_rank=66&RID=HZ9DJGAF013) |
| [*A. niger*](https://www.ncbi.nlm.nih.gov/Taxonomy/Browser/wwwtax.cgi?id=5061) | 66.52 | [XM_001398345.3](https://www.ncbi.nlm.nih.gov/nucleotide/XM_001398345.3?report=genbank&log$=nucltop&blast_rank=67&RID=HZ9DJGAF013) |
| [*A. nomiae NRRL 13137*](https://www.ncbi.nlm.nih.gov/Taxonomy/Browser/wwwtax.cgi?id=1509407) | 67.72 | [XM_015545890.1](https://www.ncbi.nlm.nih.gov/nucleotide/XM_015545890.1?report=genbank&log$=nucltop&blast_rank=71&RID=HZ9DJGAF013) |
| [*A. novofumigatus IBT 16806*](https://www.ncbi.nlm.nih.gov/Taxonomy/Browser/wwwtax.cgi?id=1392255) | 71.65 | [XM_024825896.1](https://www.ncbi.nlm.nih.gov/nucleotide/XM_024825896.1?report=genbank&log$=nucltop&blast_rank=72&RID=HZ9DJGAF013) |
| [*A. ochraceoroseus IBT 24754*](https://www.ncbi.nlm.nih.gov/Taxonomy/Browser/wwwtax.cgi?id=1392256) | 66.67 | [XM_040893178.1](https://www.ncbi.nlm.nih.gov/nucleotide/XM_040893178.1?report=genbank&log$=nucltop&blast_rank=73&RID=HZ9DJGAF013) |
| [*A. oryzae*](https://www.ncbi.nlm.nih.gov/Taxonomy/Browser/wwwtax.cgi?id=5062) | 72.39 | [AB223300.1](https://www.ncbi.nlm.nih.gov/nucleotide/AB223300.1?report=genbank&log$=nucltop&blast_rank=77&RID=HZ9DJGAF013) |
| [*A. piperis CBS 112811*](https://www.ncbi.nlm.nih.gov/Taxonomy/Browser/wwwtax.cgi?id=1448313) | 68.05 | [XM_025656092.1](https://www.ncbi.nlm.nih.gov/nucleotide/XM_025656092.1?report=genbank&log$=nucltop&blast_rank=80&RID=HZ9DJGAF013) |
| [*A. pseudonomiae*](https://www.ncbi.nlm.nih.gov/Taxonomy/Browser/wwwtax.cgi?id=1506151) | 67.54 | [XM_032085090.1](https://www.ncbi.nlm.nih.gov/nucleotide/XM_032085090.1?report=genbank&log$=nucltop&blast_rank=81&RID=HZ9DJGAF013) |
| [*A. pseudotamarii*](https://www.ncbi.nlm.nih.gov/Taxonomy/Browser/wwwtax.cgi?id=132259) | 67.49 | [XM_032057334.1](https://www.ncbi.nlm.nih.gov/nucleotide/XM_032057334.1?report=genbank&log$=nucltop&blast_rank=82&RID=HZ9DJGAF013) |
| [*A. pseudoviridinutans*](https://www.ncbi.nlm.nih.gov/Taxonomy/Browser/wwwtax.cgi?id=1517512) | 71.13 | [XM_043306562.1](https://www.ncbi.nlm.nih.gov/nucleotide/XM_043306562.1?report=genbank&log$=nucltop&blast_rank=84&RID=HZ9DJGAF013) |
| [*A. ruber CBS 135680*](https://www.ncbi.nlm.nih.gov/Taxonomy/Browser/wwwtax.cgi?id=1388766) | 64.34 | [XM_040786751.1](https://www.ncbi.nlm.nih.gov/nucleotide/XM_040786751.1?report=genbank&log$=nucltop&blast_rank=85&RID=HZ9DJGAF013) |
| [*A. saccharolyticus JOP 1030-1*](https://www.ncbi.nlm.nih.gov/Taxonomy/Browser/wwwtax.cgi?id=1450539) | 66.76 | [XM_025577639.1](https://www.ncbi.nlm.nih.gov/nucleotide/XM_025577639.1?report=genbank&log$=nucltop&blast_rank=86&RID=HZ9DJGAF013) |
| [*A. sclerotioniger CBS 115572*](https://www.ncbi.nlm.nih.gov/Taxonomy/Browser/wwwtax.cgi?id=1450535) | 66.81 | [XM_025615754.1](https://www.ncbi.nlm.nih.gov/nucleotide/XM_025615754.1?report=genbank&log$=nucltop&blast_rank=87&RID=HZ9DJGAF013) |
| [*A. steynii IBT 23096*](https://www.ncbi.nlm.nih.gov/Taxonomy/Browser/wwwtax.cgi?id=1392250) | 67.01 | [XM_024852868.1](https://www.ncbi.nlm.nih.gov/nucleotide/XM_024852868.1?report=genbank&log$=nucltop&blast_rank=89&RID=HZ9DJGAF013) |
| [*A. terreus NIH2624*](https://www.ncbi.nlm.nih.gov/Taxonomy/Browser/wwwtax.cgi?id=341663) | 65.60 | [XM_001218028.1](https://www.ncbi.nlm.nih.gov/nucleotide/XM_001218028.1?report=genbank&log$=nucltop&blast_rank=90&RID=HZ9DJGAF013) |
| [*A. thermomutatus*](https://www.ncbi.nlm.nih.gov/Taxonomy/Browser/wwwtax.cgi?id=41047) | 72.68 | [XM_026760113.1](https://www.ncbi.nlm.nih.gov/nucleotide/XM_026760113.1?report=genbank&log$=nucltop&blast_rank=91&RID=HZ9DJGAF013) |
| [*A. tubingensis*](https://www.ncbi.nlm.nih.gov/Taxonomy/Browser/wwwtax.cgi?id=5068) | 68.42 | [XM_035505344.1](https://www.ncbi.nlm.nih.gov/nucleotide/XM_035505344.1?report=genbank&log$=nucltop&blast_rank=93&RID=HZ9DJGAF013) |
| [*A. udagawae*](https://www.ncbi.nlm.nih.gov/Taxonomy/Browser/wwwtax.cgi?id=91492) | 69.74 | [XM_043294497.1](https://www.ncbi.nlm.nih.gov/nucleotide/XM_043294497.1?report=genbank&log$=nucltop&blast_rank=95&RID=HZ9DJGAF013) |
| [*A. uvarum CBS 121591*](https://www.ncbi.nlm.nih.gov/Taxonomy/Browser/wwwtax.cgi?id=1448315) | 68.42 | [XM_025641633.1](https://www.ncbi.nlm.nih.gov/nucleotide/XM_025641633.1?report=genbank&log$=nucltop&blast_rank=96&RID=HZ9DJGAF013) |
| [*A. vadensis CBS 113365*](https://www.ncbi.nlm.nih.gov/Taxonomy/Browser/wwwtax.cgi?id=1448311) | 67.48 | [XM_025702496.1](https://www.ncbi.nlm.nih.gov/nucleotide/XM_025702496.1?report=genbank&log$=nucltop&blast_rank=97&RID=HZ9DJGAF013) |
| [*A. viridinutans*](https://www.ncbi.nlm.nih.gov/Taxonomy/Browser/wwwtax.cgi?id=75553) | 72.68 | [XM_043271573.1](https://www.ncbi.nlm.nih.gov/nucleotide/XM_043271573.1?report=genbank&log$=nucltop&blast_rank=98&RID=HZ9DJGAF013) |
| [*A. welwitschiae*](https://www.ncbi.nlm.nih.gov/Taxonomy/Browser/wwwtax.cgi?id=1341132) | 66.74 | [XM_026765629.1](https://www.ncbi.nlm.nih.gov/nucleotide/XM_026765629.1?report=genbank&log$=nucltop&blast_rank=99&RID=HZ9DJGAF013) |
| [*A. wentii DTO 134E9*](https://www.ncbi.nlm.nih.gov/Taxonomy/Browser/wwwtax.cgi?id=1073089) | 67.12 | [XM_040834116.1](https://www.ncbi.nlm.nih.gov/nucleotide/XM_040834116.1?report=genbank&log$=nucltop&blast_rank=100&RID=HZ9DJGAF013) |
| ***Aureobasidium*** |  |  |
| [*A. melanogenum*](https://www.ncbi.nlm.nih.gov/Taxonomy/Browser/wwwtax.cgi?id=46634) | 74.71 | [KAG9515563.1](https://www.ncbi.nlm.nih.gov/protein/KAG9515563.1?report=genbank&log$=prottop&blast_rank=1&RID=K1102D8R013) |
| [*A. namibiae CBS 147.97*](https://www.ncbi.nlm.nih.gov/Taxonomy/Browser/wwwtax.cgi?id=1043004) | 67.55 | [XM_013572214.1](https://www.ncbi.nlm.nih.gov/nucleotide/XM_013572214.1?report=genbank&log$=nucltop&blast_rank=6&RID=J0JZ1CYE016) |
| [*A. pullulans*](https://www.ncbi.nlm.nih.gov/Taxonomy/Browser/wwwtax.cgi?id=5580) | 69.55 | [KAK6007626.1](https://www.ncbi.nlm.nih.gov/protein/KAK6007626.1?report=genbank&log$=prottop&blast_rank=52&RID=K1102D8R013) |
| [*A. subglaciale EXF-2481*](https://www.ncbi.nlm.nih.gov/Taxonomy/Browser/wwwtax.cgi?id=1043005) | 68.53 | [XM_013493840.1](https://www.ncbi.nlm.nih.gov/nucleotide/XM_013493840.1?report=genbank&log$=nucltop&blast_rank=9&RID=J0JZ1CYE016) |
| [***Beauveria***](https://www.ncbi.nlm.nih.gov/Taxonomy/Browser/wwwtax.cgi?id=655819) |  |  |
| [*B. bassiana ARSEF 2860*](https://www.ncbi.nlm.nih.gov/Taxonomy/Browser/wwwtax.cgi?id=655819) | 72.03 | [XM_008601263.1](https://www.ncbi.nlm.nih.gov/nucleotide/XM_008601263.1?report=genbank&log$=nucltop&blast_rank=1&RID=J0K36XD7013) |
| [***Blastomyces***](https://www.ncbi.nlm.nih.gov/Taxonomy/Browser/wwwtax.cgi?id=559298) |  |  |
| [*B. dermatitidis ER-3*](https://www.ncbi.nlm.nih.gov/Taxonomy/Browser/wwwtax.cgi?id=559297) | 67.42 | [XM_045417585.1](https://www.ncbi.nlm.nih.gov/nucleotide/XM_045417585.1?report=genbank&log$=nucltop&blast_rank=2&RID=J0K9TTAE013) |
| [*B. gilchristii SLH14081*](https://www.ncbi.nlm.nih.gov/Taxonomy/Browser/wwwtax.cgi?id=559298) | 67.42 | [XM_031721431.1](https://www.ncbi.nlm.nih.gov/nucleotide/XM_031721431.1?report=genbank&log$=nucltop&blast_rank=3&RID=J0K9TTAE013) |
| [**Botrytis**](https://www.ncbi.nlm.nih.gov/Taxonomy/Browser/wwwtax.cgi?id=139641) |  |  |
| [*B. byssoidea*](https://www.ncbi.nlm.nih.gov/Taxonomy/Browser/wwwtax.cgi?id=139641) | 68.72 | [XM_038880450.1](https://www.ncbi.nlm.nih.gov/nucleotide/XM_038880450.1?report=genbank&log$=nucltop&blast_rank=1&RID=J0KA4YVX016) |
| [*B. cinerea*](https://www.ncbi.nlm.nih.gov/Taxonomy/Browser/wwwtax.cgi?id=40559) | 87.18 | [XP_001554746.1](https://www.ncbi.nlm.nih.gov/protein/XP_001554746.1?report=genbank&log$=prottop&blast_rank=22&RID=K111M8WX013) |
| [*B. deweyae*](https://www.ncbi.nlm.nih.gov/Taxonomy/Browser/wwwtax.cgi?id=2478750) | 68.12 | [XM_038959488.1](https://www.ncbi.nlm.nih.gov/nucleotide/XM_038959488.1?report=genbank&log$=nucltop&blast_rank=14&RID=J0KA4YVX016) |
| [*B. fragariae*](https://www.ncbi.nlm.nih.gov/Taxonomy/Browser/wwwtax.cgi?id=1964551) | 66.67 | [XM_037339972.1](https://www.ncbi.nlm.nih.gov/nucleotide/XM_037339972.1?report=genbank&log$=nucltop&blast_rank=16&RID=J0KA4YVX016) |
| [*B. porri*](https://www.ncbi.nlm.nih.gov/Taxonomy/Browser/wwwtax.cgi?id=87229) | 65.82 | [XM_038920178.1](https://www.ncbi.nlm.nih.gov/nucleotide/XM_038920178.1?report=genbank&log$=nucltop&blast_rank=17&RID=J0KA4YVX016) |
| [*B. sinoallii*](https://www.ncbi.nlm.nih.gov/Taxonomy/Browser/wwwtax.cgi?id=1463999) | 67.15 | [XM_038897345.1](https://www.ncbi.nlm.nih.gov/nucleotide/XM_038897345.1?report=genbank&log$=nucltop&blast_rank=18&RID=J0KA4YVX016) |
| [***Candida***](https://www.ncbi.nlm.nih.gov/Taxonomy/Browser/wwwtax.cgi?id=5476) |  |  |
| *C. africana* | 75.57 | [KAG8204651.1](https://www.ncbi.nlm.nih.gov/protein/KAG8204651.1?report=genbank&log$=prottop&blast_rank=1&RID=K11AF1FB016) |
| [*C. albicans*](https://www.ncbi.nlm.nih.gov/Taxonomy/Browser/wwwtax.cgi?id=5476) | 77.01 | [XP_717649.1](https://www.ncbi.nlm.nih.gov/protein/XP_717649.1?report=genbank&log$=prottop&blast_rank=18&RID=K11AF1FB016) |
| *C. dubliniensis CD36* | 77.30 | [XP_002417431.1](https://www.ncbi.nlm.nih.gov/protein/XP_002417431.1?report=genbank&log$=prottop&blast_rank=23&RID=K11AF1FB016) |
| *C. jiufengensis* | 66.57 | [XP_051622040.1](https://www.ncbi.nlm.nih.gov/protein/XP_051622040.1?report=genbank&log$=prottop&blast_rank=26&RID=K11AF1FB016) |
| *C. maltosa* | 76.37 | [ADM08008.1](https://www.ncbi.nlm.nih.gov/protein/ADM08008.1?report=genbank&log$=prottop&blast_rank=28&RID=K11AF1FB016) |
| *C. margitis* | 74.28 | [XP_051673195.1](https://www.ncbi.nlm.nih.gov/protein/XP_051673195.1?report=genbank&log$=prottop&blast_rank=34&RID=K11AF1FB016) |
| *C. metapsilosis* | 73.41 | [XP_067549409.1](https://www.ncbi.nlm.nih.gov/protein/XP_067549409.1?report=genbank&log$=prottop&blast_rank=38&RID=K11AF1FB016) |
| *C. orthopsilosis Co 90-125* | 74.86 | [XP_003870523.1](https://www.ncbi.nlm.nih.gov/protein/XP_003870523.1?report=genbank&log$=prottop&blast_rank=43&RID=K11AF1FB016) |
| *C. oxycetoniae* | 74.64 | [XP_049180475.1](https://www.ncbi.nlm.nih.gov/protein/XP_049180475.1?report=genbank&log$=prottop&blast_rank=48&RID=K11AF1FB016) |
| [*C. parapsilosis*](https://www.ncbi.nlm.nih.gov/Taxonomy/Browser/wwwtax.cgi?id=5480) | 75.14 | [XP_036667197.1](https://www.ncbi.nlm.nih.gov/protein/XP_036667197.1?report=genbank&log$=prottop&blast_rank=51&RID=K11AF1FB016) |
| *C. pseudojiufengensis* | 65.71 | [XP_051615152.1](https://www.ncbi.nlm.nih.gov/protein/XP_051615152.1?report=genbank&log$=prottop&blast_rank=61&RID=K11AF1FB016) |
| *C. theae* | 73.12 | [XP_051610183.1](https://www.ncbi.nlm.nih.gov/protein/XP_051610183.1?report=genbank&log$=prottop&blast_rank=63&RID=K11AF1FB016) |
| [*C. tropicalis*](https://www.ncbi.nlm.nih.gov/Taxonomy/Browser/wwwtax.cgi?id=5482) | 77.53 | [ACX81423.1](https://www.ncbi.nlm.nih.gov/protein/ACX81423.1?report=genbank&log$=prottop&blast_rank=70&RID=K11AF1FB016) |
| *C. verbasci* | 68.88 | [CAI5759786.1](https://www.ncbi.nlm.nih.gov/protein/CAI5759786.1?report=genbank&log$=prottop&blast_rank=85&RID=K11AF1FB016) |
| *C. viswanathi* | 76.15 | [RCK65220.1](https://www.ncbi.nlm.nih.gov/protein/RCK65220.1?report=genbank&log$=prottop&blast_rank=91&RID=K11AF1FB016) |
| [***Cladosporium***](https://www.ncbi.nlm.nih.gov/Taxonomy/Browser/wwwtax.cgi?id=1052096) |  |  |
| [*C. halotolerans*](https://www.ncbi.nlm.nih.gov/Taxonomy/Browser/wwwtax.cgi?id=1052096) | 67.84 | [XM_069371929.1](https://www.ncbi.nlm.nih.gov/nucleotide/XM_069371929.1?report=genbank&log$=nucltop&blast_rank=2&RID=J0KRU6K2013) |
| ***Colletotrichum*** |  |  |
| [*C. siamense*](https://www.ncbi.nlm.nih.gov/Taxonomy/Browser/wwwtax.cgi?id=690259) | 66.87 | [XM_036634757.1](https://www.ncbi.nlm.nih.gov/nucleotide/XM_036634757.1?report=genbank&log$=nucltop&blast_rank=2&RID=J0KSM9XH013) |
| [***Cryptococcus***](https://www.ncbi.nlm.nih.gov/Taxonomy/Browser/wwwtax.cgi?id=1295533) |  |  |
| [*C. amylolentus CBS 6039*](https://www.ncbi.nlm.nih.gov/Taxonomy/Browser/wwwtax.cgi?id=1295533) | 74.80 | [XM_019137944.1](https://www.ncbi.nlm.nih.gov/nucleotide/XM_019137944.1?report=genbank&log$=nucltop&blast_rank=1&RID=HZA3G97H013) |
| [*C. bacillisporus CA1280*](https://www.ncbi.nlm.nih.gov/Taxonomy/Browser/wwwtax.cgi?id=1296109) | 74.03 | [XP_066608648.1](https://www.ncbi.nlm.nih.gov/protein/XP_066608648.1?report=genbank&log$=prottop&blast_rank=5&RID=K11ZAS8V016) |
| [*C. decagattii*](https://www.ncbi.nlm.nih.gov/Taxonomy/Browser/wwwtax.cgi?id=1859122) | 74.03 | [XP_064720546.1](https://www.ncbi.nlm.nih.gov/protein/XP_064720546.1?report=genbank&log$=prottop&blast_rank=8&RID=K11ZAS8V016) |
| [*C. gattii*](https://www.ncbi.nlm.nih.gov/Taxonomy/Browser/wwwtax.cgi?id=37769) | 74.19 | [AEI90603.1](https://www.ncbi.nlm.nih.gov/protein/AEI90603.1?report=genbank&log$=prottop&blast_rank=27&RID=K11ZAS8V016) |
| [*C. neoformans*](https://www.ncbi.nlm.nih.gov/Taxonomy/Browser/wwwtax.cgi?id=5207) | 76.56 | [ABB48493.1](https://www.ncbi.nlm.nih.gov/protein/ABB48493.1?report=genbank&log$=prottop&blast_rank=46&RID=K11ZAS8V016) |
| [*C. neoformans var. grubii H99*](https://www.ncbi.nlm.nih.gov/Taxonomy/Browser/wwwtax.cgi?id=235443) | 76.56 | [XP_012050990.1](https://www.ncbi.nlm.nih.gov/protein/XP_012050990.1?report=genbank&log$=prottop&blast_rank=75&RID=K11ZAS8V016) |
| [*C. tetragattii IND107*](https://www.ncbi.nlm.nih.gov/Taxonomy/Browser/wwwtax.cgi?id=1296105) | 67.16 | [XM_066757952.1](https://www.ncbi.nlm.nih.gov/nucleotide/XM_066757952.1?report=genbank&log$=nucltop&blast_rank=40&RID=J0H9MTPT016) |
| [*C. wingfieldii*](https://www.ncbi.nlm.nih.gov/Taxonomy/Browser/wwwtax.cgi?id=5619) | 72.3 | [XP_019030234.1](https://www.ncbi.nlm.nih.gov/protein/XP_019030234.1?report=genbank&log$=prottop&blast_rank=99&RID=K11ZAS8V016) |
| [***Fusarium***](https://www.ncbi.nlm.nih.gov/Taxonomy/Browser/wwwtax.cgi?id=5515) |  |  |
| [*F. acutatum*](https://www.ncbi.nlm.nih.gov/Taxonomy/Browser/wwwtax.cgi?id=5515) | 55.62 | [KAF4440124.1](https://www.ncbi.nlm.nih.gov/protein/KAF4440124.1?report=genbank&log$=prottop&blast_rank=1&RID=K120TSKZ013) |
| *F. agapanthi* | 57.59 | [KAF4500151.1](https://www.ncbi.nlm.nih.gov/protein/KAF4500151.1?report=genbank&log$=prottop&blast_rank=2&RID=K120TSKZ013) |
| *F. albosuccineum* | 57.47 | [KAF4466056.1](https://www.ncbi.nlm.nih.gov/protein/KAF4466056.1?report=genbank&log$=prottop&blast_rank=4&RID=K120TSKZ013) |
| [*F. avenaceum*](https://www.ncbi.nlm.nih.gov/Taxonomy/Browser/wwwtax.cgi?id=40199) | 68.95 | [KAG5659319.1](https://www.ncbi.nlm.nih.gov/protein/KAG5659319.1?report=genbank&log$=prottop&blast_rank=9&RID=K120TSKZ013) |
| [*F. coffeatum*](https://www.ncbi.nlm.nih.gov/Taxonomy/Browser/wwwtax.cgi?id=231269) | 68.60 | [XM_031163674.1](https://www.ncbi.nlm.nih.gov/nucleotide/XM_031163674.1?report=genbank&log$=nucltop&blast_rank=6&RID=J0HG56T0016) |
| [*F. culmorum*](https://www.ncbi.nlm.nih.gov/Taxonomy/Browser/wwwtax.cgi?id=5516) | 66.78 | [QPC62520.1](https://www.ncbi.nlm.nih.gov/protein/QPC62520.1?report=genbank&log$=prottop&blast_rank=22&RID=K120TSKZ013) |
| [*F. falciforme*](https://www.ncbi.nlm.nih.gov/Taxonomy/Browser/wwwtax.cgi?id=195108) | 67.98 | [XM_053154696.1](https://www.ncbi.nlm.nih.gov/nucleotide/XM_053154696.1?report=genbank&log$=nucltop&blast_rank=11&RID=J1040F1H013) |
| [*F. flagelliforme*](https://www.ncbi.nlm.nih.gov/Taxonomy/Browser/wwwtax.cgi?id=2675880) | 68.65 | [XM_046129499.1](https://www.ncbi.nlm.nih.gov/nucleotide/XM_046129499.1?report=genbank&log$=nucltop&blast_rank=17&RID=J1040F1H013) |
| [*F. fujikuroi*](https://www.ncbi.nlm.nih.gov/Taxonomy/Browser/wwwtax.cgi?id=5127) | 67.63 | [SCN69238.1](https://www.ncbi.nlm.nih.gov/protein/SCN69238.1?report=genbank&log$=prottop&blast_rank=28&RID=K120TSKZ013) |
| [*F. graminearum*](https://www.ncbi.nlm.nih.gov/Taxonomy/Browser/wwwtax.cgi?id=5518) | 69.19 | WXC56404.1 |
| [*F. keratoplasticum*](https://www.ncbi.nlm.nih.gov/Taxonomy/Browser/wwwtax.cgi?id=1328300) | 67.77 | [XM_053059312.1](https://www.ncbi.nlm.nih.gov/nucleotide/XM_053059312.1?report=genbank&log$=nucltop&blast_rank=38&RID=J1040F1H013) |
| [*F. mangiferae*](https://www.ncbi.nlm.nih.gov/Taxonomy/Browser/wwwtax.cgi?id=192010) | 67.84 | [XM_041823898.1](https://www.ncbi.nlm.nih.gov/nucleotide/XM_041823898.1?report=genbank&log$=nucltop&blast_rank=39&RID=J1040F1H013) |
| [*F. musae*](https://www.ncbi.nlm.nih.gov/Taxonomy/Browser/wwwtax.cgi?id=1042133) | 69.29 | [XM_044827522.1](https://www.ncbi.nlm.nih.gov/nucleotide/XM_044827522.1?report=genbank&log$=nucltop&blast_rank=43&RID=J1040F1H013) |
| [*F. odoratissimum NRRL 54006*](https://www.ncbi.nlm.nih.gov/Taxonomy/Browser/wwwtax.cgi?id=1089451) | 68.94 | [XM_031207368.1](https://www.ncbi.nlm.nih.gov/nucleotide/XM_031207368.1?report=genbank&log$=nucltop&blast_rank=45&RID=J1040F1H013) |
| [*F. oxysporum*](https://www.ncbi.nlm.nih.gov/Taxonomy/Browser/wwwtax.cgi?id=5507) | 68.53 | [KAI7758511.1](https://www.ncbi.nlm.nih.gov/protein/KAI7758511.1?report=genbank&log$=prottop&blast_rank=48&RID=K120TSKZ013) |
| [*F. poae*](https://www.ncbi.nlm.nih.gov/Taxonomy/Browser/wwwtax.cgi?id=36050) | 69.41 | [XM_044854009.1](https://www.ncbi.nlm.nih.gov/nucleotide/XM_044854009.1?report=genbank&log$=nucltop&blast_rank=71&RID=J1040F1H013) |
| [*F. proliferatum*](https://www.ncbi.nlm.nih.gov/Taxonomy/Browser/wwwtax.cgi?id=948311) | 67.63 | [XP_044706679.1](https://www.ncbi.nlm.nih.gov/protein/XP_044706679.1?report=genbank&log$=prottop&blast_rank=51&RID=K120TSKZ013) |
| [*F. pseudograminearum*](https://www.ncbi.nlm.nih.gov/Taxonomy/Browser/wwwtax.cgi?id=101028) | 69.01 | [XP_009261241.1](https://www.ncbi.nlm.nih.gov/protein/XP_009261241.1?report=genbank&log$=prottop&blast_rank=64&RID=K120TSKZ013) |
| [*F. redolens*](https://www.ncbi.nlm.nih.gov/Taxonomy/Browser/wwwtax.cgi?id=48865) | 69.57 | [XM_046199994.1](https://www.ncbi.nlm.nih.gov/nucleotide/XM_046199994.1?report=genbank&log$=nucltop&blast_rank=79&RID=J1040F1H013) |
| [*F. sambucinum*](https://www.ncbi.nlm.nih.gov/Taxonomy/Browser/wwwtax.cgi?id=5128) | 69.36 | [XEV06865.1](https://www.ncbi.nlm.nih.gov/protein/XEV06865.1?report=genbank&log$=prottop&blast_rank=68&RID=K120TSKZ013) |
| [*F. solani*](https://www.ncbi.nlm.nih.gov/Taxonomy/Browser/wwwtax.cgi?id=169388) | 67.43 | [XM_065619716.1](https://www.ncbi.nlm.nih.gov/nucleotide/XM_065619716.1?report=genbank&log$=nucltop&blast_rank=82&RID=J1040F1H013) |
| [*F. subglutinans*](https://www.ncbi.nlm.nih.gov/Taxonomy/Browser/wwwtax.cgi?id=42677) | 68.40 | [XM_036685092.1](https://www.ncbi.nlm.nih.gov/nucleotide/XM_036685092.1?report=genbank&log$=nucltop&blast_rank=84&RID=J1040F1H013) |
| [*F. tjaetaba*](https://www.ncbi.nlm.nih.gov/Taxonomy/Browser/wwwtax.cgi?id=1567544) | 68.81 | [XM_037354500.1](https://www.ncbi.nlm.nih.gov/nucleotide/XM_037354500.1?report=genbank&log$=nucltop&blast_rank=86&RID=J1040F1H013) |
| [*F. vanettenii 77-13-4*](https://www.ncbi.nlm.nih.gov/Taxonomy/Browser/wwwtax.cgi?id=660122) | 69.00 | [XM_003049083.1](https://www.ncbi.nlm.nih.gov/nucleotide/XM_003049083.1?report=genbank&log$=nucltop&blast_rank=88&RID=J1040F1H013) |
| [*F. venenatum*](https://www.ncbi.nlm.nih.gov/Taxonomy/Browser/wwwtax.cgi?id=56646) | 68.60 | [XM_025725246.1](https://www.ncbi.nlm.nih.gov/nucleotide/XM_025725246.1?report=genbank&log$=nucltop&blast_rank=89&RID=J1040F1H013) |
| [*F. verticillioides*](https://www.ncbi.nlm.nih.gov/Taxonomy/Browser/wwwtax.cgi?id=117187) | 69.50 | [XP_018759186.1](https://www.ncbi.nlm.nih.gov/protein/XP_018759186.1?report=genbank&log$=prottop&blast_rank=97&RID=K120TSKZ013) |
| [*F. zealandicum*](https://www.ncbi.nlm.nih.gov/Taxonomy/Browser/wwwtax.cgi?id=569361) | 57.18 | [KAF4976459.1](https://www.ncbi.nlm.nih.gov/protein/KAF4976459.1?report=genbank&log$=prottop&blast_rank=100&RID=K120TSKZ013) |
| [***Histoplasma***](https://www.ncbi.nlm.nih.gov/Taxonomy/Browser/wwwtax.cgi?id=544711) |  |  |
| [*H. capsulatum*](https://www.ncbi.nlm.nih.gov/Taxonomy/Browser/wwwtax.cgi?id=544711) | 69.38 | [QSS59379.1](https://www.ncbi.nlm.nih.gov/protein/QSS59379.1?report=genbank&log$=prottop&blast_rank=1&RID=K12TSSGP016) |
| [***Marasmius***](https://www.ncbi.nlm.nih.gov/Taxonomy/Browser/wwwtax.cgi?id=181124) |  |  |
| [*M. oreades*](https://www.ncbi.nlm.nih.gov/Taxonomy/Browser/wwwtax.cgi?id=181124) | 78.85 | [XP_043002738.1](https://www.ncbi.nlm.nih.gov/protein/XP_043002738.1?report=genbank&log$=prottop&blast_rank=13&RID=K12XV216013) |
| [***Meyerozyma***](https://www.ncbi.nlm.nih.gov/Taxonomy/Browser/wwwtax.cgi?id=294746) |  |  |
| [*M. guilliermondii ATCC 6260*](https://www.ncbi.nlm.nih.gov/Taxonomy/Browser/wwwtax.cgi?id=294746) | 77.35 | [XM_001486519.1](https://www.ncbi.nlm.nih.gov/nucleotide/XM_001486519.1?report=genbank&log$=nucltop&blast_rank=1&RID=HZADP2WR016) |
| [***Microsporum***](https://www.ncbi.nlm.nih.gov/Taxonomy/Browser/wwwtax.cgi?id=554155) |  |  |
| [*M. canis CBS 113480*](https://www.ncbi.nlm.nih.gov/Taxonomy/Browser/wwwtax.cgi?id=554155) | 71.76 | [XM_002849340.1](https://www.ncbi.nlm.nih.gov/nucleotide/XM_002849340.1?report=genbank&log$=nucltop&blast_rank=1&RID=J10M1AYH013) |
| [***Nakaseomyces***](https://www.ncbi.nlm.nih.gov/Taxonomy/Browser/wwwtax.cgi?id=5478) |  |  |
| [*N. glabratus*](https://www.ncbi.nlm.nih.gov/Taxonomy/Browser/wwwtax.cgi?id=5478) | 87.03 | [XP_447590.1](https://www.ncbi.nlm.nih.gov/protein/XP_447590.1?report=genbank&log$=prottop&blast_rank=1&RID=K12Z6UMB016) |
| [***Neurospora***](https://www.ncbi.nlm.nih.gov/Taxonomy/Browser/wwwtax.cgi?id=5141) |  |  |
| [*N. crassa*](https://www.ncbi.nlm.nih.gov/Taxonomy/Browser/wwwtax.cgi?id=5141) | 67.92 | [XP_011393501.1](https://www.ncbi.nlm.nih.gov/protein/XP_011393501.1?report=genbank&log$=prottop&blast_rank=2&RID=K133MSPC016) |
| [*N. hispaniola*](https://www.ncbi.nlm.nih.gov/Taxonomy/Browser/wwwtax.cgi?id=588809) | 67.80 | [XM_062832877.1](https://www.ncbi.nlm.nih.gov/nucleotide/XM_062832877.1?report=genbank&log$=nucltop&blast_rank=4&RID=J10N83T4013) |
| [*N. tetrasperma FGSC 2508*](https://www.ncbi.nlm.nih.gov/Taxonomy/Browser/wwwtax.cgi?id=510951) | 68.93 | [XM_009853833.1](https://www.ncbi.nlm.nih.gov/nucleotide/XM_009853833.1?report=genbank&log$=nucltop&blast_rank=5&RID=J10N83T4013) |
| [*N. tetraspora*](https://www.ncbi.nlm.nih.gov/Taxonomy/Browser/wwwtax.cgi?id=94610) | 67.97 | [XM_062821761.1](https://www.ncbi.nlm.nih.gov/nucleotide/XM_062821761.1?report=genbank&log$=nucltop&blast_rank=6&RID=J10N83T4013) |
| ***Penicillium*** |  |  |
| [*P. alfredii*](https://www.ncbi.nlm.nih.gov/Taxonomy/Browser/wwwtax.cgi?id=1506179) | 71.07 | [XM_056652916.1](https://www.ncbi.nlm.nih.gov/nucleotide/XM_056652916.1?report=genbank&log$=nucltop&blast_rank=1&RID=J0J66A0J013) |
| [*P. angulare*](https://www.ncbi.nlm.nih.gov/Taxonomy/Browser/wwwtax.cgi?id=116970) | 68.95 | [XM_056917383.1](https://www.ncbi.nlm.nih.gov/nucleotide/XM_056917383.1?report=genbank&log$=nucltop&blast_rank=4&RID=J0J66A0J013) |
| [*P. antarcticum*](https://www.ncbi.nlm.nih.gov/Taxonomy/Browser/wwwtax.cgi?id=416450) | 65.72 | [XM_058460028.1](https://www.ncbi.nlm.nih.gov/nucleotide/XM_058460028.1?report=genbank&log$=nucltop&blast_rank=5&RID=J0J66A0J013) |
| [*P. argentinense*](https://www.ncbi.nlm.nih.gov/Taxonomy/Browser/wwwtax.cgi?id=1131581) | 68.18 | [XM_056617858.1](https://www.ncbi.nlm.nih.gov/nucleotide/XM_056617858.1?report=genbank&log$=nucltop&blast_rank=8&RID=J0J66A0J013) |
| [*P. arizonense*](https://www.ncbi.nlm.nih.gov/Taxonomy/Browser/wwwtax.cgi?id=1835702) | 66.51 | [XM_022627738.1](https://www.ncbi.nlm.nih.gov/nucleotide/XM_022627738.1?report=genbank&log$=nucltop&blast_rank=9&RID=J0J66A0J013) |
| [*P. atrosanguineum*](https://www.ncbi.nlm.nih.gov/Taxonomy/Browser/wwwtax.cgi?id=1132637) | 65.54 | [XM_056884195.1](https://www.ncbi.nlm.nih.gov/nucleotide/XM_056884195.1?report=genbank&log$=nucltop&blast_rank=11&RID=J0J66A0J013) |
| [*P. bovifimosum*](https://www.ncbi.nlm.nih.gov/Taxonomy/Browser/wwwtax.cgi?id=126998) | 68.69 | [XM_056661232.1](https://www.ncbi.nlm.nih.gov/nucleotide/XM_056661232.1?report=genbank&log$=nucltop&blast_rank=13&RID=J0J66A0J013) |
| [*P. brevicompactum*](https://www.ncbi.nlm.nih.gov/Taxonomy/Browser/wwwtax.cgi?id=5074) | 65.13 | [XM_056952131.1](https://www.ncbi.nlm.nih.gov/nucleotide/XM_056952131.1?report=genbank&log$=nucltop&blast_rank=14&RID=J0J66A0J013) |
| [*P. canariense*](https://www.ncbi.nlm.nih.gov/Taxonomy/Browser/wwwtax.cgi?id=189055) | 69.72 | [XM_056684609.1](https://www.ncbi.nlm.nih.gov/nucleotide/XM_056684609.1?report=genbank&log$=nucltop&blast_rank=16&RID=J0J66A0J013) |
| [*P. cataractarum*](https://www.ncbi.nlm.nih.gov/Taxonomy/Browser/wwwtax.cgi?id=2100454) | 71.14 | [XM_056703582.1](https://www.ncbi.nlm.nih.gov/nucleotide/XM_056703582.1?report=genbank&log$=nucltop&blast_rank=21&RID=J0J66A0J013) |
| [*P. chrysogenum*](https://www.ncbi.nlm.nih.gov/Taxonomy/Browser/wwwtax.cgi?id=5076) | 65.20 | [XM_056713492.1](https://www.ncbi.nlm.nih.gov/nucleotide/XM_056713492.1?report=genbank&log$=nucltop&blast_rank=23&RID=J0J66A0J013) |
| [*P. cinerascens*](https://www.ncbi.nlm.nih.gov/Taxonomy/Browser/wwwtax.cgi?id=70096) | 65.60 | [XM_058457381.1](https://www.ncbi.nlm.nih.gov/nucleotide/XM_058457381.1?report=genbank&log$=nucltop&blast_rank=24&RID=J0J66A0J013) |
| [*P. citrinum*](https://www.ncbi.nlm.nih.gov/Taxonomy/Browser/wwwtax.cgi?id=5077) | 67.08 | [XM_056641455.1](https://www.ncbi.nlm.nih.gov/nucleotide/XM_056641455.1?report=genbank&log$=nucltop&blast_rank=25&RID=J0J66A0J013) |
| [*P. concentricum*](https://www.ncbi.nlm.nih.gov/Taxonomy/Browser/wwwtax.cgi?id=293559) | 68.75 | [XM_056717844.1](https://www.ncbi.nlm.nih.gov/nucleotide/XM_056717844.1?report=genbank&log$=nucltop&blast_rank=31&RID=J0J66A0J013) |
| [*P. coprophilum*](https://www.ncbi.nlm.nih.gov/Taxonomy/Browser/wwwtax.cgi?id=36646) | 67.86 | [XM_056678819.1](https://www.ncbi.nlm.nih.gov/nucleotide/XM_056678819.1?report=genbank&log$=nucltop&blast_rank=33&RID=J0J66A0J013) |
| [*P. cosmopolitanum*](https://www.ncbi.nlm.nih.gov/Taxonomy/Browser/wwwtax.cgi?id=1131564) | 66.19 | [XM_056630264.1](https://www.ncbi.nlm.nih.gov/nucleotide/XM_056630264.1?report=genbank&log$=nucltop&blast_rank=34&RID=J0J66A0J013) |
| [*P. crustosum*](https://www.ncbi.nlm.nih.gov/Taxonomy/Browser/wwwtax.cgi?id=36656) | 69.78 | [XM_056868632.1](https://www.ncbi.nlm.nih.gov/nucleotide/XM_056868632.1?report=genbank&log$=nucltop&blast_rank=37&RID=J0J66A0J013) |
| [*P. daleae*](https://www.ncbi.nlm.nih.gov/Taxonomy/Browser/wwwtax.cgi?id=63821) | 72.59 | [XM_056906206.1](https://www.ncbi.nlm.nih.gov/nucleotide/XM_056906206.1?report=genbank&log$=nucltop&blast_rank=38&RID=J0J66A0J013) |
| [*P. diatomitis*](https://www.ncbi.nlm.nih.gov/Taxonomy/Browser/wwwtax.cgi?id=2819901) | 68.24 | [XM_056930155.1](https://www.ncbi.nlm.nih.gov/nucleotide/XM_056930155.1?report=genbank&log$=nucltop&blast_rank=40&RID=J0J66A0J013) |
| [*P. digitatum*](https://www.ncbi.nlm.nih.gov/Taxonomy/Browser/wwwtax.cgi?id=36651) | 73.60 | [XM_014678911.1](https://www.ncbi.nlm.nih.gov/nucleotide/XM_014678911.1?report=genbank&log$=nucltop&blast_rank=41&RID=J0J66A0J013) |
| [*P. expansum*](https://www.ncbi.nlm.nih.gov/Taxonomy/Browser/wwwtax.cgi?id=27334) | 68.06 | [XM_016741737.1](https://www.ncbi.nlm.nih.gov/nucleotide/XM_016741737.1?report=genbank&log$=nucltop&blast_rank=46&RID=J0J66A0J013) |
| [*P. griseofulvum*](https://www.ncbi.nlm.nih.gov/Taxonomy/Browser/wwwtax.cgi?id=5078) | 66.40 | [XM_040793848.1](https://www.ncbi.nlm.nih.gov/nucleotide/XM_040793848.1?report=genbank&log$=nucltop&blast_rank=49&RID=J0J66A0J013) |
| [*P. hispanicum*](https://www.ncbi.nlm.nih.gov/Taxonomy/Browser/wwwtax.cgi?id=1080232) | 70.10 | [XM_056947615.1](https://www.ncbi.nlm.nih.gov/nucleotide/XM_056947615.1?report=genbank&log$=nucltop&blast_rank=51&RID=J0J66A0J013) |
| [*P. hordei*](https://www.ncbi.nlm.nih.gov/Taxonomy/Browser/wwwtax.cgi?id=40994) | 64.69 | [XM_056897188.1](https://www.ncbi.nlm.nih.gov/nucleotide/XM_056897188.1?report=genbank&log$=nucltop&blast_rank=53&RID=J0J66A0J013) |
| [*P. lagena*](https://www.ncbi.nlm.nih.gov/Taxonomy/Browser/wwwtax.cgi?id=94218) | 65.26 | [XM_056975341.1](https://www.ncbi.nlm.nih.gov/nucleotide/XM_056975341.1?report=genbank&log$=nucltop&blast_rank=54&RID=J0J66A0J013) |
| [*P. longicatenatum*](https://www.ncbi.nlm.nih.gov/Taxonomy/Browser/wwwtax.cgi?id=1561947) | 66.47 | [XM_057114105.1](https://www.ncbi.nlm.nih.gov/nucleotide/XM_057114105.1?report=genbank&log$=nucltop&blast_rank=55&RID=J0J66A0J013) |
| [*P. maclennaniae*](https://www.ncbi.nlm.nih.gov/Taxonomy/Browser/wwwtax.cgi?id=1343394) | 68.06 | [XM_056969565.1](https://www.ncbi.nlm.nih.gov/nucleotide/XM_056969565.1?report=genbank&log$=nucltop&blast_rank=56&RID=J0J66A0J013) |
| [*P. macrosclerotiorum*](https://www.ncbi.nlm.nih.gov/Taxonomy/Browser/wwwtax.cgi?id=303699) | 67.13 | [XM_057075686.1](https://www.ncbi.nlm.nih.gov/nucleotide/XM_057075686.1?report=genbank&log$=nucltop&blast_rank=57&RID=J0J66A0J013) |
| [*P. malachiteum*](https://www.ncbi.nlm.nih.gov/Taxonomy/Browser/wwwtax.cgi?id=1324776) | 66.13 | [XM_057084100.1](https://www.ncbi.nlm.nih.gov/nucleotide/XM_057084100.1?report=genbank&log$=nucltop&blast_rank=58&RID=J0J66A0J013) |
| [*P. manginii*](https://www.ncbi.nlm.nih.gov/Taxonomy/Browser/wwwtax.cgi?id=203109) | 67.60 | [XM_057100730.1](https://www.ncbi.nlm.nih.gov/nucleotide/XM_057100730.1?report=genbank&log$=nucltop&blast_rank=59&RID=J0J66A0J013) |
| [*P. mononematosum*](https://www.ncbi.nlm.nih.gov/Taxonomy/Browser/wwwtax.cgi?id=268346) | 65.00 | [XM_057294207.1](https://www.ncbi.nlm.nih.gov/nucleotide/XM_057294207.1?report=genbank&log$=nucltop&blast_rank=61&RID=J0J66A0J013) |
| [*P. nucicola*](https://www.ncbi.nlm.nih.gov/Taxonomy/Browser/wwwtax.cgi?id=1850975) | 65.41 | [XM_057123149.1](https://www.ncbi.nlm.nih.gov/nucleotide/XM_057123149.1?report=genbank&log$=nucltop&blast_rank=62&RID=J0J66A0J013) |
| [*P. odoratum*](https://www.ncbi.nlm.nih.gov/Taxonomy/Browser/wwwtax.cgi?id=1167516) | 65.15 | [XM_057141094.1](https://www.ncbi.nlm.nih.gov/nucleotide/XM_057141094.1?report=genbank&log$=nucltop&blast_rank=64&RID=J0J66A0J013) |
| [*P. oxalicum*](https://www.ncbi.nlm.nih.gov/Taxonomy/Browser/wwwtax.cgi?id=69781) | 66.86 | [XM_050109631.1](https://www.ncbi.nlm.nih.gov/nucleotide/XM_050109631.1?report=genbank&log$=nucltop&blast_rank=65&RID=J0J66A0J013) |
| [*P. paradoxum*](https://www.ncbi.nlm.nih.gov/Taxonomy/Browser/wwwtax.cgi?id=176176) | 65.11 | [XM_057172402.1](https://www.ncbi.nlm.nih.gov/nucleotide/XM_057172402.1?report=genbank&log$=nucltop&blast_rank=67&RID=J0J66A0J013) |
| [*P. psychrosexuale*](https://www.ncbi.nlm.nih.gov/Taxonomy/Browser/wwwtax.cgi?id=1002107) | 65.50 | [XM_057186317.1](https://www.ncbi.nlm.nih.gov/nucleotide/XM_057186317.1?report=genbank&log$=nucltop&blast_rank=71&RID=J0J66A0J013) |
| [*P. pulvis*](https://www.ncbi.nlm.nih.gov/Taxonomy/Browser/wwwtax.cgi?id=1562058) | 67.44 | [XM_057070565.1](https://www.ncbi.nlm.nih.gov/nucleotide/XM_057070565.1?report=genbank&log$=nucltop&blast_rank=73&RID=J0J66A0J013) |
| [*P. riverlandense*](https://www.ncbi.nlm.nih.gov/Taxonomy/Browser/wwwtax.cgi?id=1903569) | 71.72 | [XM_057193030.1](https://www.ncbi.nlm.nih.gov/nucleotide/XM_057193030.1?report=genbank&log$=nucltop&blast_rank=75&RID=J0J66A0J013) |
| [*P. robsamsonii*](https://www.ncbi.nlm.nih.gov/Taxonomy/Browser/wwwtax.cgi?id=1792511) | 65.54 | [XM_057226661.1](https://www.ncbi.nlm.nih.gov/nucleotide/XM_057226661.1?report=genbank&log$=nucltop&blast_rank=77&RID=J0J66A0J013) |
| [*P. rubens*](https://www.ncbi.nlm.nih.gov/Taxonomy/Browser/wwwtax.cgi?id=1108849) | 65.18 | [XM_002557500.1](https://www.ncbi.nlm.nih.gov/nucleotide/XM_002557500.1?report=genbank&log$=nucltop&blast_rank=83&RID=J0J66A0J013) |
| [*P. samsonianum*](https://www.ncbi.nlm.nih.gov/Taxonomy/Browser/wwwtax.cgi?id=1882272) | 65.20 | [XM_057280563.1](https://www.ncbi.nlm.nih.gov/nucleotide/XM_057280563.1?report=genbank&log$=nucltop&blast_rank=84&RID=J0J66A0J013) |
| [*P. solitum*](https://www.ncbi.nlm.nih.gov/Taxonomy/Browser/wwwtax.cgi?id=60172) | 63.49 | [XM_040958429.2](https://www.ncbi.nlm.nih.gov/nucleotide/XM_040958429.2?report=genbank&log$=nucltop&blast_rank=86&RID=J0J66A0J013) |
| [*P. soppii*](https://www.ncbi.nlm.nih.gov/Taxonomy/Browser/wwwtax.cgi?id=69789) | 67.31 | [XM_057238597.1](https://www.ncbi.nlm.nih.gov/nucleotide/XM_057238597.1?report=genbank&log$=nucltop&blast_rank=87&RID=J0J66A0J013) |
| [*P. subrubescens*](https://www.ncbi.nlm.nih.gov/Taxonomy/Browser/wwwtax.cgi?id=1316194) | 71.54 | [XM_057150233.1](https://www.ncbi.nlm.nih.gov/nucleotide/XM_057150233.1?report=genbank&log$=nucltop&blast_rank=91&RID=J0J66A0J013) |
| [*P. taxi*](https://www.ncbi.nlm.nih.gov/Taxonomy/Browser/wwwtax.cgi?id=168475) | 67.69 | [XM_057203558.1](https://www.ncbi.nlm.nih.gov/nucleotide/XM_057203558.1?report=genbank&log$=nucltop&blast_rank=92&RID=J0J66A0J013) |
| [*P. verhagenii*](https://www.ncbi.nlm.nih.gov/Taxonomy/Browser/wwwtax.cgi?id=1562060) | 65.56 | [XM_057159758.1](https://www.ncbi.nlm.nih.gov/nucleotide/XM_057159758.1?report=genbank&log$=nucltop&blast_rank=94&RID=J0J66A0J013) |
| [*P. verrucosum*](https://www.ncbi.nlm.nih.gov/Taxonomy/Browser/wwwtax.cgi?id=60171) | 67.65 | [XM_057221584.1](https://www.ncbi.nlm.nih.gov/nucleotide/XM_057221584.1?report=genbank&log$=nucltop&blast_rank=96&RID=J0J66A0J013) |
| [*P. vulpinum*](https://www.ncbi.nlm.nih.gov/Taxonomy/Browser/wwwtax.cgi?id=29845) | 68.04 | [XM_057248125.1](https://www.ncbi.nlm.nih.gov/nucleotide/XM_057248125.1?report=genbank&log$=nucltop&blast_rank=98&RID=J0J66A0J013) |
| [*P. waksmanii*](https://www.ncbi.nlm.nih.gov/Taxonomy/Browser/wwwtax.cgi?id=69791) | 66.61 | [XM_057272213.1](https://www.ncbi.nlm.nih.gov/nucleotide/XM_057272213.1?report=genbank&log$=nucltop&blast_rank=99&RID=J0J66A0J013) |
| [***Pichia***](https://www.ncbi.nlm.nih.gov/Taxonomy/Browser/wwwtax.cgi?id=4909) |  |  |
| *P. californica* | 77.17 | [KAG0689811.1](https://www.ncbi.nlm.nih.gov/protein/KAG0689811.1?report=genbank&log$=prottop&blast_rank=1&RID=K139WDWP016) |
| *P. inconspicua* | 76.95 | [TID30977.1](https://www.ncbi.nlm.nih.gov/protein/TID30977.1?report=genbank&log$=prottop&blast_rank=12&RID=K139WDWP016) |
| *P. kluyveri* | 77.52 | [GMM47827.1](https://www.ncbi.nlm.nih.gov/protein/GMM47827.1?report=genbank&log$=prottop&blast_rank=20&RID=K139WDWP016) |
| [*P. kudriavzevii*](https://www.ncbi.nlm.nih.gov/Taxonomy/Browser/wwwtax.cgi?id=4909) | 77.81 | [XP_029322425.1](https://www.ncbi.nlm.nih.gov/protein/XP_029322425.1?report=genbank&log$=prottop&blast_rank=29&RID=K139WDWP016) |
| *P. membranifaciens* | 75.72 | [XP_019017124.1](https://www.ncbi.nlm.nih.gov/protein/XP_019017124.1?report=genbank&log$=prottop&blast_rank=68&RID=K139WDWP016) |
| [***Saccharomyces***](https://www.ncbi.nlm.nih.gov/Taxonomy/Browser/wwwtax.cgi?id=4932) |  |  |
| [*S. cerevisiae*](https://www.ncbi.nlm.nih.gov/Taxonomy/Browser/wwwtax.cgi?id=4932) | 100.00 | [CP093603.1](https://www.ncbi.nlm.nih.gov/nucleotide/CP093603.1?report=genbank&log$=nucltop&blast_rank=1&RID=J0J7PH2E013) |
| [***Trichoderma***](https://www.ncbi.nlm.nih.gov/Taxonomy/Browser/wwwtax.cgi?id=173218) |  |  |
| [*T. aggressivum f. europaeum*](https://www.ncbi.nlm.nih.gov/Taxonomy/Browser/wwwtax.cgi?id=173218) | 66.49 | [XM_062904604.1](https://www.ncbi.nlm.nih.gov/nucleotide/XM_062904604.1?report=genbank&log$=nucltop&blast_rank=1&RID=J106BG2H013) |
| [*T. asperellum*](https://www.ncbi.nlm.nih.gov/Taxonomy/Browser/wwwtax.cgi?id=101201) | 65.12 | [XM_024901111.2](https://www.ncbi.nlm.nih.gov/nucleotide/XM_024901111.2?report=genbank&log$=nucltop&blast_rank=2&RID=J106BG2H013) |
| [*T. atroviride*](https://www.ncbi.nlm.nih.gov/Taxonomy/Browser/wwwtax.cgi?id=63577) | 70.25 | [XM_014088124.2](https://www.ncbi.nlm.nih.gov/nucleotide/XM_014088124.2?report=genbank&log$=nucltop&blast_rank=5&RID=J106BG2H013) |
| [*T. breve*](https://www.ncbi.nlm.nih.gov/Taxonomy/Browser/wwwtax.cgi?id=2034170) | 65.77 | [XM_056168136.1](https://www.ncbi.nlm.nih.gov/nucleotide/XM_056168136.1?report=genbank&log$=nucltop&blast_rank=7&RID=J106BG2H013) |
| [*T. citrinoviride*](https://www.ncbi.nlm.nih.gov/Taxonomy/Browser/wwwtax.cgi?id=58853) | 66.23 | [XP_024753357.1](https://www.ncbi.nlm.nih.gov/protein/XP_024753357.1?report=genbank&log$=prottop&blast_rank=29&RID=K13JAY33013) |
| [*T. gamsii*](https://www.ncbi.nlm.nih.gov/Taxonomy/Browser/wwwtax.cgi?id=398673) | 72.03 | [XM_018803561.1](https://www.ncbi.nlm.nih.gov/nucleotide/XM_018803561.1?report=genbank&log$=nucltop&blast_rank=10&RID=J106BG2H013) |
| [*T. harzianum CBS 226.95*](https://www.ncbi.nlm.nih.gov/Taxonomy/Browser/wwwtax.cgi?id=983964) | 70.05 | [XM_024922578.1](https://www.ncbi.nlm.nih.gov/nucleotide/XM_024922578.1?report=genbank&log$=nucltop&blast_rank=11&RID=J106BG2H013) |
| [*T. reesei*](https://www.ncbi.nlm.nih.gov/Taxonomy/Browser/wwwtax.cgi?id=51453) | 66.32 | [XP_006968225.1](https://www.ncbi.nlm.nih.gov/protein/XP_006968225.1?report=genbank&log$=prottop&blast_rank=78&RID=K13JAY33013) |
| [*T. virens*](https://www.ncbi.nlm.nih.gov/Taxonomy/Browser/wwwtax.cgi?id=29875) | 66.05 | [XP_013960809.1](https://www.ncbi.nlm.nih.gov/protein/XP_013960809.1?report=genbank&log$=prottop&blast_rank=95&RID=K13JAY33013) |
| [***Ustilago***](https://www.ncbi.nlm.nih.gov/Taxonomy/Browser/wwwtax.cgi?id=307758) |  |  |
| [*U. bromivora*](https://www.ncbi.nlm.nih.gov/Taxonomy/Browser/wwwtax.cgi?id=307758) | 64.52 | [SAM81393.1](https://www.ncbi.nlm.nih.gov/protein/SAM81393.1?report=genbank&log$=prottop&blast_rank=1&RID=K13RS11U013) |
| [*U. hordei*](https://www.ncbi.nlm.nih.gov/Taxonomy/Browser/wwwtax.cgi?id=120017) | 66.16 | [XM_041555184.1](https://www.ncbi.nlm.nih.gov/nucleotide/XM_041555184.1?report=genbank&log$=nucltop&blast_rank=3&RID=J0JTXDJ5016) |
| ***Xylaria*** |  |  |
| [*X. bambusicola*](https://www.ncbi.nlm.nih.gov/Taxonomy/Browser/wwwtax.cgi?id=326684) | 67.25 | [XM_047969112.1](https://www.ncbi.nlm.nih.gov/nucleotide/XM_047969112.1?report=genbank&log$=nucltop&blast_rank=1&RID=J0JUP5VF016) |
| [***Yarrowia***](https://www.ncbi.nlm.nih.gov/Taxonomy/Browser/wwwtax.cgi?id=4952) |  |  |
| [*Y. lipolytica*](https://www.ncbi.nlm.nih.gov/Taxonomy/Browser/wwwtax.cgi?id=4952) | 82.72 | [QNQ00095.1](https://www.ncbi.nlm.nih.gov/protein/QNQ00095.1?report=genbank&log$=prottop&blast_rank=1&RID=K13YTD39016) |

**Supplementary Table 5** Analysis of indole receptor homologs in *E. cloacae*

| **Bacteria** | **The identified receptor** | **Homologs** | **Description** | **Identity (%)** |
| --- | --- | --- | --- | --- |
|  |  |  |  |  |
| *Escherichia coli* | BaeS | ECL_03405 | signal transduction histidine-protein kinase BaeS | 76.66 |
|  | BaeR | ECL_03406 | DNA-binding transcriptional regulator BaeR | 82.56 |
|  | CpxA | ECL_05065 | two-component system, OmpR family, sensor histidine kinase CpxA | 83.54 |
|  | CpxR | ECL_05064 | two-component system, OmpR family, response regulator CpxR | 83.91 |
| *Lysobacter enzymogenes* | QseC | ECL_04344 | two-component system, OmpR family, sensor histidine kinase QseC | 68.7 |
| *Pseudomonas fluorescens* | EmhR | - | - | - |
| *Stigmatella aurantiaca* | PK | ECL_01444 | pyruvate kinase | 33.65 |
| *Vibrio cholerae* | DksA | ECL_00949 | DnaK transcriptional regulator DksA | 38.95 |
| *Enterohemorrhagic E. coli* | IsrR | - | - | - |
| *Acinetobacter baumannii* | AbiR | - | - | - |
